# An autonomous implantable device for the prevention of death from opioid overdose

**DOI:** 10.1126/sciadv.adr3567

**Published:** 2024-10-23

**Authors:** Joanna L. Ciatti, Abraham Vázquez-Guardado, Victoria E. Brings, Jihun Park, Brian Ruyle, Rebecca A. Ober, Alicia J. McLuckie, Michael R. Talcott, Emily A. Carter, Amy R. Burrell, Rebecca A. Sponenburg, Jacob Trueb, Prashant Gupta, Joohee Kim, Raudel Avila, Minho Seong, Richard A. Slivicki, Melanie A. Kaplan, Bryan Villalpando-Hernandez, Nicolas Massaly, Michael C. Montana, Mitchell Pet, Yonggang Huang, Jose A. Morón, Robert W. Gereau, John A. Rogers

**Affiliations:** ^1^ Department of Materials Science and Engineering, Northwestern University, Evanston, IL 60208, USA.; ^2^ Querrey Simpson Institute for Bioelectronics, Northwestern University, Evanston, IL 60208, USA.; ^3^ Department of Electrical and Computer Engineering, North Carolina State University, Raleigh, NC 27606, USA.; ^4^ Department of Anesthesiology, Washington University School of Medicine, St. Louis, MO 63110, USA.; ^5^ Washington University Pain Center, Washington University School of Medicine, St. Louis, MO 63110, USA.; ^6^ Department of Neuroscience, Washington University, St. Louis, MO 63110, USA.; ^7^ Center for Comparative Medicine, Northwestern University, Evanston, IL 60208, USA.; ^8^ Department of Pathology, Northwestern University Feinberg School of Medicine, Chicago, IL 60611, USA.; ^9^ Division of Cardiology, Washington University School of Medicine, St. Louis, MO 63110, USA.; ^10^ Chemistry of Life Processes Institute (Quantitative Bio-element Imaging Center), Northwestern University, Evanston, IL 60208, USA.; ^11^ Department of Mechanical Engineering, Northwestern University, Evanston, IL 60208, USA.; ^12^ Division of Plastic and Reconstructive Surgery, Washington University School of Medicine, St. Louis, MO 63110, USA.; ^13^ Department of Civil and Environmental Engineering, Northwestern University, Evanston, IL 60208, USA.; ^14^ Department of Biomedical Engineering, Washington University, St. Louis, MO 63110, USA.; ^15^ Department of Biomedical Engineering, Northwestern University, Evanston, IL 60208, USA.; ^16^ Department of Neurological Surgery, Northwestern University Feinberg School of Medicine, Chicago, IL 60611, USA.

## Abstract

Opioid overdose accounts for nearly 75,000 deaths per year in the United States, now a leading cause of mortality among young people aged 18 to 45 years. At overdose levels, opioid-induced respiratory depression becomes fatal without the administration of naloxone within minutes. Currently, overdose survival relies on bystander intervention, requiring a nearby person to find the overdosed individual and have immediate access to naloxone to administer. To circumvent the bystander requirement, we developed the Naloximeter: a class of life-saving implantable devices that autonomously detect and treat overdose while simultaneously contacting first responders. We present three Naloximeter platforms, for fundamental research and clinical translation, all equipped with optical sensors, drug delivery mechanisms, and a supporting ecosystem of technology to counteract opioid-induced respiratory depression. In small and large animal studies, the Naloximeter rescues from otherwise fatal opioid overdose within minutes. This work introduces life-changing, clinically translatable technologies that can broadly benefit a susceptible population recovering from opioid use disorder.

## Teaser

A lifesaving device automatically detects opioid overdose and rapidly responds with naloxone, averting overdose fatalities.

## INTRODUCTION

Opioids have been used for medicinal purposes for centuries and remain the primary analgesic to treat moderate to severe acute pain (*1*, *2*). Opioid use disorder (OUD), characterized by persistent opioid usage despite its adverse consequences, is a chronic relapse disorder and is one of the major contributors to global disease burden, with more than 40 million people dependent on opioids worldwide in 2017 (*3*–*5*). A rise in drug-involved overdose deaths in the United States has coincided with the widespread availability of synthetic opioids, e.g., fentanyl, which accounted for approximately 75,000 deaths in 2023, roughly 70% of all drug overdose deaths (*6*–*9*). The age-adjusted drug overdose mortality rate has nearly tripled over the past 10 years (*10*). Medically supervised withdrawal, or detoxification, is the standard of care for OUD treatment (*4*). This population has, however, a risk of fatal overdose that is elevated by 10 to 16 times within the first several months following a period of sobriety (*11*–*15*). Further, 5 to 10% of patients hospitalized for a drug overdose will die within 1 year of discharge (*16*, *17*). Therefore, high-risk individuals recovering from OUD require solutions to prevent fatalities in the event of relapse.

Opioid-induced respiratory depression (OIRD) is a dose-dependent phenomenon that can progress to complete apnea without intervention. This response can lead to overdose fatality due to neuronal damage caused by disruption of oxygen supply to the brain and other vital organs (*18*–*21*). Thus, the timely detection of overdose is critical to minimizing the risk of death. Current treatment for overdose involves administration of the rescue drug naloxone (NLX), an opioid receptor antagonist that temporarily reverses the effect of opioids and restores breathing (*19*, *22*). Overdose reversal with NLX is highly effective; however, deployment to an acutely overdosing patient requires timely identification of overdose, the immediate availability of NLX, and proper training of the bystander to administer (*23*, *24*). Given that overdoses often occur when the patient is alone, hypoxic brain injury and death are the most common outcomes (*25*, *26*). Public-health solutions including supervised injection facilities (*27*, *28*), virtual overdose monitoring systems (*29*–*33*), or fixed-location overdose detection devices (*34*–*36*) are of growing interest, but these solutions are slow to implement and costly, and, most importantly, still require human intervention to deliver NLX.

To address this challenge, we envisioned a closed-loop rescue device for the rapid detection and autonomous treatment of opioid overdose, which obviates the need for bystander intervention. A few devices designed to detect overdose and deliver NLX have been proposed recently, but their bulky form factors, impracticality, and lack of in vivo validation studies suggest limited real-world relevance (*37*–*39*). Wearable devices for overdose detection are also of interest (*40*–*43*), but, as mentioned, they lack autonomous rescue capabilities. We introduce a device platform that includes physiological sensors with several options for automated drug delivery, power supply, and wireless communications. An accompanying software system supports real-time data analytics and closed-loop control. The technology, referred to in the following as a Naloximeter, addresses both research and clinical applications, as demonstrated in benchtop investigations and in vivo demonstrations of automatic overdose rescue in rodent and porcine models. The design choices emphasize engineering simplicity and capability for clinical translation, where large animal demonstrations highlight its potential life-saving benefits for humans.

## RESULTS

### Implantable device for automated closed-loop rescue from opioid overdose

**Fig. 1.**
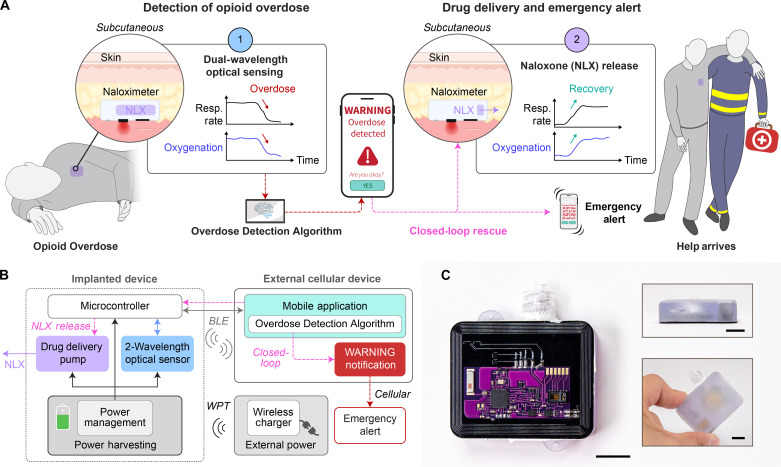
Autonomous operational scheme of Naloximeter for continuous sensing, triggered drug delivery and rescue from opioid overdose. (**A**) Schematic illustration of a scenario in which an implanted device (Naloximeter) detects respiratory depression leading to hypoxia, a signature of opioid overdose, delivers the rescue drug NLX, and sends an emergency alert to first responders. Created with BioRender.com. (**B**) Operational diagram including transdermal wireless power transfer, bidirectional communication via BLE protocols, and cellular communication that delivers an emergency alert. (**C**) Optical images of a Naloximeter; inset: side (top) and top view (bottom) of the device. Scale bars, 1 cm.

The Naloximeter is a subcutaneously implanted device that uses (1) a dual-wavelength optical sensor for measurements of local tissue oxygenation (StO_2_) to detect physiological signs of an overdose and (2) an automated drug delivery system to execute a closed-loop, rapid rescue response (Fig. 1A). The optical sensor operates at red (660 nm) and near-infrared (880 nm) wavelengths to yield time-series data that are analyzed with a multivariable algorithm for overdose detection, including calculations of StO_2_, based on established methods of near-infrared spectroscopy (*44*), along with other features in the data. The system microcontroller communicates via Bluetooth Low Energy (BLE) protocols to a cellular-enabled device (phone or tablet) with a custom mobile application to control the overall operation.

The closed-loop functionality is a response cascade that begins with the continuous analysis of data from the optical sensor to monitor for signatures of an overdose, followed by rapid NLX delivery and transmission of an emergency alert to first responders. A warning notification on the cellular device allows the user to abort the rescue process as a fail-safe mechanism in the case of a falsely detected overdose event. In an embodiment designed for large animals and ultimately humans, a lithium polymer (LiPo) battery capable of wireless recharging at the medical frequency band (13.56 MHz) via magnetic induction serves as the power supply (Fig. 1B). A battery-free, near-field communication (NFC) version designed for research with small animal models exploits magnetic induction for wireless communication and continuous power delivery. Additional details on the electronics, software, and related characterizations are in Supplementary text, figs. S1 to S7, and table S1. Figure 1C presents images of a Naloximeter device for large animals; the areal footprint (42 mm by 34 mm by 13 mm) is approximately 75% the size of a commercial pacemaker (fig. S8).

**Fig. 2.**
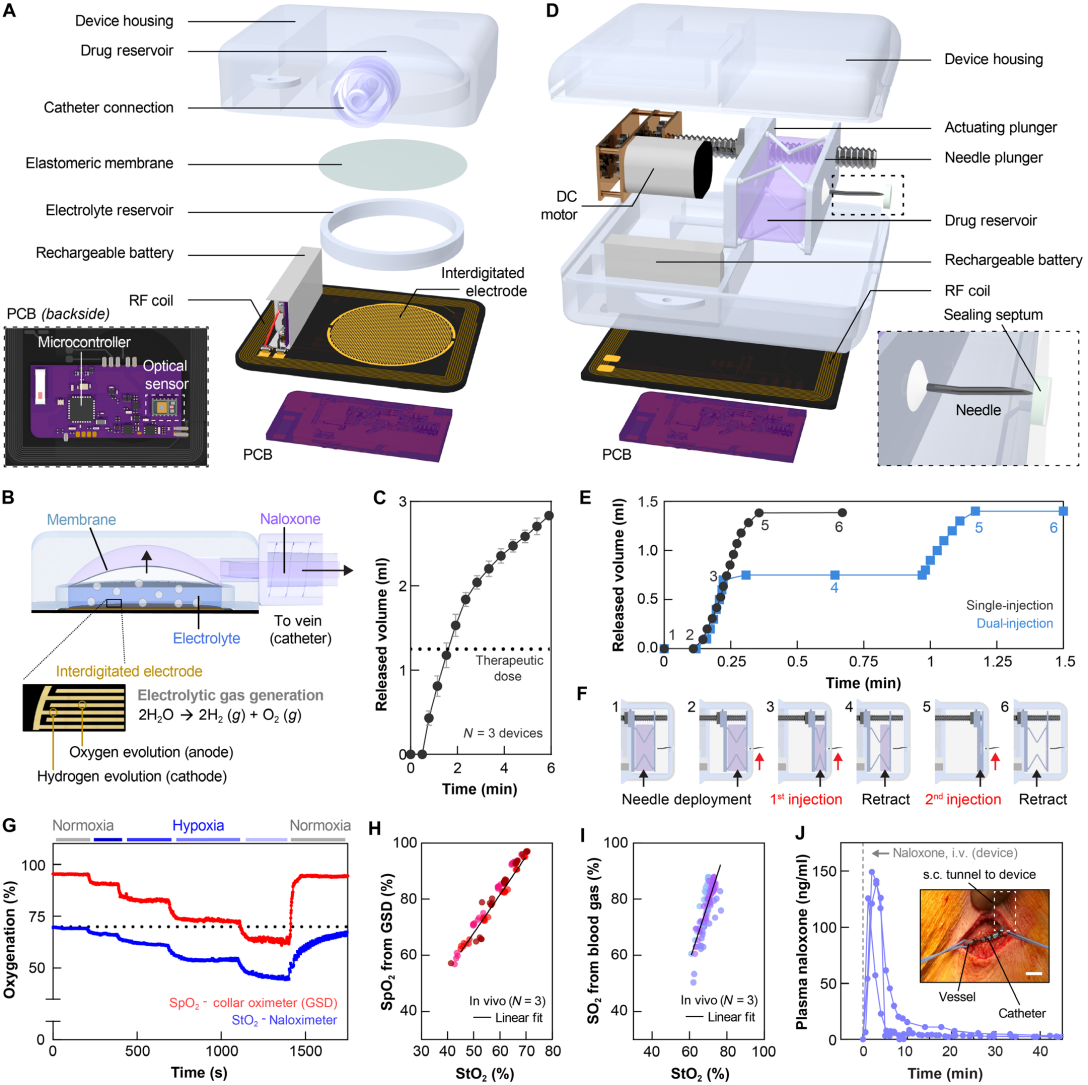
Device design and in-vivo validation studies. (**A**) Exploded view illustration of a Naloximeter for intravenous drug delivery via catheter and electrolytic pump; inset: PCB layout containing the dual-wavelength optical sensor, mounted on the backside of the assembled pump device. (**B**) Cross-sectional illustration showing deformation of a flexible membrane under pressure from gas generated via electrolytic water splitting. This process drives NLX through a catheter port to a vein. (**C**) Drug release from the electrolytic device, *N* = 3 devices. (**D**) Exploded view illustration of a Naloximeter for drug injection via a needle and motor-driven actuator; inset: Huber needle and polytetrafluoroethylene septum at the outlet of the housing. (**E**) Drug release from the injector device in single- or dual-injection mode, *N* = 3 devices. (**F**) Schematic illustrations of dual-injection operation, namely: needle deployment (1–2), delivery of first dose (3), retraction (4), delivery of second dose (5), and final retraction once empty (6). (**G**) Validation of the dual-wavelength optical sensor for determining StO_2_ via sequential exposure to hypoxic gas mixtures (21, 14, 12, 10, 8, and 21% O_2_, denoted as colored bars) in a rodent model; SpO_2_ data collected using a GSD (MouseOx collar). (**H**) Correlation plot for SpO_2_ (GSD) and StO_2_ (Naloximeter) resulting from rodent hypoxia validation studies (*R*^2^ = 0.95). Colors signify individual animals. (**I**) Correlation plot for SO_2_ from blood gases and StO_2_ from the dual-wavelength optical sensor validated in porcine hypoxia studies (*R*^2^ = 0.65). Colors signify individual devices. (**J**) Pharmacokinetics of NLX delivered using the intravenous device in a porcine model, dose: 3 ml (2.7 mg of NLX), *N* = 3 animals. Inset: Optical image of a catheter tunneled subcutaneously from a device and secured in the jugular vein; scale bar, 1 cm. i.v., intravenous. s.c., subcutaneous.

We developed two schemes for drug administration, both designed to rapidly deliver NLX to the bloodstream. The intravenous version (Fig. 2A) includes a printed circuit board (PCB) with interdigitated gold electrodes, drug and electrolyte reservoirs separated by an elastomeric membrane, in an enclosure formed in a biocompatible polymer with a catheter connection. Upon activation, electrolytic water splitting generates H_2_ and O_2_ gas in the electrolyte chamber. The resulting pressure deforms the membrane (polystyrene-*b*-polyisoprene-*b*-polystyrene; *E* = 4.3 MPa) and forces NLX from the drug reservoir into the vasculature via the intravenous catheter (Fig. 2B). Parametric modeling of the mechanics of this deformation process, supported by benchtop experimental studies, guides the selection of optimized design parameters (figs. S9 and S10), including a membrane diameter of 24 mm and thickness of 70 μm. The device accommodates a drug volume of 3 ml, capable of complete release of NLX directly into the bloodstream in less than 6 min (Fig. 2C and movie S1). The dead volume of the catheter (0.2 ml in Fig. 2C) accounts for a portion of the drug payload that is not infused. The doses quoted in the following correspond to the total volume loaded in the device. Accelerated degradation studies of the electrodes show adequate corrosion resistance to ensure stable operation for implanted durations exceeding 1 year (fig. S11).

The injector version of the Naloximeter (Fig. 2D) includes an actuating plunger, a compressible drug reservoir made of ethylene vinyl alcohol (EVOH) and a Huber needle. A small-scale DC motor drives the injection of NLX into the adjacent subcutaneous tissue, puncturing any fibrotic capsule that may form around the device as a foreign body response (*45*). A polytetrafluoroethylene/silicone septum seals the chamber at the needle outlet to prevent flow of biofluid into the enclosure, formed in a biocompatible polymer identical to the intravenous version (Fig. 2D, inset). The force required to deploy and retract the needle through the sealing septum is less than 1.3 N, far below the 10-N force delivered by the motor (figs. S12 and S13). The capacity of the drug reservoir is 1.5 ml. Complete delivery occurs within 25 s as a single dose, with a loss volume of <0.1 ml (Fig. 2E). The device can also be operated in dual-injection mode, with two 0.75-ml payloads delivered sequentially, as in Fig. 2 (E and F) and movie S2. The potency of fentanyl and its analogs often requires multiple NLX administrations to revive during an overdose event, thus motivating the dual-injection capability of the device (*22*, *23*). Results of additional characterization studies are in fig. S14. Further details on the device architecture and fabrication appear in Materials and Methods and figs. S15 and S16.

The electronic hardware and firmware are similar for these two device embodiments; the optical sensor is the same (Fig. 2A, inset, and fig. S2). Hypoxia studies in rodent and porcine models with stepwise modulation of inspired oxygen serve as the basis for validating measurements of StO_2_. In rodents, the calculated StO_2_ values correlate with SpO_2_ determined by a gold standard device (GSD), for SpO_2_ in the range of 60 to 100% across multiple animals (*N* = 3; Fig. 2, G and H, and fig. S17). Furthermore, comparisons of StO_2_ measurements against oxygen saturation (SO_2_) determined by blood gas analysis in hypoxic porcine models produce similar results (*N* = 3; Fig. 2I and figs. S18 and S19). Pharmacokinetic (PK) data for NLX delivered with the intravenous device in porcine models indicate swift delivery into the bloodstream. NLX appears in the plasma within 1 min of pump activation, and the peak concentration of 138 ± 10 ng/ml occurs at 2 ± 1 min after activation (*N* = 3; Fig. 2J). The variations can be attributed to differences in catheter length, diameter, and blood circulation between animals. The intravenous device includes a catheter with a normally closed slit valve, which is secured in the vasculature during implantation (Fig. 2J, inset, and figs. S20 and S21). Additional experiments used a gadolinium (Gd) tracer element found in MRI contrast agents as a proxy for NLX pharmacokinetics (Supplementary Materials and fig. S22). The injector device delivers NLX to the bloodstream with the typical kinetics of a subcutaneous or intramuscular injection (fig. S39). Specifically, the needle enters the tissue surrounding the device; the drug leaves the needle and diffuses from the injection point to nearby capillary vessels in the muscular and cutaneous tissue, where it enters the bloodstream (fig. S21D).

### Characterization of opioid overdose in large animal models

**Fig. 3.**
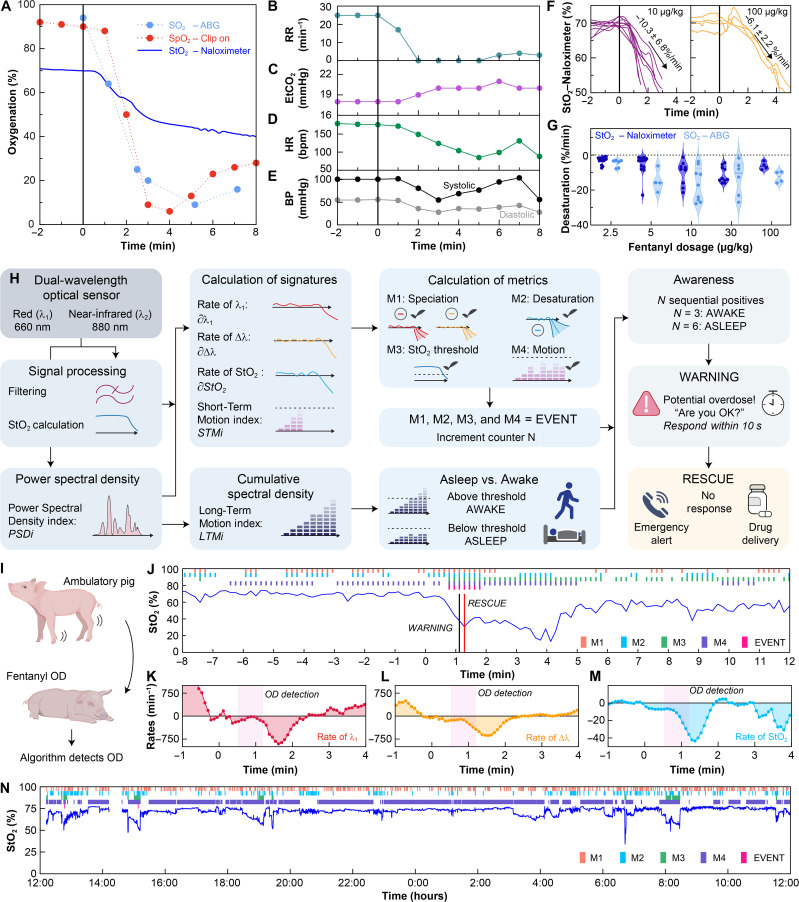
Physiological signs of opioid overdose and an algorithm for overdose detection in freely moving subjects. (**A**) StO_2_ recorded with a Naloximeter during a fentanyl overdose (OD; 10 μg/kg) in an anesthetized pig and its comparison with gold standards: SO_2_ (ABG) and SpO_2_ (pulse oximeter). (**B**) Decline of respiration rate, (**C**) elevation of end-tidal carbon dioxide (EtCO_2_), (**D**) bradycardia, and (**E**) reduction in blood pressure after fentanyl injection (at time = 0) represent clinical signs of overdose. (**F**) StO_2_ recorded from Naloximeters during fentanyl overdose quantify the rate of desaturation. Left, *n* = 9 devices and right, *n* = 4 devices. (**G**) Desaturation rates following various dosages of fentanyl. *N* = 29 subjects and *n* = 52 devices; dose-specific sample sizes are included in table S2. (**H**) Illustration of the analytical approach for the ODA based on the dual-wavelength optical sensor. Definition of metrics: optical absorption and its rate of change (M1), desaturation rate (M2), tissue oxygenation levels (M3), and signal power density (M4). (**I**) Operation of the ODA in an ambulatory pig, including (**J**) StO_2_ during opioid overdose (30 μg/kg), and the rates of change of (**K**) the red optical signal (*∂*λ_1_), (**L**) the difference between the red and infrared optical signals (*∂*∆λ) of the dual-wavelength optical sensor, and (**M**) the tissue oxygenation (*∂StO*_2_). Fentanyl was administered at time = 0. (**N**) Application of the ODA in a healthy, normally ambulating pig for 24 hours. Although several instances of synchronous positive metrics, i.e., EVENTs were registered, none of them led to a WARNING. Colored bars in (J) and (N) indicate time stamps where individual metrics (M1, M2, M3, and M4) or compound logical metrics (EVENT) are true.

The physiological manifestation of opioid overdose consists of the gradual reduction, including possible cessation, of oxygen uptake in the lungs (Fig. 3A), driven by respiratory depression (Fig. 3B) and accompanied by an increment in end-tidal carbon dioxide (EtCO_2_, Fig. 3C). Respiratory depression often precipitates cardiac depression, observed as bradycardia (Fig. 3D) and low blood pressure (Fig. 3E). The circulation of excess deoxygenated blood produces systemic oxygen deprivation, as observed with clinical measurements (arterial blood gas: ABG) and optical sensors (Naloximeter: StO_2_, standard pulse oximeter: SpO_2_) in Fig. 3A. This oxygen desaturation occurs on the timescale of several seconds to 1 min after fentanyl administration. The minimum dosage of fentanyl that will produce fatal overdose in humans is difficult to predict because the respiratory response depends on the health and opioid tolerance of the individual subject. After fatal overdose, often the only data available are the fentanyl concentrations in plasma, not the dosage that precipitated the overdose (*46*). Nevertheless, the Drug Enforcement Administration estimates a lethal dose of approximately 30 μg/kg (2 mg) in humans and recommends ventilatory support for doses above 10 μg/kg (*47*, *48*). Experiments at five dosages of intravenous fentanyl in the range of 2.5 to 100 μg/kg in porcine models indicates that the oxygenation response is similar for doses above 10 μg/kg, producing a desaturation event with oxygen decline at a rate of ~−10.4% StO_2_ per minute (Fig. 3F), as confirmed with ABG analysis (~−13.9% SO_2_ per minute, Fig. 3G). The observed desaturation rates produced by these dosages yield severe hypoxia (<70% SO_2_) within 3 min, with risk of global ischemia and brain injury if sustained over several minutes (*49*). Smaller fentanyl doses lead to reduced desaturation rates (Fig. 3G and fig. S23) but equally fatal outcomes. Substantial variability in the desaturation rate for dosages above 10 μg/kg reflects the diversity of responses to fentanyl between individuals and even within the same animal. As with humans, the response to fentanyl is highly dependent on factors such as age, genetics, body weight, and concomitant medications (*50*–*52*).

Detecting an overdose with high specificity and selectivity requires accurate interpretation of physiological signatures while discriminating against false positives induced by motions and other artifacts. At the onset of OIRD, the transition to hypoxia produces changes in tissue optical absorption due to the speciation of deoxy- and oxygenated hemoglobin (*53*). An increase in deoxygenated hemoglobin produces an increase in optical absorption at the red wavelength and a corresponding reduction in the red optical signal at the sensor, with an opposite effect for the near-infrared signal (fig. S16 and S24). These changes differ from motion-induced artifacts, where rapid fluctuations in both signals produce short-lived variations in the calculated values of StO_2_. Power spectral density analysis of data from humans performing specific movements shows that the reduction of spectral content in the 1- to 5-Hz range correlates with the reduction of gross body motions, thus serving as an indicator of the intoxicated overdose state (fig. S25 and S26). The Overdose Detection Algorithm (ODA) uses multivariable analysis of data from the optical sensor to operate reliably and quickly (within 90 s of fentanyl administration) to detect overdose and reject false positives during normal ambulation, as illustrated in Fig. 3H. The ODA calculates the StO_2_ and an index of motion (*STMi*, cumulative over 60 s), and it determines the rates of change of the StO_2_ (*∂StO*_2_), the magnitude of the red signal (*∂*λ_1_), and the difference between the red and infrared signals (∂Δλ). Comparisons of these parameters against threshold values produce four logical metrics every 10 s. The mutually inclusive condition of all four metrics suggests a potential overdose episode (an “EVENT”). A second motion index (*LTMi*, cumulative over 10 min) determines whether the subject is awake or asleep (fig. S27). An uninterrupted successive series of EVENTs detected within 30 s leads to a warning flag (a “WARNING”) that produces a push notification on the mobile app to prompt user engagement. When the subject is asleep, the ODA adjusts the triggering time to 60 s to account for confounding desaturation produced during obstructive sleep apnea. If the user fails to acknowledge this notification within 10 s, the system launches the closed-loop rescue cascade: activating the drug delivery mechanism and relaying an emergency alert. Further details regarding the algorithm are in Supplementary text.

Figure 3I depicts tests of the ODA, performed through multiple fentanyl overdoses with ambulatory porcine models. A dose of fentanyl (30 μg/kg) leads to cyanosis, respiratory depression, stiff posture, and muscle tremors in these animals. In one animal, the ODA detects the early signs of overdose (first EVENT) within 37 s after fentanyl administration, as the onset of a steady decrease in StO_2_ (Fig. 3J) accompanied by sequential positive metric conditions that confirm the overdose (Fig. 3, K to M, and fig. S28). A WARNING at 67 s precedes the rescue cascade at 77 s (Fig. 3J). A 24-hour period of continuous monitoring in a freely moving pig serves as the basis for further validation of the ODA (Fig. 3N; with additional datasets, *N* = 3 total, in fig. S29). This example shows multiple episodes where the StO_2_ approaches clinically dangerous levels, but the absence of sequential occurrences of positive metrics (fig. S30) avoids false indications of an overdose. Further, we did not observe any false negatives (failure to detect overdose) in datasets where an overdose occurred.

### Device rescues freely moving animals from opioid overdose

**Fig. 4.**
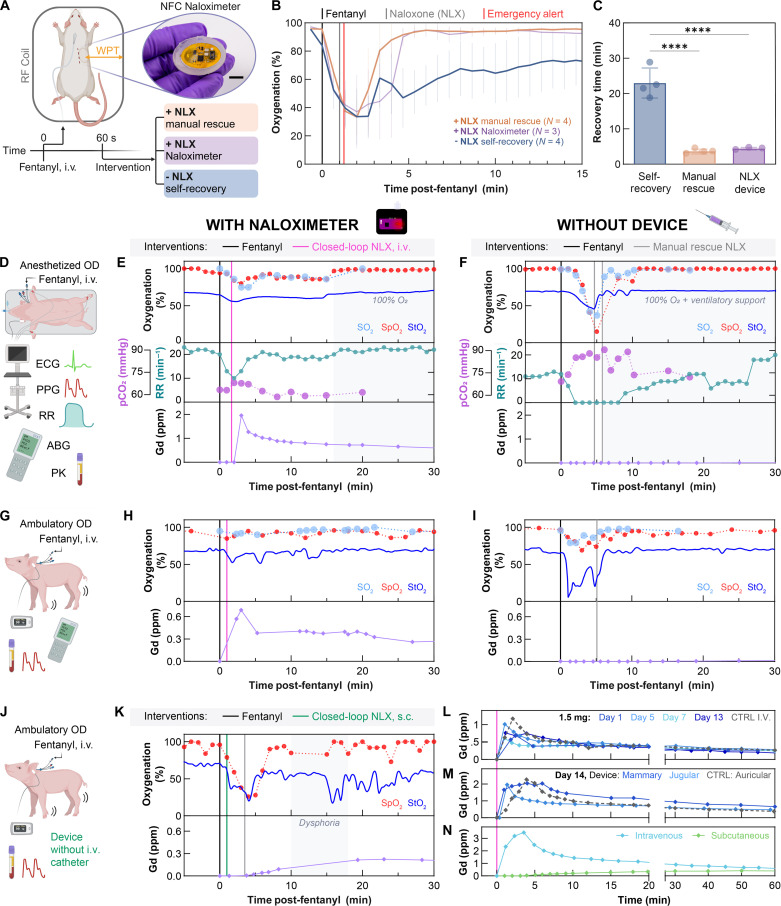
Demonstration of autonomous rescue from life-threatening opioid overdose in rodents and freely moving porcine models. (**A**) Subcutaneous implant location for a miniaturized NFC Naloximeter and corresponding fentanyl overdose experiment grouping by NLX treatments; inset: optical image of the device; scale bar, 1 cm. (**B**) Oxygenation in a rodent model following fentanyl overdose and manual subcutaneous administration of NLX (manual rescue), autonomous rescue enabled by the NFC Naloximeter, or self-recovery without NLX. Sample sizes are in the legend. (**C**) Comparison of recovery time between treatment groups (one-way ANOVA, ****P < 0.0001). Data points correspond to individual animals, and bars depict the means. (**D**) Experimental setup for overdose (OD) rescue in an anesthetized pig model (**E**) with and (**F**) without a Naloximeter, the latter required manually administered NLX to rescue. Oxygenation (top), respiratory vitals (middle), and pharmacokinetic data (bottom) for each fentanyl overdose. (**G**) Experimental setup for OD rescue in ambulatory pigs (**H**) with and (**I**) without a Naloximeter. (**J**) Demonstration of subcutaneous drug delivery from (**K**) a Naloximeter without intravenous catheter in an ambulatory pig, insufficient to recover from OD, requiring manually administered NLX to rescue. Oxygenation (top) and PK (bottom) data for each fentanyl overdose. The oxygenation in (E), (F), (H), (I), and (K) was measured with blood gases (SO_2_), a commercial pulse oximeter (SpO_2_), and the Naloximeter (StO_2_). (**L**) Longitudinal PK data from an intravenous Naloximeter at 1 to 13 days after implantation, and an infusion pump as a control (CTRL I.V.). Dose is 1.5 ml. (**M**) Gd pharmacokinetics from a Naloximeter (device) at 14 days after implantation in the jugular or mammary vein, or infusion pump (CTRL) in the auricular vein. Dose is 2 ml. (**N**) Gd PK profile when delivered by a Naloximeter intravenously (jugular vein) or subcutaneously. Dose is 3 ml. (A), (D), (G), and (J) created with BioRender.com.

Experiments in rodent models validate the closed-loop rescue concept with a miniaturized, battery-free version of the electrolytic Naloximeter designed for research purposes, implemented without the catheter (Fig. 4A, inset). This device operates via NFC protocols and wireless power transfer, as described in Materials and Methods and figs. S31 and S32. As with pigs, administration of a sufficient dose of fentanyl in rats leads to a rapid desaturation event. The NFC Naloximeter detects signs of an overdose within 1 min and then triggers a rescue cascade as described previously, with an emergency alert received at 70 s after fentanyl administration. The animals show signs of recovery within 4 min and full recovery (90% SpO_2_) after 4.5 min (*N* = 3; Fig. 4B). Comparisons of overdose recovery between groups rescued by the Naloximeter, manual subcutaneous NLX injection, or without administration of NLX (self-recovery) highlight the rescue capabilities of the device (Fig. 4C). Overdose recovery time is highly significant (*P* < 0.0001) between the Naloximeter and self-recovery (*N* = 3 and 4; recovery times = 4.5 ± 0.3 and 23 ± 4 min, respectively). The same is true between manual rescue (*N* = 4; recovery time = 3.7 ± 0.6 min) and self-recovery (*P* < 0.0001).

Large animal rescue is more complex because, as with humans, the response to fentanyl varies between individuals. Survival studies in pigs simulate real-world uses of the intravenous Naloximeter for humans. Details of the surgical approach, experiment timeline, and device biointegration are in Materials and Methods and figs. S20 and S33 to S35. Initial experiments validate the closed-loop rescue capability using animals anesthetized with the hypnotic agent propofol and monitored using clinical systems (Fig. 4D). In one example, the pig was administered a dose of fentanyl (2.5 μg/kg) after a postoperative healing period of 14 days. The onset of respiratory depression occurred within 1 min, causing oxygen desaturation and hypercapnia observed by the optical sensor in the Naloximeter (StO_2_) and clinical systems (SO_2_ and SpO_2_) between 1 and 1.5 min (Fig. 4E, top and middle). The Naloximeter triggered closed-loop drug delivery at 1.67 min after fentanyl administration, and Gd (NLX proxy) appeared in the bloodstream by 3 min (Fig. 4E, bottom). The animal recovered to baseline respiratory rate within 5 min of closed-loop rescue, and oxygenation remained unchanged until the inspired oxygen returns to 100% at 15 min after administration. A control experiment involved delivery of the same dose to the same animal without activation of the Naloximeter. As before, respiratory depression onset occurred at 1 min, but without the Naloximeter rescue, apnea occurred at 2 min, with notable hypoxia and hypercapnia (Fig. 4F) until the manual administration of two doses of NLX and rescue breaths. Delivery of excess inspired oxygen (100%) and mechanical ventilation supported the animal to recovery. Additional Naloximeter rescue demonstrations (*N* = 4 total) and further vital signs are in figs. S36 to S38.

Tests of fentanyl overdose in conscious, freely moving pigs validated the ODA and rescue capabilities of the Naloximeter for eventual applications with humans (Fig. 4G). In this case, the ODA detected the overdose at 55 s after fentanyl administration (30 μg/kg) and triggered the rescue cascade at 65 s. The initial desaturation event was short-lived because of the reversal effect of NLX, and the pig recovered to baseline levels of oxygenation within 10 min of the overdose (Fig. 4H, top). PK data show the NLX proxy in the bloodstream within 1 min of pump activation (Fig. 4H, bottom). The control case led to a severe desaturation event with signs of possible reoxygenation at 3 min followed by an immediate sharp desaturation before imminent death averted by manual rescue (Fig. 4I). An important additional observation is that subcutaneous drug delivery with the same electrolytic pumping Naloximeter performed without the catheter (Fig. 4J) is insufficient to rescue from fentanyl overdose (30 μg/kg), as illustrated by a deep desaturation event (Fig. 4K, top) that is similar to the control case. Specifically, despite closed-loop triggering of the rescue cascade at 1 min, PK data show very low levels of drug in the bloodstream even after 5 min in this delivery modality (Fig. 4K, bottom). These findings likely result from frustrated diffusion of NLX through fibrotic tissue that forms around the implant, illustrated in fig. S21E. Intravenous Naloximeters with catheters implanted for several weeks in both peripheral and central vasculature show similar PK profiles to the control case (infusion pump; Fig. 4, L and M). When compared directly to subcutaneous drug delivery without the catheter (Fig. 4N), the rapid delivery of NLX to the bloodstream is clearly critical to overdose rescue. Additional PK studies (*N* = 11) in fig. S39 substantiate this trend.

## DISCUSSION

This work introduces three Naloximeter device platforms for autonomous rescue from opioid overdose: one that enables fundamental studies in rodent models and two options suitable for clinical translation to humans. These three devices share the same fundamental platform (optical sensor and drug delivery device) but differ in their drug delivery mechanism and application. Survival experiments in large and small animals demonstrate the capabilities for automatically detecting and quickly responding to overdose through the combined use of a dual-wavelength optical sensor and a drug delivery system. In general, we observe that overdoses treated with the Naloximeter are much less severe, with minimal lasting effects to overall health. Rapid delivery of NLX to the bloodstream is key to rescue, a design feature in both Naloximeter embodiments for clinical translation.

The envisioned patient population includes those in recovery from OUD due to their high risk of fatal overdose, where device implantation can occur just before their release from treatment. Knock-on effects of nonfatal overdose are a part of OUD that are often overlooked but contribute considerably to the morbidity observed in those who have survived an overdose (*16*, *17*). For implantation in humans, we envision outpatient procedures that align with traditional subcutaneous pacemakers and vascular access ports, surgeries encountered by millions worldwide (*54*–*57*). While there is a risk of thrombus and complications with indwelling catheters, we observed no significant difference in a biomarker for cardiac damage between samples drawn pre- and post-Naloximeter activation (*N* = 12; fig. S40). We attribute this to the catheter locking solution and the normally closed Groshong valve (*58*), which prevents blood backflow while still allowing for infusion, as described further in Supplementary text. The injector Naloximeter platform was conceived to address concerns regarding long-term catheter patency. Performing drug administration via injection rather than intravenous delivery isolates the device from the bloodstream and removes the clotting risk, at the cost of speed of NLX to the bloodstream, which is optimized in the intravenous embodiment. Further in vivo studies to assess the longitudinal catheter patency at much longer timescales would illuminate the feasibility of a catheter-based drug delivery mode. The availability of both technologies provides valuable options for the patient and their physician. Future work will include detailed comparative studies, with specific focus on the injector version.

The simplicity of the optical sensor and associated algorithms and their ability to function reliably in porcine models represent attractive aspects of the engineering approaches described here. Further study of overdose detection and false positive rejection in humans would be essential for clinical translation. In particular, events that may cause respiratory depression or apnea other than overdose, e.g., traumatic injury, require careful consideration and likely additional sensors for motion or heart/respiration rate to enable discrimination of these events. A demonstration of multimodal sensing capability with oxygenation and accelerometry is included in fig. S26. Of course, additional sensors may enhance the robustness of the device at the expense of engineering complexity. Another consideration for translation is the battery life, which lasts approximately 1 week (6.9 days) before requiring recharge, a feature that was demonstrated in porcine models (fig. S6). Wireless recharging is an accepted technology for implanted devices such as nerve stimulators or pain management drug delivery systems. Battery life could be extended to 11 weeks by increasing battery capacity while maintaining the same lateral footprint of the Naloximeter device, as described in Supplementary text.

Future work may consider translation of the Naloximeter platform to a wearable device; however, drawbacks including patient compliance and potential abuse of an easily replaceable platform are key ethical issues to consider. In addition, the translatable technology would require an on-device data analytics system as a safety mechanism in case BLE communication with the cellular device fails, thus maintaining continuous data processing for overdose detection. Although demonstrated specifically in the context of opioid overdose, the Naloximeter platform is not limited to this disease paradigm and can be adapted to treat other emergency rescue scenarios with rapid PK demands such as anaphylaxis or epilepsy.

## MATERIALS AND METHODS

### Description of the electronics module

The electronics consisted of two PCBs (1 oz. Cu on FR-4, 0.4 mm), one active and one passive. The controlling PCB (active), 15 mm by 31 mm, contained all electronic components, while a supporting PCB (passive), 34 mm by 42 mm for the intravenous device and 31 mm by 45 mm for the injector device, contained the planar coil for wireless recharging and interdigitated electrodes for electrolytic gas generation (intravenous device). Electroless nickel immersion gold (ENIG) plating formed a conformal gold layer (75 nm) on the Cu interdigitated electrodes. The controlling electronics comprised a low power Arm Cortex M3 microcontroller unit (MCU, CC2640R2F, 5 mm by 5 mm, Texas Instruments) that supports BLE 5.1 communication protocols. This MCU controlled all aspects of device operation and wireless communication. An integrated optical sensor (MAX30101, Maxim Integrated Products Inc.) acted as a dual-wavelength optical sensor equipped with light-emitting diodes (LEDs: red, 660 nm and near-infrared, 880 nm). This component interfaced with the MCU for the recording of photocurrent from the embedded silicon photodetector (PD) at 50 samples/s and 16-bit resolution. Two general purpose input/output pins in the MCU served as logical controls for actuation of drug delivery. In the intravenous device configuration, an n-type metal oxide semiconductor field-effect transistor served as a switch for electrolytic gas generation. In the injector configuration, an H-bridge driver chip (DRV8837C, Texas Instruments) provided power to the DC motor in both forward and backward directions. Magnetic induction was used to wirelessly charge a 75-mAh LiPo battery. During wireless charging, a transmitter antenna operating at the industrial, scientific, and medical band of 13.56 MHz, coupled with the planar coil on the supporting PCB, enabled wireless power transfer. The induced alternating voltage was rectified and regulated to 5 V to power the battery management chip (LTC4065EDC, Analog Devices Inc.) at a charging current of ~30 mA. The system design also included a remote reset mechanism for the MCU in the event of a failure in the process of starting the device. A negative temperature coefficient (NTC) sensor interfaced with the MCU to provide measurements of temperature. Figures S1 and S2 show an electronic circuit diagram and functional block diagram of the device, respectively. Heat generated by the optical sensor and device electronics during operation is negligible, as measured by an infrared camera (FLIR A655sc, Teledyne FLIR) shown in fig. S5. Wireless recharging of devices generated minimal thermal load, as measured by the NTC on the device and plotted in fig. S6 (B and C) for the implanted and nonimplanted cases, respectively. The temperature of devices during charging (*T*_max_ = 41.7°C) is below the threshold for burn injury, which can be as high as 43°C for up to 8 hours (*59*).

### Fabrication of Naloximeters for porcine model studies

The fabrication of the implantable Naloximeter devices consisted of three parts: electronics module, drug delivery pump (two types), and encapsulation.

#### Electronics module

The designs of the PCBs made use of a commercial computer-aided design utility (Autodesk Fusion 360, Autodesk Inc.) and an outsourcing vendor (PCBWay Inc.) for fabrication. Low-temperature soldering bonded the electronic components to the PCBs. Half-cut castellated holes served as a means for mounting the controlling PCB to the supporting PCB. After connecting a battery to the supporting PCB, marine epoxy (Marine Epoxy, Loctite) covered the exposed soldering connections.

#### Drug delivery pump–Intravenous

Pump assembly began with the fabrication of flexible membranes. The block copolymer polystyrene-*b*-polyisoprene-*b-*polystyrene at 22 wt % styrene (SIS22; Sigma-Aldrich) was dispersed in toluene (≥99.5% purity, Sigma-Aldrich) at 30 wt % concentration using a combination of ultrasonication and stirring at 40°C. Spin-coating this solution on a silicon wafer treated with a hydrophobic coating (Rain-X Inc.) formed the membrane film: thickness, ~70 μm. Membranes remained at room temperature in a fume hood for at least 1 hour following spin-coating to ensure the evaporation of the volatile solvent.

Figure S14 illustrates the methods for assembling the intravenous pump. Briefly, a stereolithographic (SLA) three-dimensional (3D) printer (Form 3B, Formlabs Inc.) fabricated the device housing (drug reservoir) and electrolyte reservoir in a biocompatible resin (BioMed Clear V1, Formlabs). After the removal of printing supports, sanding and sonication produced finished parts with low surface roughness. Waterproof polyurethane sealant (3M Marine Adhesive Sealant Fast-Cure 5200, 3M Inc.) bonded the electrolyte reservoir to the membrane. After curing, the membrane/electrolyte reservoir was released from the substrate and inserted into the device housing with the same sealant to form the drug reservoir with a water-tight bond. A male Luer lock fitting (716281, QOSINA Corp.) was secured within the device housing with superglue (Gel Control super glue, Loctite) and additional waterproof sealant. After inserting the battery into the device chamber, the supporting PCB containing the electrode bonded to the electrolyte reservoir and device housing with additional waterproof sealant.

#### Drug delivery pump–Injector

Fabrication of pumps for the injector device began with SLA 3D printing of a two-piece housing, plungers and flexible bridges in the same biocompatible resin described above. The drug reservoir consisted of 50-μm-thick EVOH film (Non-Catch, Kyodoprinting) manually cut and heat-sealed to the desired shapes. Bonding the EVOH drug reservoir and Huber needle (22G, SAI Infusion) with waterproof polyurethane sealant and marine epoxy completed the fabrication of the needle plunger structure. Biomedical epoxy (EA M-31CL, Loctite) secured the plungers, a 316 stainless steel nut, and four flexible bridges together to form a plunger subassembly. After inserting the battery, motor, and the plunger subassembly into the lower half of the device housing, waterproof polyurethane sealant bonded the supporting PCB to the lower portion of the device housing. Additional waterproof sealant secured the upper device housing with the lower portion to complete the pump assembly for the injector device.

#### Encapsulation

Figure S15 illustrates the encapsulation process, beginning with the controlling PCB in the electronics module. A liquid-phase adhesion-promoter treatment used 3-(trimethoxysilyl)propyl methacrylate (MilliporeSigma) in a 1:50:50 volumetric mixture with isopropanol and deionized water and was applied immediately before depositing a conformal coating of parylene-C (10 to 13 μm, dichloro [2,2] paracyclophane, SCS Labcoater II, Specialty Coating Systems Inc.). Thereafter, the controlling PCB was soldered to the supporting PCB and assembled pump. Biomedical epoxy then covered both PCBs as a secondary biofluid barrier. A molded encapsulation layer of poly(dimethylsiloxane) (PDMS; Sylgard 184, Dow Corning) provided a soft and biocompatible tissue interface. In the intravenous device, a filling port (0.6-mm diameter) was drilled at the electrolyte reservoir level after encapsulation. An aqueous solution of potassium hydroxide (50 mM, Fisher Chemical) served as the electrolyte, and the filling port was sealed with fast-curing silicone adhesive (Kwik-Sil, World Precision Instruments). In the case of the injector device, tape sealed an outlet hole for the needle before forming the PDMS encapsulation. Once cured, a biopsy punch removed the tape and PDMS at the outlet to expose the interior of the chamber. A polytetrafluoroethylene (PTFE)/silicone sealing septum (Sigma-Aldrich), secured with Kwik-Sil, covered the opened outlet. The dimensions of the intravenous and injector devices were 46 mm by 36 mm by 16.5 mm and 55 mm by 46.5 mm by 18.5 mm, respectively.

### Fabrication of miniaturized Naloximeters for rodent model studies

Fabrication of the battery-free electrolytic pumps designed for use in rodents followed the same procedures as described above but on a smaller scale. Two-sided flexible PCBs (f-PCBs, PCBWay Inc) supported interdigitated gold-plated electrodes and electronic components on opposite sides. Low-temperature solder bonded surface-mount components to the bottom side of the f-PCB. The top side (containing electrodes) was covered with a laser-cut release liner to protect the active metal surface during the deposition of parylene-C (10 to 13 μm) on the f-PCB, as described above. After removing the release liner, the f-PCB was attached to the electrolyte reservoirs using a layer of waterproof polyurethane sealant. The devices were encapsulated in a similar molding process as above, with a layer of PDMS over the electronics, and an overcoat of a soft, biocompatible silicone (Ecoflex 00-30, Smooth-On Inc.) to form the upper aspect of the encapsulation. The drug outlet at the top of the device was sealed before encapsulation and reopened after demolding, at which point a microfluidic channel layer (Ecoflex 00-30) was secured at the outlet with fast-curing silicone (Ecoflex 00-35 FAST, Smooth-On Inc.). The circuit and logic diagrams and exploded view of the device are shown in figs. S31 and S32.

### Studies of release rate from the intravenous device

A 10 mM aqueous solution of Rhodamine B (Acros Organics) filled the drug reservoir of the electrolytic pump. Deionized water (70 to 90 g) was added to a beaker with a magnetic stir bar and placed onto a stirring plate. A Groshong catheter (7 French) was filled with water from the beaker and connected to the device, with the free end of the catheter secured in the beaker of water. The electrolytic pump was activated via command from the mobile application, and aliquots from the cup were collected every 30 to 60 s for 10 min. A benchtop plate reader (Synergy NEO2, BioTek Instruments Inc.) analyzed the aliquots in duplicate by measuring the absorbance spectrum between 450 and 650 nm. A calibration curve with reference concentration samples was included in each plate. The absorbance value at 553 nm was used to calculate the time-resolved dye concentration in the beaker (accounting for volume loss due to aliquot sampling) based on the Beer-Lambert Law. The volume of dye released by the pump was then computed on the basis of a simple dilution factor.

### Calculation of tissue oxygenation (StO_2_)

A differential spectroscopy method estimated the regional tissue oxygenation (StO_2_) using the two LED colors (λ_1_ = 660 nm and λ_2_ = 880 nm) and the integrated silicon PD of the biometric optical sensor (MAX30101, Maxim Integrated). This method used the modified Beer-Lambert law of optical absorption in scattering media (*60*–*62*) to calculate changes in oxygenated (HbO_2_) and deoxygenated (Hb) hemoglobin. At λ_1_ = 660 nm, the molar absorption coefficients are ε_Hb-λ1_ = 344.2 mm^−1^ M^−1^ and ε_HbO2-λ1_ = 44.5 mm^−1^ M^−1^, whereas for λ_2_ = 880 nm, they are ε_Hb-λ2_ = 83.57 mm^−1^ M^−1^ and ε_HbO2-λ2_ = 119.9 mm^−1^ M^−1^ (*53*). Solving the linear equation, Eq. 1, resulting from the differential optical losses at the two wavelengths yielded the changes in the concentration of Hb (∆*cHb*) and HbO_2_ (∆*cHbO*_2_) with respect to baseline

$$\left[\begin{array}{c} ∆\mathrm{cHb}\\ ∆{\text{cHbO}}_{2} \end{array}\right]=\frac{1}{d\;v_{b}}{\left[\begin{array}{cc} {\mathrm{DPL}}_{\lambda 1}\;\varepsilon_{\mathrm{Hb}-\lambda 1} & {\mathrm{DPL}}_{\lambda 1}\;\varepsilon_{\mathrm{HbO}2-\lambda 1}\\ {\mathrm{DPL}}_{\lambda 2}\;\varepsilon_{\mathrm{Hb}-\lambda 2} & {\mathrm{DPL}}_{\lambda 2}\;\varepsilon_{\mathrm{HbO}2-\lambda 2} \end{array}\right]}^{-1}\left[\begin{array}{c} ∆A_{\lambda 1}\\ ∆A_{\lambda 2} \end{array}\right]$$ (1)

Here, *d* is the LED-PD separation distance (3.25 mm), *v_b_* is the blood volume fraction of the tissue, *DPL*_λ1,2_ are the differential path lengths at the two wavelengths (λ_1_ and λ_2_). The time-dependent difference in the optical signal (∆*A*_λ1,2_) with respect to baseline, measured at the two wavelengths, is ∆*A*_λ1,2_(*t*) =−log_10_[*I*_λ1,2_(*t*)/*I*_λ1,2_(*t*_0_)], where *I*_λ1,2_(*t*) is the digitalized photocurrent data at time *t* and *I*_λ1,2_(*t*_0_) is the photocurrent recorded at baseline (*t*_0_). The ratio of oxygenated hemoglobin (*cHbO*_2_) to total hemoglobin (*cHbO*_2_ + *cHb*) on a concentration basis defines the tissue oxygenation *StO*_2_(*t*), Eq. 2

$${\mathrm{StO}}_{2}\left(t\right)=\frac{{\text{cHbO}}_{2}\left(t\right)}{{\text{cHbO}}_{2}\left(t\right)+\mathrm{cHb}\left(t\right)}$$ (2)

where *cHbO*_2_(*t*) = *cHbO*_2_(*t*_0_) + ∆*cHbO*_2_(*t*), and *cHb*(*t*) = *cHb*(*t*_0_) + ∆*cHb*(*t*). In porcine muscular tissue, *v_b_* = 1.0%, *DPL*_λ1_ = 9.65 and *DPL*_λ2_ = 9.09 (*63*, *64*). Devices placed in different locations of the pig (fig. S18) show similar results versus an SO_2_ gold standard, but desaturation rate and depth varied slightly during hypoxia, likely a result of tissue-dependent variations in ν*_b_* (*64*). The baseline *StO*_2_ is defined as 70%, and the concentration of total hemoglobin is 130 g/liter (2 mM), which corresponds to baseline *cHb*(*t*_0_) = 0.6 mM and *cHbO*_2_(*t*_0_) = 1.4 mM. The slope of a first-order linear interpolation of the tissue oxygenation data over a window of 1 to 3 min of steady signal decrement post-fentanyl administration produced the desaturation rate metric reported in Fig. 3G.

### Large animal studies

All large animal studies were performed according to the protocol approved by the Northwestern University Institutional Animal Care and Use Committee (study #00018098). Female Yorkshire Landrace crossbred pigs (27 to 30 kg) were obtained from Oak Hill Genetics (Ewing, IL, USA) and examined by veterinary staff upon arrival. The pigs were housed in groups of two to three animals on a 14:10-hour light:dark (LD) cycle (lights on at 6:00 a.m.) during an acclimation period of at least 5 days before any surgical intervention. Following device implantation surgery, the pigs were single-housed and remained on the same 12:12 LD cycle. The temperature in the facility ranged from 21° to 24°C, while the humidity was maintained between 30 and 70%. The pigs were fed a standard diet of pig feed twice daily throughout the experiments, which were conducted during the light cycle. The pigs were fasted for 12 hours preceding any surgery or experimental procedures, and water was available ad libitum. Figure S30 shows the general experiment timeline.

#### Device implantation procedure

Ethylene oxide gas sterilized the closed-loop devices in a standard 24-hour cycle (Anprolene AN74, Andersen Sterilizers). Using aseptic technique, the devices were filled with drug solution containing a 9:1 volumetric mixture of NLX (NLX HCl injection, 1 mg/ml; International Medication Systems) and Gd contrast agent (ProHance, gadoteridol injection, 279.3 mg/ml; Bracco Diagnostics Inc.) immediately before implantation. A sterile one-way duckbill valve (11582, QOSINA Corp.) was attached to the device once filled.

Pigs were sedated and prepared for surgery with telazol (6 mg/kg), propofol (0.5 to 1.5 mg/kg), and atropine (0.05 mg/kg) and intubated. Isoflurane anesthetic (1 to 5%) was used to effect throughout all surgical procedures. Carprofen (4 mg/kg) and bupivacaine (4 to 10 ml) served as preoperative and local analgesics, respectively. Antibiotic cefazolin (22 mg/kg) and anticoagulant heparin (100 mg/kg) were administered intraoperatively. Figure S20 illustrates the implantation procedure for devices in the jugular vein. Incision sites were shaved and scrubbed three times with alternating Betadine and ethanol swabs to disinfect. For devices in the jugular vein, a 3- to 5-cm incision was made in the ventral neck and blunt dissected to expose the external jugular vein. An 8- to 10-cm incision bisecting the brachiocephalic muscle was made along the dorsal aspect of the neck, and blunt dissection produced a subcutaneous pocket for the device. A trocar created a small subcutaneous tunnel between the device pocket and the vascular access incision, and the intravenous catheter (5 to 8 French) was pulled through. The catheter was measured and cut to a length that ensured 7 to 10 cm of the catheter in the vessel and minimal slack between the device and vascular access. A blunt needle or similar Luer connector supplied by the manufacturer connected the device to the free end of the catheter, which had been locked with a solution of dextrose/heparin (500 IU/ml of sodium heparin, 25 wt % dextrose) to maintain patency (*65*). At this point, the device was inserted into the subcutaneous pocket with the PCB side facing the muscular tissue, and the catheter protruded from the neck incision. The catheter was introduced to the vessel and secured with finger-trap sutures (3-0 silk). A suture (4-0 silk) through the fixation wing on the device secured it to the underlying muscular tissue. The subcutaneous space was closed with absorbable tacking sutures (3-0 PDS, polydioxanone). Last, the skin was closed in layers with absorbable sutures (3-0 PDS) in the subcutaneous layer and barbed absorbable sutures (2-0 Quill) in the dermis.

A similar method was used in the implantation of intravenous devices in the mammary vein, with an incision just above the nipple line for venous access and a subcutaneous device pocket along the upper aspect of the iliocostalis. Figures S31 and S32 show examples of healed devices implanted in the jugular and mammary veins, respectively.

The pigs were administered carprofen (2 mg/kg) for 2 days postoperatively and antibiotics (16 mg/kg, Clavamox), aspirin (325-mg tablet), and clopidogrel (75-mg tablet, Plavix) for the duration of the study. The pigs recovered for at least 2 days following device implantation before additional surgical procedures, i.e., catheterization. When necessary, the devices were recharged transdermally (fig. S6) with a custom wireless charger consisting of a transmitting coil (diameter 10 to 12 cm, two turns) connected to a radio frequency power supply (13.56 MHz, 10 W, Neurolux Inc.).

#### Catheterization procedure (porcine)

Preoperative medication, sedation, and aseptic procedures were identical to that used in device implantation surgery. A standard central venous catheterization approach was used, in which the pig is positioned in dorsal recumbency and a midline incision (2 to 4 cm) is made in the ventral neck. Blunt dissection and isolation of the carotid artery and jugular vein followed by insertion of catheters (8 to 9.5 French), secured with finger-trap sutures (3-0 silk) established vascular access. Subcutaneous tunneling with a trocar to the dorsal aspect of the neck created exit sites for both catheters. Catheter patency was confirmed, and the indwelling lines were locked with heparinized saline before closure. The midline incision was closed in layers with absorbable sutures (3-0 PDS) in the subcutaneous layer and barbed absorbable sutures (2-0 Quill) in the dermis. Additional finger-trap sutures secured the lines to the skin at their exit site, and a veterinary jacket (MPS-Top Shirt Dog, Medical Pet Shirts) worn by the animal minimized the risk of complications from the indwelling lines.

#### Fentanyl overdose experiments (porcine)

In anesthetized studies, the pigs were sedated with telazol (6 mg/kg) and atropine (0.05 mg/kg) and intubated. A propofol infusion of 6 to 12 mg kg^−1^ hour^−1^ maintained light anesthesia. Physiological monitoring devices were placed to monitor heart rate (electrocardiography), SpO_2_ and pulse (PPG), and respiratory vitals (capnography) with a veterinary vitals machine (LifeWindow 6000 V, Digicare Biomedical). A handheld blood gas analyzer (i-STAT 1, CG8+ cartridges, Abbott Laboratories) provided on-site measurements of blood gases (ABG) from fresh arterial samples. Samples for small-molecule PK analysis were collected in EDTA plasma tubes (K_2_EDTA Vacuette, Greiner Bio-One), stored on ice, and centrifuged within 1 hour of collection.

The pig was breathing spontaneously throughout the duration of the study, and the inhalational gas mixture was titrated from 100% oxygen to 100% medical air in the 30 min before fentanyl administration. Baseline data were recorded with the implanted devices and physiological monitoring devices. A baseline blood sample for ABG and PK was drawn within the 5 min preceding fentanyl administration (fentanyl citrate, intravenous, 2.5 to 10 μg/kg), which occurred at time = 0. Blood samples for PK were collected every 1 min for the first 10 min, every 2 to 3 min for the following 10 min, and every 10 min thereafter until time = 60 min. Blood samples for ABGs were collected at the same frequency as PK samples until recovery (approximately 20 min), limited by the running time of the instrument.

In ambulatory experiments, pigs with implanted devices were contained in a large-animal transport cart equipped with a squeeze panel. A portable clip-on oximetry device (Vetcorder Pro, Sentier Connect) accomplished external monitoring of SpO_2_ and pulse. Baseline and blood draw procedures remained the same as anesthetized experiments. Fentanyl (30 to 100 μg/kg) was administered at time = 0. Food was withheld until after recovery from overdose.

Upon detecting overdose, the devices autonomously triggered NLX release. In the “control” case, the devices were configured to record StO_2_ but not armed to trigger the rescue operation. The animals were observed for as long as possible until imminent veterinary intervention was required, at which point rescue dose(s) of intravenous NLX were administered. Each rescue dose was 1 mg (1 mg/mL NLX HCl solution). Additional lifesaving measures were supplied as indicated.

#### Longitudinal PK studies (porcine)

Pigs were sedated with telazol (6 mg/kg) and atropine (0.05 mg/kg) and intubated. Inhalational isoflurane (1 to 3%) maintained adequate anesthetic depth. Physiological monitoring devices were placed to monitor heart rate (electrocardiography), SpO_2_ and pulse (PPG), and respiratory vitals (capnography) with a veterinary vitals machine (LifeWindow 6000 V, Digicare Biomedical). Samples for small-molecule PK analysis were collected in EDTA plasma tubes (K_2_EDTA Vacuette, Greiner Bio-One), stored on ice, and centrifuged (3500 rpm, 8 min) within 1 hour of collection.

A baseline blood sample for PK was drawn within the 5 min preceding NLX administration, which occurred at time = 0 via manual pump activation with the mobile application. Blood samples for PK were collected every 1 min for the first 10 min, every 2 to 3 min for the following 10 min, and every 10 min thereafter until time = 60 min. In control intravenous studies, a continuous infusion pump (BeneFusion SP3 Vet, Mindray Animal Care) supplied the same NLX/Gd mixture used in devices at a flow rate of 30 ml/hour. In control injection studies, manual subcutaneous injection of the NLX/Gd mixture occurred at time = 0.

#### Freely moving oximetry (porcine)

A device connected with the mobile application was placed outside of the home cage of a pig with implanted devices. A command from the mobile application started oximetry recording. The implanted device streamed continuously until the recording was stopped or the battery was drained on either the implanted or cellular device. Normal feeding and animal care occurred without disruption during the recording period.

#### Hypoxia challenge experiments (porcine)

The hypoxia procedure was approved by the Institutional Animal Care and Use Committee of Washington University in St. Louis. Female Yorkshire Landrace crossbred pigs (45 to 55 kg) were obtained from Oak Hill Genetics (Ewing, IL, USA). The pigs were sedated, intubated, and brought under general anesthesia with isoflurane (1 to 5%). Carotid arterial and femoral venous lines placed via cutdown allowed for blood sampling. The devices were implanted subcutaneously in the foreleg (deltoid and triceps) and medial rectus abdominis, and StO_2_ recording commenced. Arterial and venous blood gases (ABG and VBG) were measured from time-synchronized blood samples and analyzed with a blood gas analyzer (StatPrime CCS Analyzer, Nova Biomedical). A commercial ear-mounted probe measured SpO_2_, while a skin-mounted commercial probe system (T.Ox, Vioptix) measured cutaneous StO_2_ to compare with device recordings.

Baseline data were collected at normoxic (21% O_2_) conditions, followed by periods of hypoxia induced by modulation of the inspired oxygen fraction (balance: nitrogen). Blood samples for ABG/VBG were drawn many times across the spectrum of inhaled gas mixtures. Oxygen saturation (SO_2_) in capillary blood was defined by a weighted average of venous and arterial SO_2_ (70V:30A) and compared to StO_2_ measured by the implanted device. This experimental approach was partially based on validation studies for an FDA-approved tissue oximetry device (*66*). The hypoxia challenge occurred in two animals, with one animal containing two devices and the other using one. Resulting comparisons between Naloximeter StO_2_ and the other oximetry measurements are plotted in figs. S18 and S19.

#### Cardiac biomarker analysis (porcine)

Samples for cardiac biomarker analysis were collected before and after intravenous Naloximeter activation. Troponin I was quantified with a canine/feline assay (Troponin I, IDEXX Laboratories, Westbrook, Maine, USA). Samples were collected in serum clot activator tubes (CAT Serum Clot Activator Vacuette Tubes, Greiner Bio-One). Preactivation samples were collected at the time of baseline PK blood collection, and postactivation samples were collected between 60 and 65 min after pump activation. Blood samples were incubated at room temperature for 30 min before centrifugation (3000 rpm, 10 min) and then stored at −4°C and shipped the same day to the analytical laboratory. The results of this analysis are in fig. S40.

### Small animal studies

All procedures were approved by the Institutional Animal Care and Use Committee of Washington University in St. Louis. Male and female Sprague-Dawley rats (250 to 350 g) were used for all rodent experiments. The rats were initially group-housed with two to three animals per cage on a 12:12-hour LD cycle (lights on at 7:00 a.m.) and acclimated to the animal facility holding rooms for at least 7 days. The temperature in the holding rooms ranged from 21° to 24°C, while the humidity was maintained between 30 and 70%. The rats received food and water ad libitum throughout experiments, which were conducted during the light cycle.

#### Surgical procedures

The animals were deeply anesthetized using isoflurane (5% induction, 2% maintenance) and administered carprofen (5 mg/kg, subcutaneous, s.c.), enrofloxacin (8 mg/kg, s.c., Baytril), and bupivacaine (5 mg/kg, intravenous catheterization) or lidocaine (8 mg/kg, device implantation) for pre- and intraoperative analgesia. Clippers shaved the surgical site, and three applications of betadine/ethanol swab sterilized the area. A recirculating heated water blanket warmed the animals during surgery.

#### Intravenous catheterization (rodent)

A small incision on the ventral surface of the neck provided access to the jugular vein, wherein an indwelling catheter was inserted and sutured in place with nonabsorbable (6-0 silk) suture. The catheter was tunneled subcutaneously to the dorsal neck and exited via another small incision. The ventral incision in the muscle was closed with absorbable (4-0 vicryl) suture, and the skin incision was closed using nonabsorbable (6-0 silk) suture. The exposed catheter was connected to a backpack device (Vascular Access Harness, Instech) containing a port for drug administration. The rats received carprofen tablets (Rimadyl 2 mg, MD150-2, Bio-Serv) for 2 days after surgery to assist in wound healing and analgesia. A sterile gentamicin/saline solution (0.3 ml at 1.33 mg/ml gentamicin) administered daily maintained catheter patency. The rats were single-housed following surgery and allowed to recover for 1 week before device implantation surgery.

#### Device implantation (rodent)

Ethylene oxide exposure (24-hour cycle) served as the method for device sterilization. A 5-cm incision was made on the back between the shoulder blade and spine to expose the latissimus dorsi and external oblique muscles. Blunt dissection created a subcutaneous pocket sized appropriately to the device. Aseptic technique was used to fill the devices with a prepared NLX/saline solution (6.67 mg/ml NLX hydrochloride dihydrate; pharmaceutical grade, MilliporeSigma) immediately before implantation, and Kwik-Sil sealed the filling port. The device was inserted into the pocket, and a running stitch of absorbable suture (5-0 PDS) was used to make tacking sutures to close the excess subcutaneous space. The skin incision was closed using absorbable (5-0 PDS) subcuticular sutures, and topical tissue adhesive (GLUture, World Precision Instruments) was applied to the incision. Betadine and topical lidocaine ointment were applied over the closed incision for their antiseptic and analgesic properties, respectively. Postoperatively, the rats were treated with anti-inflammatory carprofen (5 mg/kg) and antibiotic enrofloxacin (8 mg/kg, Baytril) once daily for up to 7 days.

#### Hypoxia challenge experiments (rodent)

Hypoxia-induced changes in pulse oxygenation (SpO_2_) and tissue oxygenation (StO_2_) were examined in catheterized rats with implanted devices. A gas blender (Hypoxydial; Starr Life Sciences) regulated the mixture of inhaled gases (O_2_ and N_2_) for precise oxygen concentration. The rats were lightly anesthetized with isoflurane (3% induction, 2% maintenance) and fitted with a pulse oximeter collar (MouseOx v2.0, Starr Life Sciences) to measure SpO_2_ while the implanted device measured StO_2_. Baseline data were collected at normoxic (21% O_2_) conditions, followed by stepwise adjustment in 5-min episodes down to a minimum of 8% O_2_. Additional datasets used in the correlation of SpO_2_ and StO_2_ (Fig. 2H) are included in fig. S17.

#### Fentanyl overdose experiments (rodent)

Rats with implanted devices and catheters were lightly anesthetized with isoflurane (5% induction, 1 to 1.5% maintenance) and fitted with a pulse oximeter collar (MouseOx v2.0, Starr Life Sciences). Cardiorespiratory parameters (oxygen saturation, heart rate, and respiratory rate) were collected throughout the experiment using the collar oximeter and the implanted device. All animals received an intravenous administration of fentanyl citrate (20 μg/kg) at time = 0. After 60 s, the animals in the manual injection treatment group received a subcutaneous injection of NLX (NLX HCl, 1 mg/kg at 6.67 mg/ml concentration; MilliporeSigma). The animals in the closed-loop treatment group had implanted devices that automatically triggered NLX release according to the overdose detection mechanism (see “Automated Rescue Implementation” in supplementary text). The animals in the self-recovery group did not receive any intervention.

### NLX and fentanyl PK quantification

Plasma specimens were analyzed using a modification of a validated liquid chromatography tandem mass spectrometry (LC-MS/MS) assay (*67*). Briefly, 100 μl of plasma was transferred to a conical tube with lid (1.5-ml tube, Eppendorf) and 100 μl of methanol containing the internal standards (10 ng/ml) was added. The samples were vortexed for 3 min and centrifuged for 10 min (26,000*g*, 4°C). Addition of 170 μl of sample to a high-performance LC (HPLC) vial containing 800 μl of water formed the analyzed solution. An LC-MS/MS tandem mass spectrometer (ABSciex 5500, SCIEX) with a turbo V ion source operated in positive electrospray ionization mode quantified fentanyl and NLX in the plasma. An LC system (1200 Series, Agilent Technologies) equipped with a quaternary pump, a temperature-controlled column compartment, and an autosampler (HTC PAL autosampler, Leap Technologies) performed the chromatography. The column (2.6 μm, 3.0 mm by 100 mm Kinetex F5 core-shell column, Phenomenex) separated the analytes using an HPLC flow rate of 1 ml/min with two mobile phases, A: 0.2% aqueous formic acid and B: acetonitrile (LC-MS grade). The column temperature was 50°C. Limits of quantifications were 0.5 ng/ml for fentanyl and NLX. The upper limit of quantification was 1000 ng/ml for fentanyl and 250 ng/ml for NLX.

### Gd PK quantification

PK data collected with the Gd tracer element were analyzed with inductively coupled plasma MS (ICP-MS). Pre-weighed metal-free tubes (MetalFree 15 ml, Labcon) collected 200 to 500 μl of fresh blood per sample and were kept frozen at −20°C until post-weighed and workup began. The blood samples were treated with 0.5 ml of trace-grade nitric acid (>69%, Thermo Fisher Scientific) and 0.5 ml of trace-grade hydrogen peroxide (>30%, GFS Chemicals) and placed at 65°C for at least 3 hours to allow for complete sample digestion. Ultrapure water (18.2 megaohm·cm) was then added to produce a final solution of 5.0% nitric acid (v/v) in a total volume of 10 ml.

Dilution of a quantitative standard (1000 μg/ml Gd elemental standard, Inorganic Ventures) to 500 ng/g element concentration in 5.0% nitric acid (v/v) formed the quantitative standard. A subsequent 100× dilution created a second quantitative standard of 5 ng/g Gd in 5.0% nitric acid (v/v). A solution of 5.0% nitric acid (v/v) was used as the calibration blank.

ICP-MS was performed on a computer-controlled (QTEGRA software) instrument (iCapQ, Thermo Fisher Scientific) operating in STD mode and equipped with an autosampler (ESI SC-2DX PrepFAST, Elemental Scientific Inc.). Internal standard was added inline using the prepFAST system and consisted of 1 ng/ml of a mixed element solution containing Li, Sc, Y, In, Tb, and also carried out inline dilutions to generate a calibration curve consisting of 500, 100, 50, 25, 10, 5, 1, 0.5, 0.25, 0.1, and 0.05 parts per billion of Gd. Each sample was acquired using one survey run (10 sweeps) and three main (peak jumping) runs (40 sweeps). The isotopes selected for analysis were ^156,157^Gd, and ^115^In, ^159^Tb (chosen as internal standards for data interpolation and machine stability). Instrument performance is optimized daily through autotuning followed by verification via a performance report (passing manufacturer specifications).

### Statistical analysis

Animals were randomly assigned to treatment groups. Statistical analyses were performed using Prism (GraphPad Software). Sample sizes are reported as number of animals (*N*) or trials/devices (*n*) in the figure captions. All data are expressed as means ± SD or as individual plots. One-way analysis of variance (ANOVA) was used for multiple group comparisons, and *P* values less than 0.05 were considered significant. The before-after comparison in fig. S40 is a paired, two-sided (conservative) *t*-test. Raw data (λ_1_ and λ_2_) are plotted without smoothing or adulteration. Fast Fourier transform spectra from the raw optical signals were computed with MATLAB (Mathworks Inc.).

## Supplementary Materials

### The PDF file includes:

Supplementary Text Figs. S1 to S40 Tables S1 and S2 Legends for movies S1 and S2 References
