# Supplementary material for: An autonomous implantable device for the prevention of death from opioid overdose
Source: Sci Adv. 2024 Oct 23;10(43):eadr3567. doi: 10.1126/sciadv.adr3567 (PMC11498215; doi:10.1126/sciadv.adr3567)
Supplement: Supplementary file 1 — Supplementary Text Figs. S1 to S40 Tables S1 and S2 Legends for movies S1 and S2 References [file sciadv.adr3567_sm.pdf]

Science Advances
AAAS

Supplementary Materials for

An autonomous implantable device for the prevention of death from opioid overdose

Joanna L. Ciatti *et al.*

Corresponding author: Robert W. Gereau, gereaur@wustl.edu; John A. Rogers, jrogers@northwestern.edu

Sci. Adv. **10**, eadr3567 (2024)
DOI: 10.1126/sciadv.adr3567

The PDF file includes:

Supplementary Text
Figs. S1 to S40
Tables S1 and S2
Legends for movies S1 and S2
References

Other Supplementary Material for this manuscript includes the following:

Movies S1 and S2

Supplementary Text

Wireless BLE Integration with Implanted Device

The implanted device established BLE wireless communication with a cellular device that runs a custom mobile application shown in Fig. S3. The user can send device configuration parameters (optical sensor: sampling rate and optical intensity; pump: pulse width modulation parameters for actuation) or action commands (start/stop oximetry recording, reset device, and manual pump activation with direction control for the needle injector) to the device using the mobile application graphical user interface. The implanted device transmitted two packets of data every one second. The first packet contained one-second worth of raw photocurrent values and the second contained device status indicators (temperature, pump activation status, battery voltage and wireless charging status). Current consumption during various modes of operation was characterized using a power profiler (Power Profiler Kit II, Nordic Semiconductor) and is reported in Fig. S4 and Table S1.

The mobile application sent a timestamp to the implanted device every 300 seconds, synchronized with respect to the coordinated universal time (UTC), to update the internal real time clock of the MCU. Thus, the mobile application received UTC timestamped data packets and uploaded them to a remote server via internet connection. This data management strategy allowed large-scale data collection from multiple devices and subjects simultaneously.

Automated Rescue Implementation

Prior to administration of fentanyl, the implanted devices received the "Start Oximetry" command and began data transmission to the cellular device. Upon detecting a drop in oxygenation due to opioid-induced respiratory depression, the mobile application prompted a WARNING, then triggered the RESCUE operation. First, the mobile application sent the device a command to activate drug delivery via BLE. Then, it relayed an emergency call using the cellular network. In the case of animal experiments, the cellular device dialed an emergency contact set up in the mobile application rather than 9-1-1 due to Federal Communications Commission restrictions on non-emergency 9-1-1 calls. In the case of rodent devices, a MATLAB (Mathworks, Inc.) script replaced the mobile application for device control and data collection.

Theoretical Modeling of Drug Delivery with Electrolytic Pump

We employed an analytical model for electrolytic drug delivery microsystems, derived from singular perturbation methods in our previous work (*68*), to predict the delivery time over a range of geometrical and elasticity parameters in the flexible membrane. The model used a combination of normalized non-dimensional device parameters, including the initial environmental pressure in the body P_0^*, initial volume of gas in the electrolyte chamber V_0^*, and microfluidic resistance based on the microfluidic layouts M^* to characterize the drug delivery process and predict the drug delivery time. For a negligible V_0^*, small M^*, and known initial environmental pressure P_0^*, the

relationship between the time t^* and drug volume V^* is given by the following non-dimensional parametric expression:

$$t^* = V^*[G(V^*) + P_0^*] + M^* \frac{V^*}{\frac{d}{dV^*}[V^*G(V^*)] + P_0^*} \quad \text{... (S1)}$$

where $G(V^*)$ is the normalized pressure-volume function describing the deformation of the flexible membrane. Equation S1 can be re-written dimensionally as

$$t = \frac{4F}{3RTi} V[f(V) + P_0] + \frac{32\mu L}{a^4} \frac{V}{\frac{d[Vf(V)]}{dV} + P_0} \quad \text{... (S2)}$$

where F is Faraday's constant, R is the ideal gas constant, T is temperature, i is the electrical current applied to the electrodes, $f(V)$ is the pressure-volume function of the flexible membrane, μ is the drug viscosity, L is the length of the microfluidic channels, and a is the side length of the channel.

The first term in Eq. S2 quantifies the time required for the membrane to overcome the external pressure and deform into a spherical cap and the second term quantifies the time required for the drug to travel through the microchannels. In this work, microchannels are exchanged for an intravenous catheter, which has characteristic length an order of magnitude larger. In this regime, the microfluidic resistance becomes negligible, and instead the "dead volume" (V_{DV}) inside of the catheter tubing becomes significant. Thus, we rewrite Eq. S2 to account for the intravenous device configuration as:

$$t = \frac{4F}{3RTi}(V_d + V_{DV})[f(V_d) + P_0] \quad \text{... (S3)}$$

Where the volume delivered to the bloodstream ($V_d = V - V_{DV} = V - \frac{\pi D^2 L_c}{4}$) is the difference between the volume of gas generated by electrolysis (V) and V_{DV} for a given the catheter internal diameter (D) and length (L_c).

The deformation of the membrane from flat to spherical cap (for finite-deformation) is approximated for a linear-elastic material model as

$$f(V_d) = \frac{64Eh}{3\pi^3 R_0^{10}(1 - v)}(V_d + V_{DV})^3 \quad \text{... (S4)}$$

where E is the Young's Modulus, h is the thickness, v is Poisson's ratio, and R_0 is the radius of the membrane. A parametric study, shown in Fig. S9, was performed to model the effect of varying

the Young's Modulus (E = 1 – 3.5 MPa), thickness (h = 40 –150 μm), and radius ($R_0 = 10 - 15\ \mathrm{mm}$) of the flexible membrane in the time required to deliver 1000 μL of drug. This was substantiated by experimental evidence tracking the membrane deformation versus time, provided in Fig. S10.

Preliminary Studies with Cutaneous Naloximeter Sensors for Humans

Cutaneous Naloximeter sensors (oximeters) shared the electronics of the large animal devices but omitted the drug delivery pumps. Two-sided f-PCBs, containing a planar coil for wireless recharging, supported electronic components on one side and a battery on the other. The same firmware, logic, and mobile application was used in both cases. The key components (MCU, LiPo battery, dual-wavelength optical sensor) and fabrication processes were identical with the electronics module of the large animal Naloximeter. Encapsulation of the devices followed a similar PDMS molding process as described in the methods. For measurement sessions, sensors (oximetry and accelerometry) were secured to the skin of the volar forearm with a skin-safe adhesive (KM 40A, Katecho) and optionally covered with a layer of film dressing (Tegaderm, 3M, Inc.).

Human studies were performed according to the protocol approved by the Northwestern University Institutional Review Board (IRB #: STU00220375). Informed consent was obtained prior to participation. Collection of marked data from an active movement sequence began with baseline data recording for several minutes. The active sequence consisted of a series of movements: finger pressing on the device, running, walking, biking, aerobic exercise (e.g. jumping jacks), and resting (seated and supinated). Start and stop times for each exercise were noted, as well as any observations of physiological condition.

Overdose Detection Algorithm (ODA)

The overdose detection algorithm (ODA) is founded on the temporal dynamics of physiological signals characteristic to changes in systemic oxygenation. Measurements of the optical absorption of biological tissues allow for quantification of the oxygenated and deoxygenated hemoglobin species, and thus estimation of oxygenation percentage (*69*). Wearable and implantable devices with strategically engineered sensors have demonstrated this optical technique for interrogating biological metrics (*70–73*). While the optical approach is very sensitive to changes in hemoglobin oxygenation *in vivo*, the underlying mathematical model is blind to confounding events produced by motion artifacts or postural changes that disrupt blood flow in tissue surrounding the sensor. These events can lead to calculation of false fluctuations of oxygenation. Furthermore, obstructive sleep apnea represents a confounding affliction that produces real desaturation events of -20% SpO_2/min lasting ~25 – 30 s, occurring in a characteristic oscillatory pattern (*74–77*). In a study using cerebral oximetry to determine StO_2 during apnea-hypopnea events, the StO_2 desaturation was determined to be -4.02 ± 0.06% in events 12 – 16 s in duration (*77*). Our ODA was designed to discriminate against confounding artifacts that might lead to false overdose detections, such as

sleep apnea or motion. The decomposition of the temporal data collected from the dual-wavelength optical sensor permits the extraction of oxygenation level, its temporal changes and motion indices to produce a series of four logical metrics (M1 – M4). The collective multivariable analysis yields a robust set of data analytics that identifies the physiological signatures of an overdose in real-time.

The ODA received packets of data at 1 Hz containing the two raw optical signals, red (λ_1) and infrared (λ_2). The ODA operates at this frequency to perform calculations on the current and preceding nine packets of data. A digital low-pass Butterworth filter (0.1 Hz, 4th order) processed the incoming raw data to calculate the StO_2 using Eq. 1 and Eq. 2 and the differential optical signal ($\Delta\lambda = \lambda_1 - \lambda_2$). In parallel, a fast Fourier transform (FFT) calculated the power density spectrum on both red and infrared signals filtered using a band-pass Butterworth filter (passband 1-5 Hz, 4th order). The average of the integrated power density spectra provided the power density spectrum index ($PDSi$). Summation of the $PDSi$ over the previous 1 and 10 minutes calculated the short-term and long-term motion indices, $STMi$ and $LTMi$, respectively. Linear interpolation of λ_1, StO_2, and $\Delta\lambda$ followed by differentiation provided the rates of change ($\frac{d}{dt} = \partial$) of the red signal ($\partial\lambda_1$), optical difference ($\partial\Delta\lambda$) and tissue oxygenation (∂StO_2), calculated every 10 seconds over a 60-second window. A comparison of these rates, the StO_2 and the $STMi$, against experimentally derived threshold values produced four logical metrics:

$$\begin{bmatrix} \text{M1} \\ \text{M2} \\ \text{M3} \\ \text{M4} \end{bmatrix} = \begin{bmatrix} (\partial\lambda_{1-ThL} < \partial\lambda_1 < \partial\lambda_{1-ThU}) \,\&\, (\partial\Delta\lambda_{ThL} < \partial\Delta\lambda < \partial\Delta\lambda_{ThU}) \\ \partial StO_{2-ThL} < \partial StO_2 < \partial StO_{2-ThU} \\ \text{StO}_2 < StO_{2-Th} \\ STMi < STMi_{Th} \end{bmatrix} \quad \text{... (S5)}$$

where the subindices in M1 and M2, ThL and ThU, correspond to the lower and upper thresholds, respectively, and StO_{2-Th} and $STMi_{Th}$ to the tissue oxygenation and short-term motion index thresholds, respectively.

An OD-relevant event (EVENT) existed when the mutually inclusive condition of the four metrics in Eq. S5 in the ODA was prompted, which evolved into a WARNING after N consecutive EVENTs: $N = 3$ if $LTMi > LTMi_{Th}$, corresponding to active/awake state; or $N = 6$ if $LTMi < LTMi_{Th}$, corresponding to rest/sleep state. Upon triggering a WARNING, the ODA prompted the user with a push notification to override the warning, if acknowledged. If one additional EVENT accumulated in the 10 seconds following WARNING without user feedback, the ODA triggered the RESCUE response: activating drug delivery and relaying an emergency call. Figure 3H shows the graphical illustration of the workflow associated with these data analytics.

The following thresholds were used for the metrics:

M1	$\partial\lambda_{1-ThL} = -500$	$\partial\lambda_{1-ThU} = -10$	$\partial\Delta\lambda_{ThL} = -500$	$\partial\Delta\lambda_{ThL} = -10$
M2	$\partial StO_{2-ThL} = -100$	$\partial StO_{2-ThU} = -2$		
M3	$StO_{2-Th} = 60$			
M4	$STMi_{Th} = 50$			

These values were determined as follows. The thresholds for $\partial\lambda_1$, $\partial\Delta\lambda$, ∂StO_2 were extracted from data collected during the fentanyl dosage desaturation experiments (e.g. Fig S21 and S25). The 60% threshold for StO_2 corresponds to a physiological level that indicates hypoxia and is equivalent to ~ 80% SpO_2 (Fig. 2H) or ~ 60% SO_2 (Fig. 2I). Finally, the threshold for the $STMi$ is estimated from marked data in human during rest (Fig. S25) and in pigs during rest/sleep (Fig. S27 and S29).

Discussion of Catheter Patency

Catheter patency is critical to the intravenous Naloximeter platform, and it is achieved with two key aspects of the catheter and implantation design. First, the catheter locking solution, which was shown by Luo et al. to maintain patency for up to 4 weeks in rodent models (*65*). No catheter obstructions that prevented infusion were observed at the time of euthanasia (between 21 to 45 days post-implantation). Secondly, the catheter employed in the devices is a silicone catheter containing a normally closed Groshong valve that opens for infusion or aspiration upon positive or negative pressure, respectively (*58*). This prevents backflow of blood into the catheter, and thereby clotting. These types of catheters are often used in treatment with 1 to 3 year duration, and generally exhibit low risk of thrombosis or other complications (*78*). To increase the sophistication of the platform for use in humans, one could imagine a customized catheter with a bursting valve at the vascular tip which isolates the catheter channel from blood until the pump activates, at which point the seal would be broken by positive pressure from the pump, and the drug would infuse into the bloodstream.

Discussion of Battery Life

Battery lifetime is calculated with the simple formula in Eq. S6.

$$Lifetime\ (\mathrm{h}) = \frac{Capacity\ (\mathrm{mAh})}{Load\ Current\ (\mathrm{mA})} \quad \ldots (S6)$$

In the case of continuous oxygenation sensing, i.e. sensor always ON, the lifetime is computed as 2.1 days given the battery capacity of 75 mAh, average load current = 1.461 mA (Table S1). For translation, an intermittent operational mode would be the most beneficial, wherein the sensor is

turned ON and OFF regularly, providing data at regular intervals with lower power consumption (Fig. S4D). For a 20% duty cycle and a 5 s period, the battery life is extended to 6.9 days using the same 75 mAh battery, average load current = 0.452 mA. A larger rechargeable battery with 850 mAh capacity would extend the battery life to 24 days in continuous mode or 11 weeks in the intermittent mode. One example of such a battery is Part No. LP603443JU from Jaunch Battery solutions, with dimensions 46 x 34.5 x 6.2 mm^3.

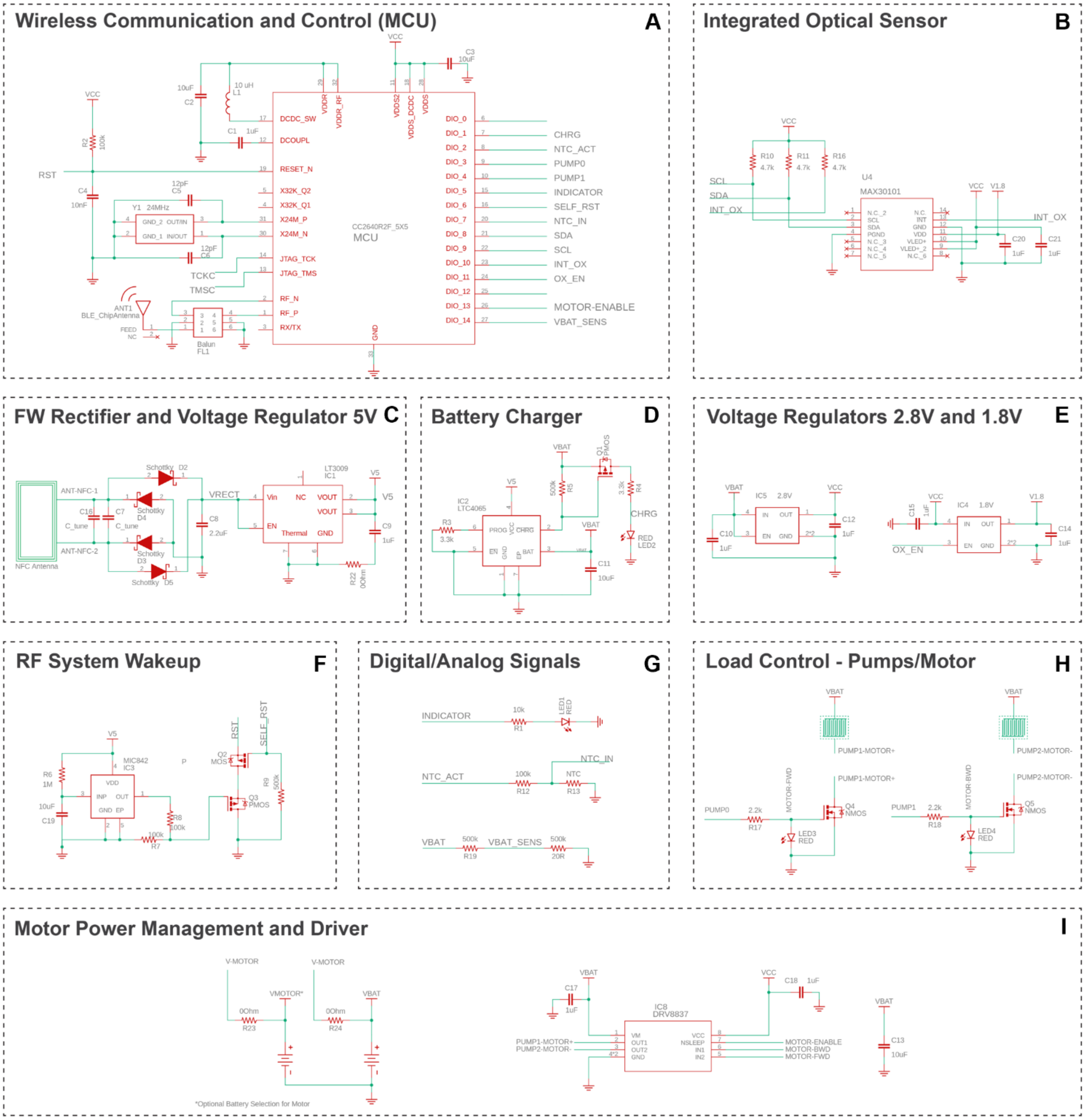

Fig. S1. Circuit diagrams for subsystems of the Naloximeter platform for large animals. (A) The CC2640R2F microcontroller (MCU) uses BLE wireless communication and controls the operation of the device. **(B)** The integrated optical sensor MAX30101 connects with the MCU using I^{2}C serial communication. **(C)** A full-wave (FW) voltage rectifier and regulator converts the harvested input AC to 5 V DC. **(D)** The battery charger is powered by wireless power transfer. **(E)** The step-down voltage regulator supplies 2.8 V to the electronics (left). A 1.8 V regulator provides voltage to the optical sensor only (right). **(F)** An RF actuated wake up system provides a remote

reset to the microcontroller in the event of a failed cold start. **(G)** General inputs/outputs control function in the device such as analog battery voltage monitoring, indicator operation, input NTC sensor. **(H)** The power module controls the electrolysis-driven pumps, or the logic signals to operate the H-bridge chip that drives the DC motor. **(I)** The power and control module operates to the DC motor.

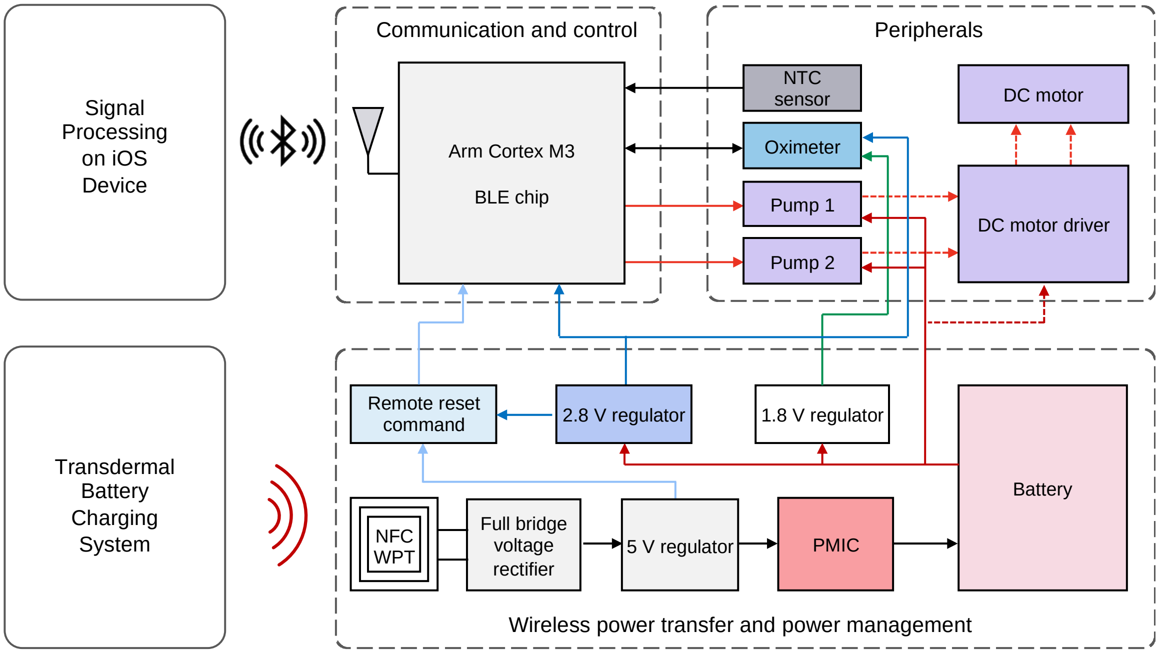

Fig. S2. Functional block diagram of the Naloximeter platform for large animals. Modules that construct the electronic system and its interaction with the external iOS device and transdermal battery charging system. The same system, with minor modifications, supports two options for drug delivery, electrolytic pump or DC motor (dashed lines) operation.

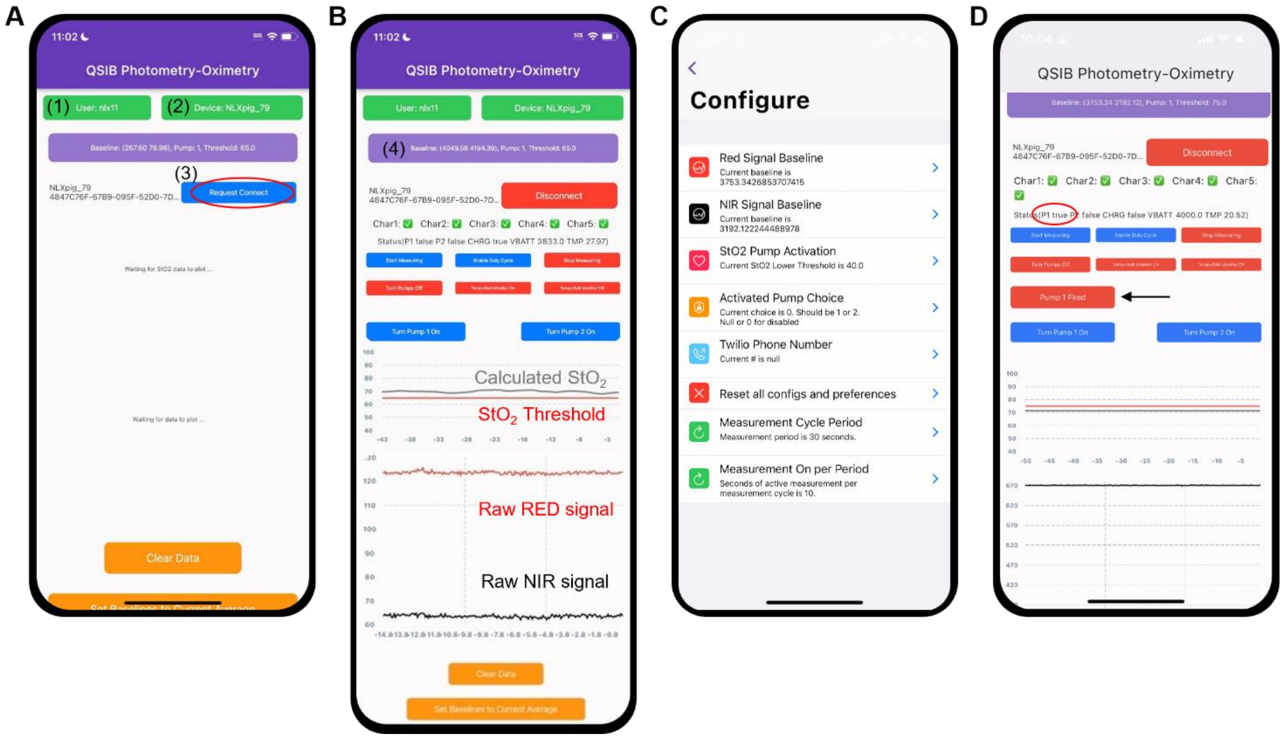

Fig. S3. Screenshots of the mobile application on a phone. (A) The main app screen indicates (1) cloud account for data management, (2) device selection, and (3) button for establishing a BLE connection to the device. **(B)** View of the main app screen after establishing device connection, with buttons for controlling device operation, including collection of optical sensor data and calculation of StO_2 in real-time. **(C)** Device configuration window, accessed by clicking on (4). **(D)** Main app screen provides two indicators of NLX deployment: pump (P1) status (red circle) and "Pump 1 Fired" display (black arrow).

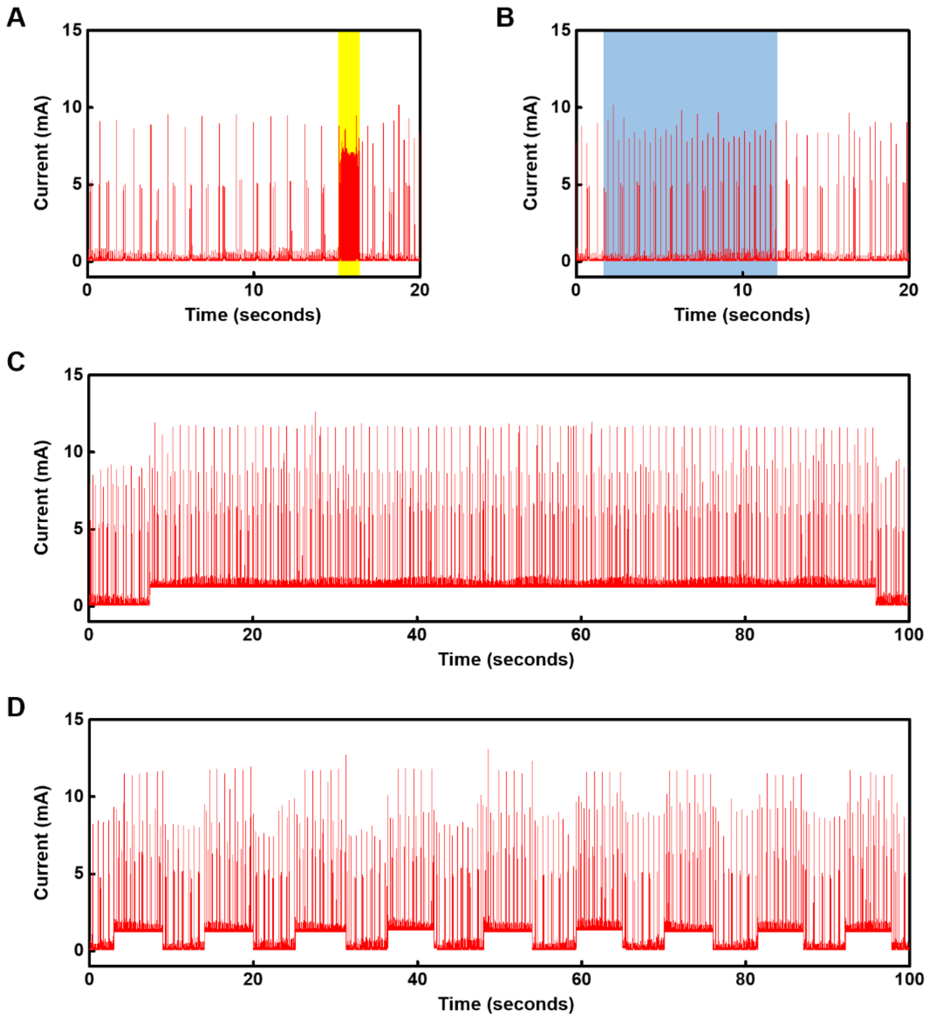

Fig. S4. Current consumption during operation of a Naloximeter for large animals. (A) Current consumption of the device before, during (yellow area), and after establishing BLE connection to the peripheral device. **(B)** Current consumption of the device while sending status updates (temperature, battery voltage, pump status, and charging status) at intervals of 1 s (blue area) and while idle. **(C)** Current consumption of the device while operating the dual-color optical sensor in normal mode. **(D)** Current consumption of the device while operating the dual-color optical sensor in intermittent mode (50% duty cycle, 10 s period: 5 s ON, 5 s OFF). Supply voltage is 3.8 V in all cases.

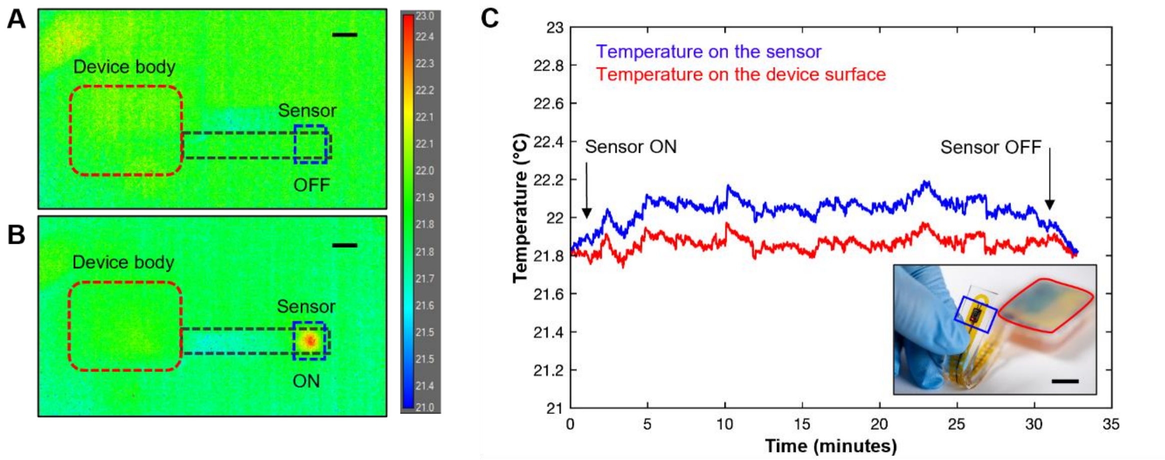

Fig. S5. Benchtop characterization of thermal load produced by the dual-wavelength optical sensor during operation. Thermographic images of an encapsulated test of a device with **(A)** the sensor OFF and **(B)** sensor ON at room temperature. Scale bars, 1 cm. **(C)** Surface average temperature recorded in the area above the electronic board and on the sensor. Inset: optical image of the device used in this characterization study, with sensor separated from the body of the device. Scale bar, 1 cm.

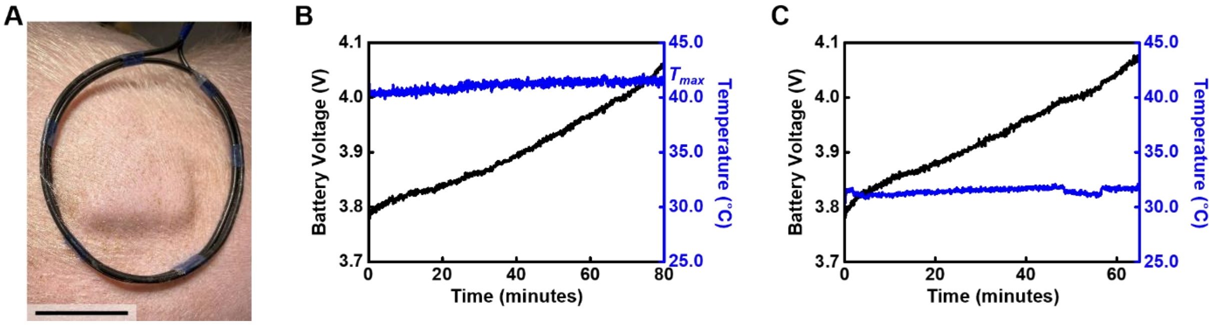

Fig. S6. Transdermal charging of a Naloximeter device for large animals. (A) Photograph showing wireless transdermal charging of a device implanted in a pig model after healing. Scale bar, 5 cm. **(B)** Battery voltage and temperature measured by the NTC in the device during transdermal charging, maximum temperature labeled as T_{max}. **(C)** Battery voltage and temperature measured by the NTC in the device during charging on the benchtop.

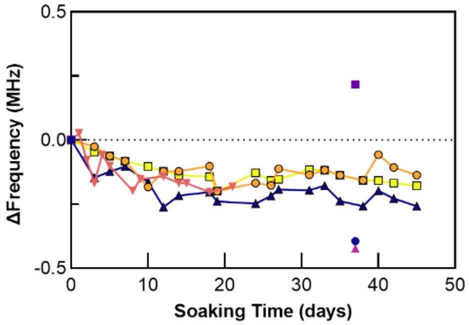

Fig. S7. Survivability of Naloximeter device electronics platform during accelerated aging at 50°C in phosphate buffered saline. Evaluation of device performance by measurement of RF coil resonant frequency, plotted as change over time. Based on acceleration factor aging, 45 days at 50°C corresponds to ~110 days at 37 °C. N = 7 devices, each color represents one device.

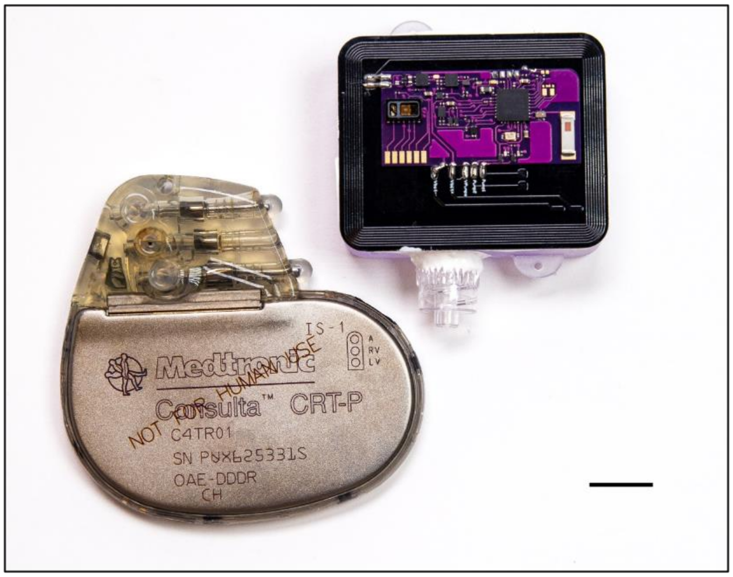

Fig. S8. Optical image of a Naloximeter and a commercial pacemaker (Consulta, Medtronic). Scale bar is 1 cm.

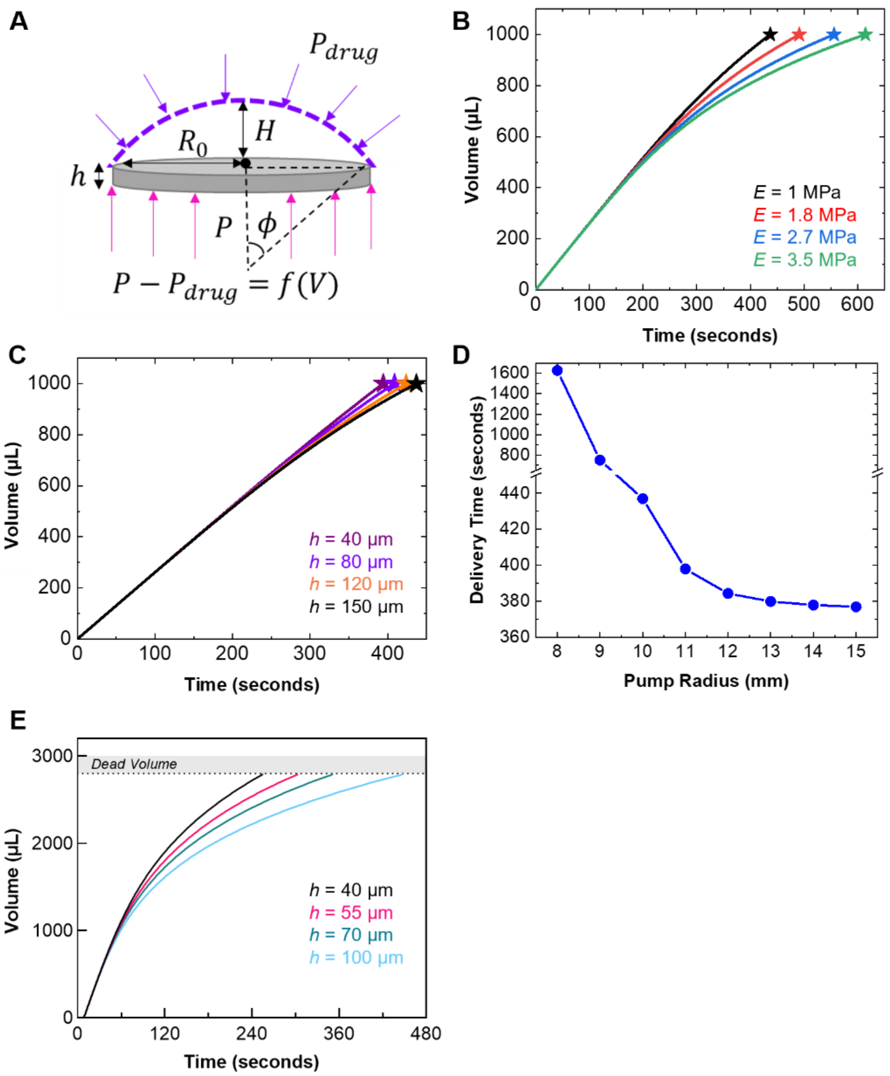

Fig. S9. Parametric FEA modeling of the operation of electrolytic pumps. (A) Schematic diagram of forces on the membrane relevant to these simulations. **(B)** Volume vs. time curves for 1 mL pumps varying the modulus of elastic membrane (h = 1 µm). **(C)** Volume vs. time curves for 1 mL pumps varying the membrane thickness (E = 1 MPa). **(D)** Effect of pump radius on delivery time for 1 mL drug volume, (E = 4.3 MPa and h = 70 µm). Calculations in (A – D) run with constants: i = 15 mA, T = 310 K. **(E)** Evaluation of the effects of membrane thickness for 3 mL pumps using parameters measured in actual devices, i = 120 mA and E = 4.3 MPa.

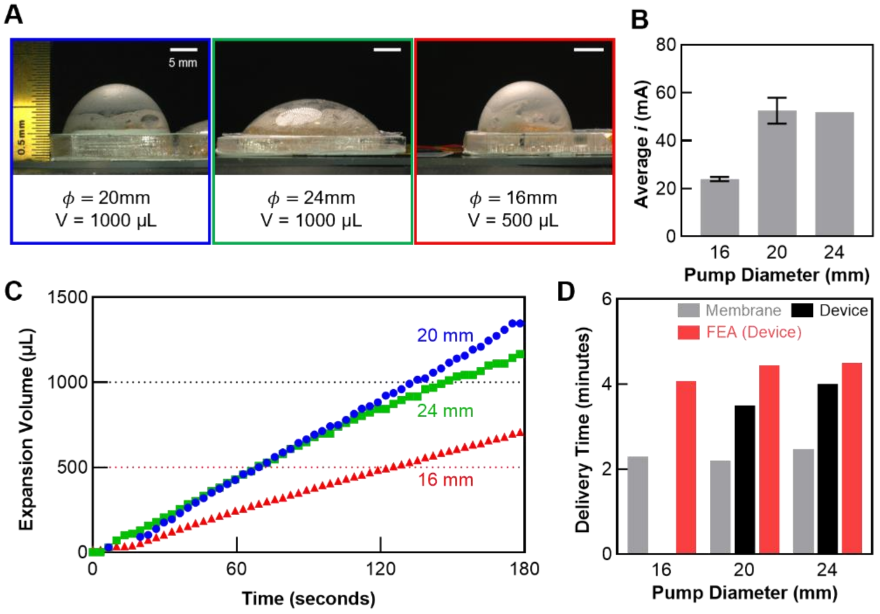

Fig. S10. Benchtop studies of the effects of pump geometry on the profile of membrane deformation. (A) Photograph of three electrolytic pumps with different geometries at inflated states. Scale bars, 5 mm. **(B)** Average current consumption for pumps with different diameters, recorded during electrolysis with the device PCB and battery. **(C)** Expansion volume calculated by image analysis as the membrane deforms during electrolysis. **(D)** Estimated time to deliver the total drug reservoir content according to benchtop studies of the membrane deformation, full device testing, and FEA modeling *(E* = 4.3 MPa, *h* = 70 μm, *T* = 310 K, and *i* from experiment).

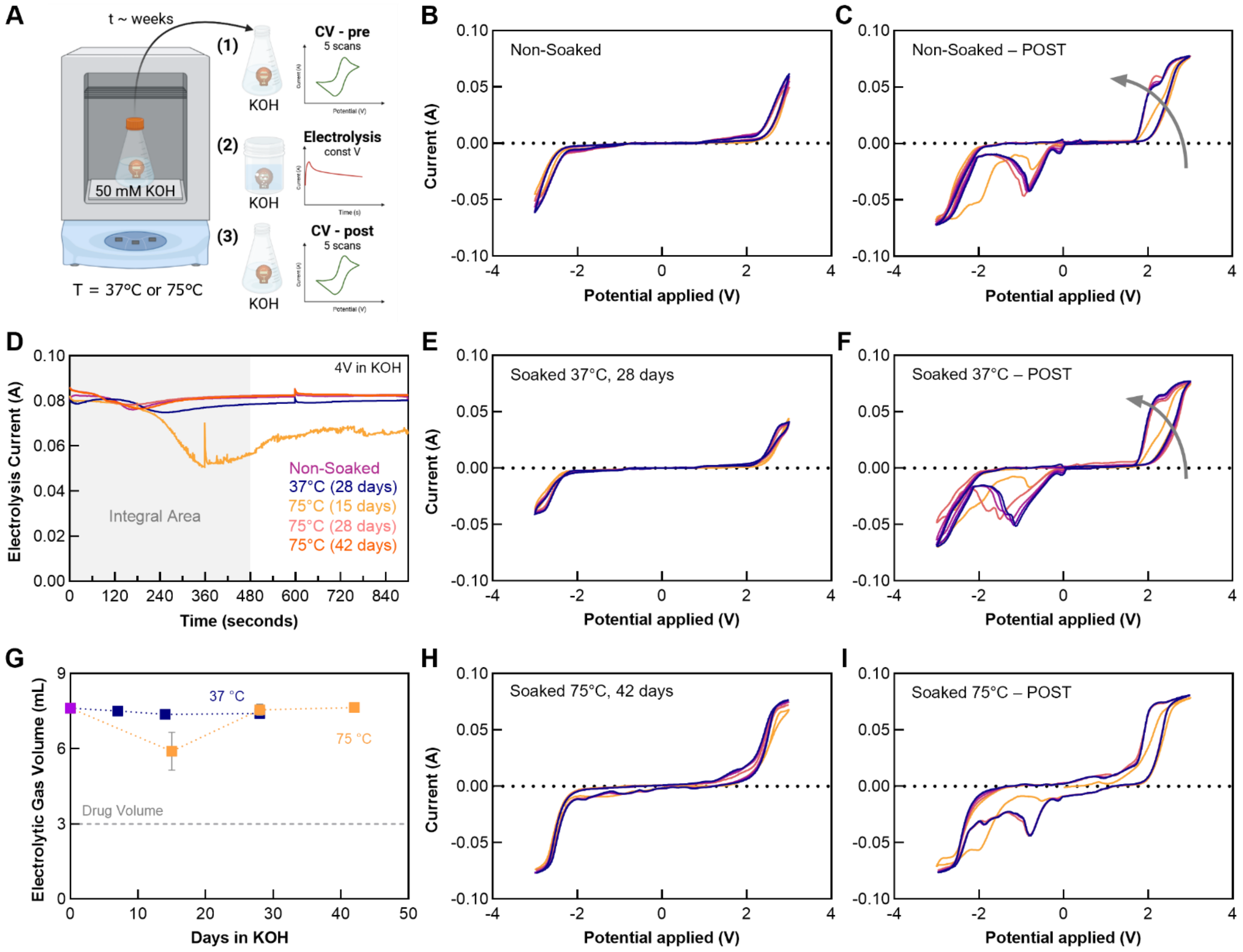

Fig. S11. Benchtop studies of electrode degradation. (A) Schematic illustration of the experimental setup and study design. The experiments consisted of soaking in potassium hydroxide electrolyte (50 mM KOH) at elevated temperature (37°C or 75°C) for several weeks followed by *(1)* cyclic voltammetry (CV), *(2)* constant voltage electrolysis, and *(3)* post-electrolysis CV. All CV tests involved scans of voltage between +/- 3 V at a rate of 50 mV/sec for 5 cycles. Constant volage electrolysis testing at an applied potential of 4 V. All electrochemical characterization was carried out in the electrolyte solution, 50 mM KOH. Cyclic voltammetry plots for a fresh non-soaked control electrode **(B)** before and **(C)** after electrolysis. **(D)** Electrolysis current at constant voltage for control and aged electrodes. Cyclic voltammetry plots for an electrode aged at 37°C for 28 days **(E)** before and **(F)** after electrolysis. **(G)** Volume of gas generated by electrolysis, as calculated with Faraday's Law and measured current (panel D) plotted according to aging time and temperature. Cyclic voltammetry plots for an electrode aged at 75°C

for 42 days – equivalent to 1 year and 9 months at body temperature, **(H)** before and **(I)** after electrolysis.

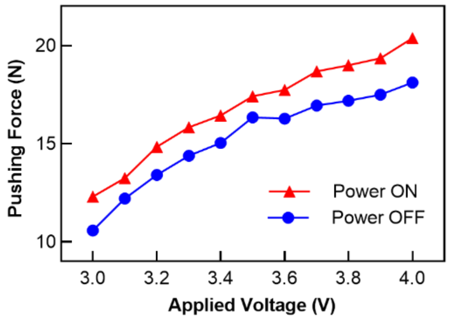

Fig. S12. Force supplied by the plunger and DC motor as a function of applied voltage. Each data point corresponds to the saturated force with the maximum load on the motor.

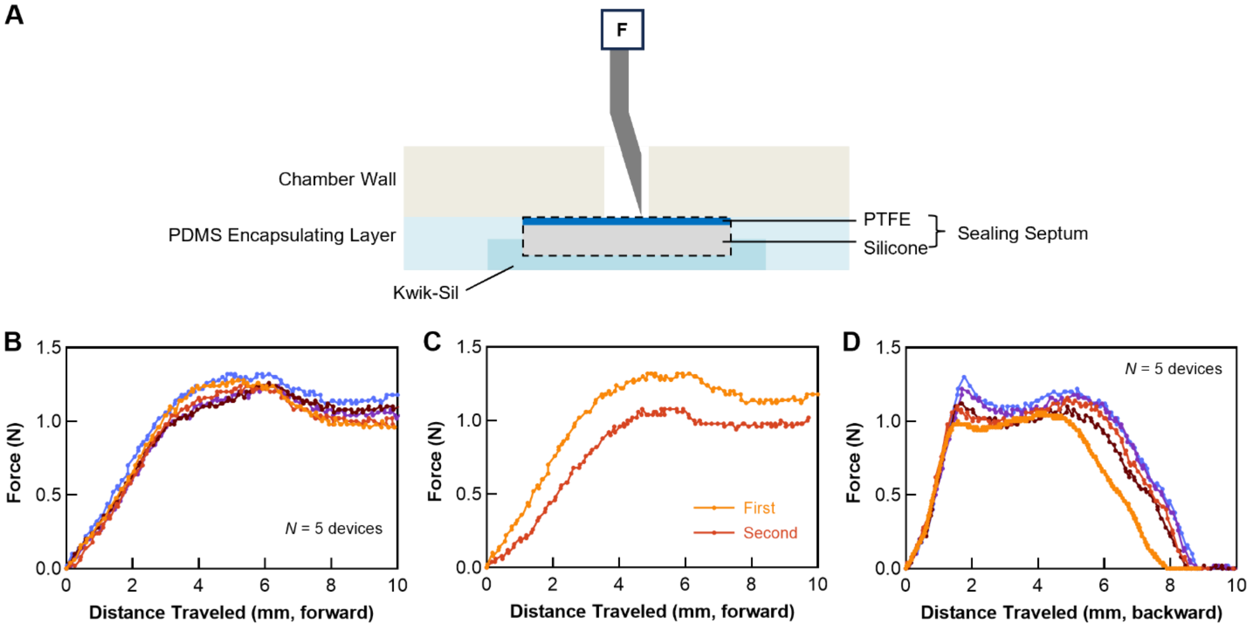

Fig. S13. Force to deploy and retract the Huber needle through the sealing septum. (A) Schematic illustration of the setup to measure the force to deploy and retract the needle through the sealing septum. **(B)** Required force for the needle to pierce the front sealing septum. **(C)** Required force for the needle to pierce the front sealing septum for the first and second time. **(D)** Required force to retract the deployed needle through the sealing septum.

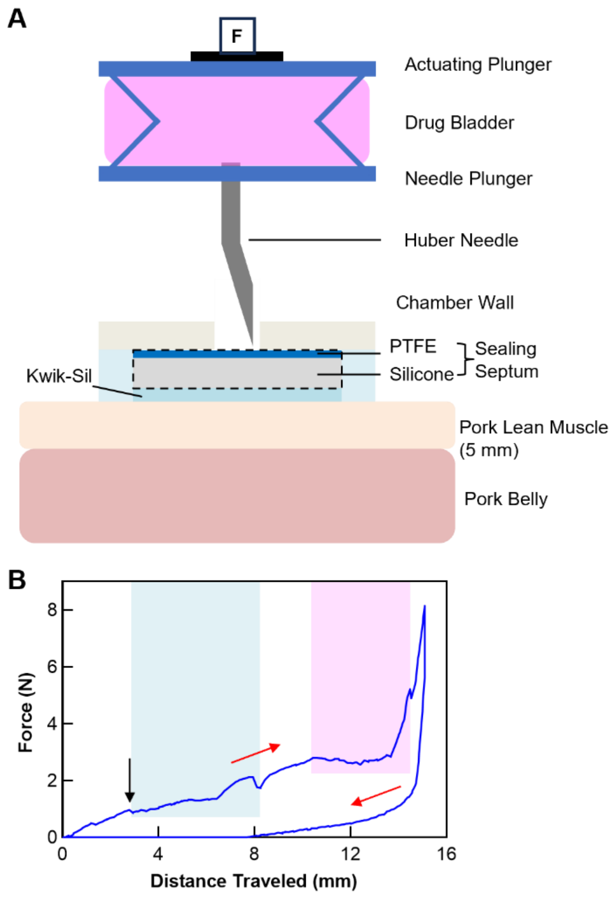

Fig. S14. Force during a successive sequence of deploying the needle, injecting the solution, and retracting the needle. (A) Schematic image of the experimental setup. The bladder is filled with 1.5 mL of solution for this characterization. **(B)** Plot of force during a successive sequence of deploying the needle into a two-layer pork tissue model, injecting the solution, and retracting the needle. Red arrows indicate the progression of time. The black arrow indicates completion of the process of piercing the sealing septum. The blue area corresponds to the regime for piercing two layers of tissue. The pink area corresponds to the regime for injecting the solution from the bladder into the pork belly.

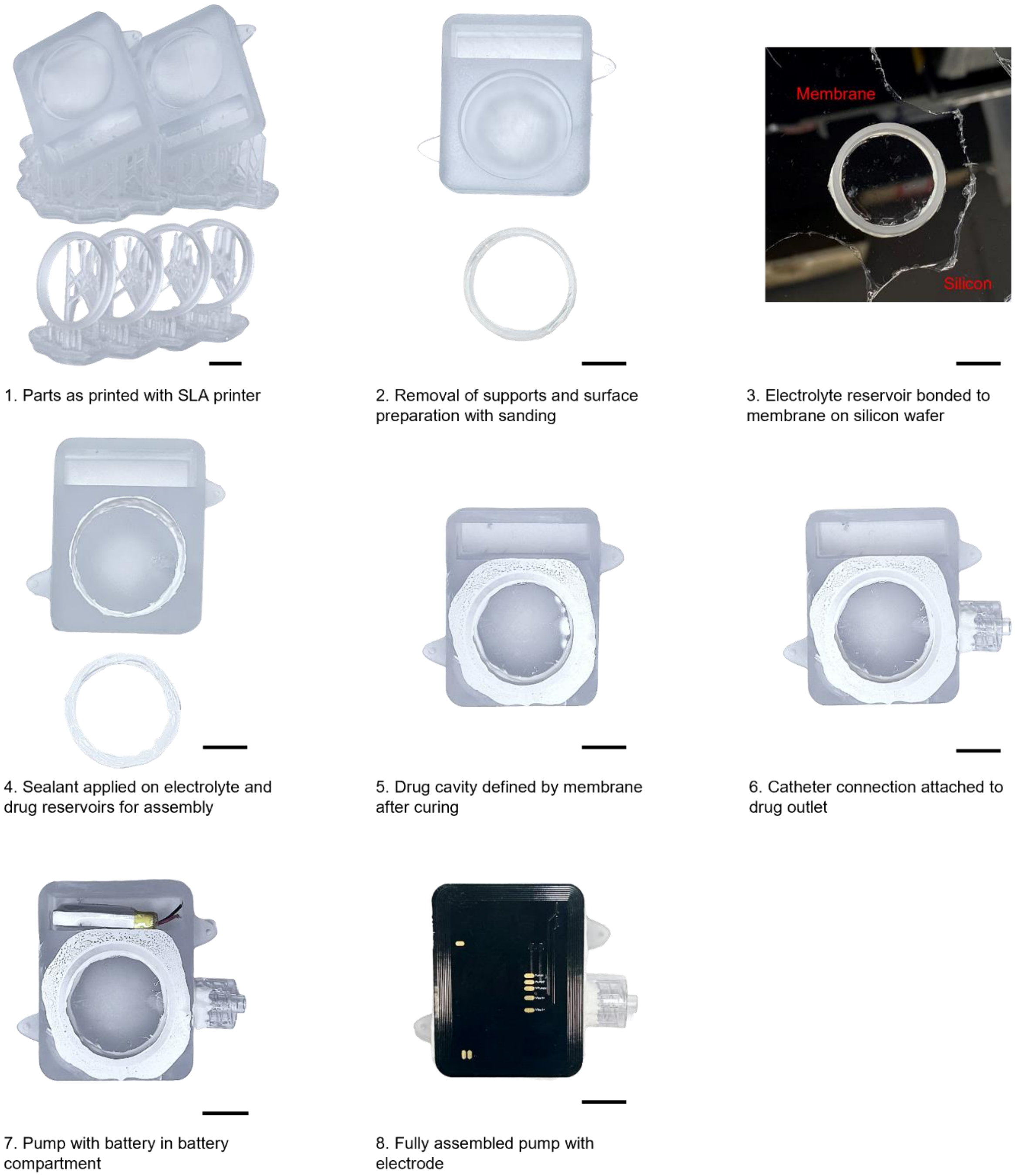

Fig. S15. Steps for fabricating intravenous Naloximeter devices. Scale bars, 1 cm.

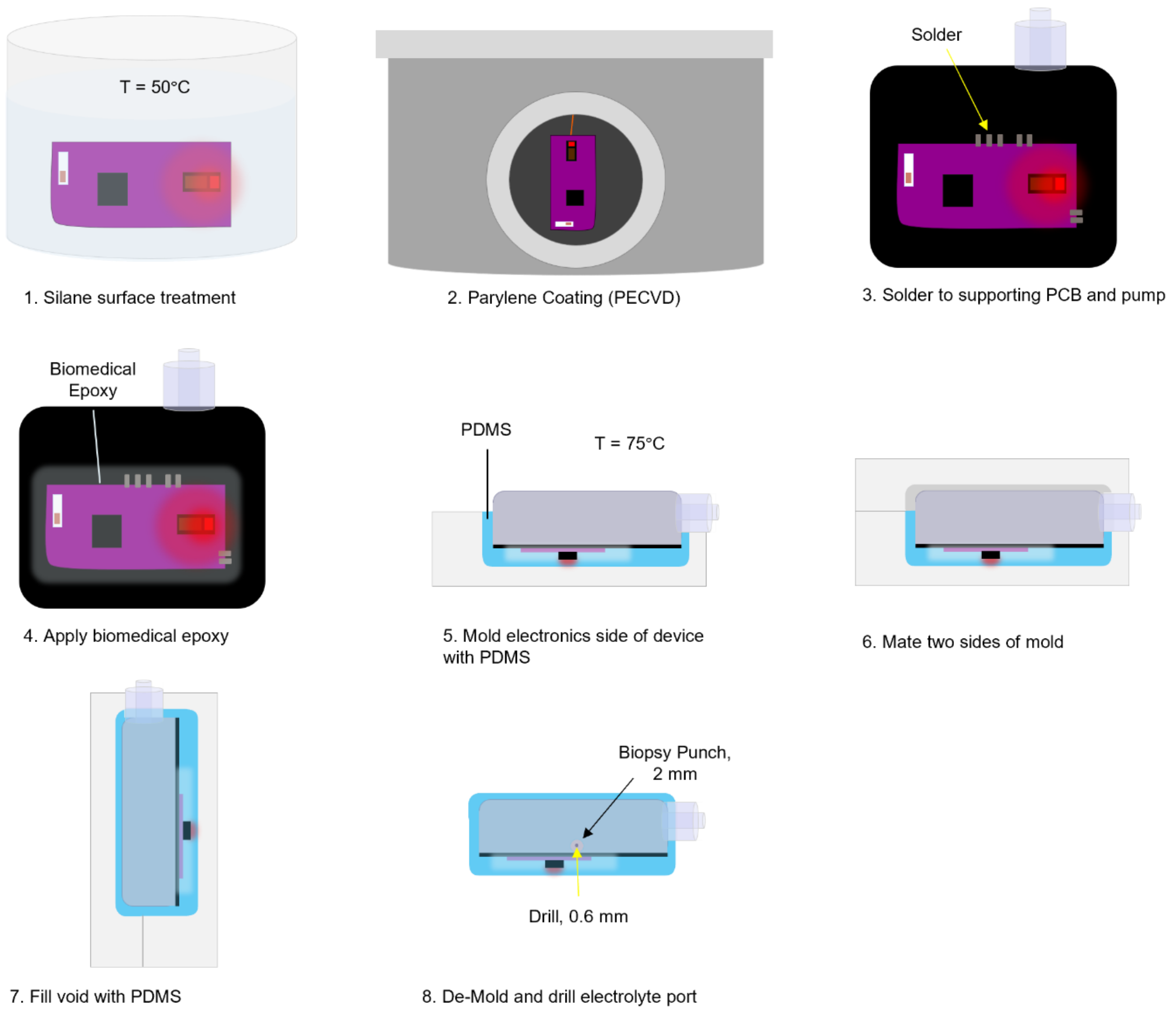

Fig. S16. Steps for encapsulating Naloximeter devices, illustrated with the intravenous device.

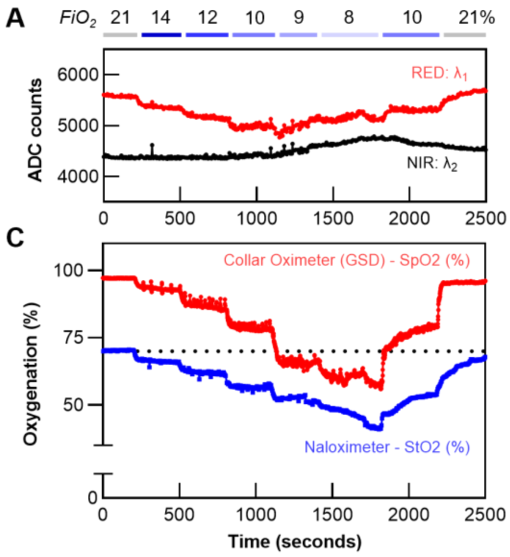

Fig. S17. Hypoxia studies in rodent models. (A, B) Raw red (λ_1) and near-infrared (NIR, λ_2) optical signals from an NFC Naloximeter during hypoxia induced by modulation of the fraction of inspired oxygen (FiO_2). **(C, D)** Corresponding calculated StO_2 from the Naloximeter and SpO_2 measured by a gold standard device (GSD, collar oximeter). Each column represents one animal.

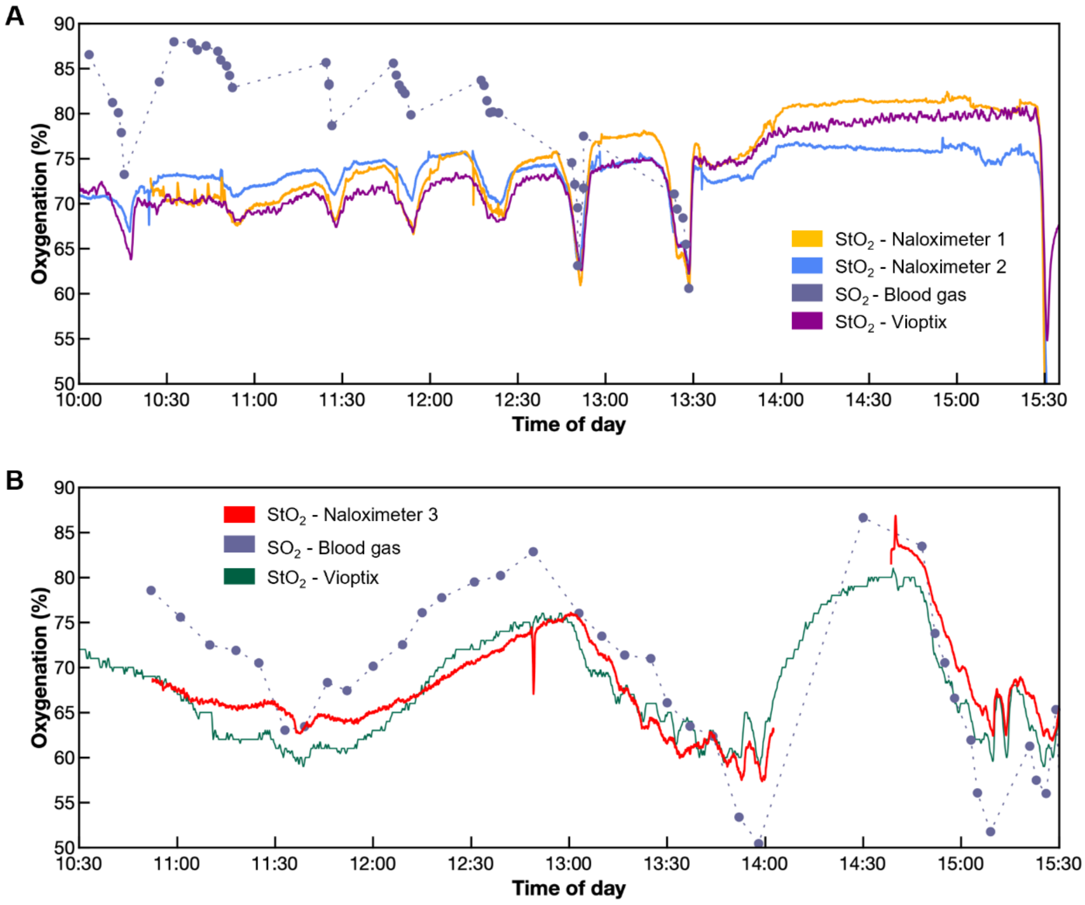

Fig. S18. Hypoxia studies in a porcine model with comparison to standard clinical devices and measurements. (A, B) Blood gas saturation is calculated as a mixture of 70% venous and 30% arterial blood. Devices were placed subcutaneously on the following muscles: Naloximeter 1 = deltoid, Naloximeter 2 = triceps, Naloximeter 3 = rectus abdominus.

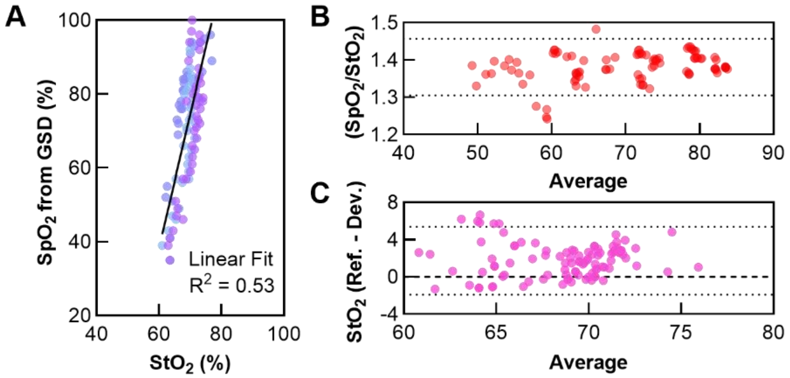

Fig. S19. Extended analyses of hypoxia studies in swine and rodent models. (A) Comparison of SpO_2 measured with gold standard device (GSD, clip-on pulse oximeter) and StO_2 measured with a Naloximeter during hypoxia in a porcine model. **(B)** Bland-Altman plot to compare (ratio versus average) SpO_2 measured with gold standard device and StO_2 measured with the Naloximeter. Dashed lines depict the 95% confidence interval. **(C)** Bland-Altman plot to compare (difference versus average) StO_2 measured with a clinical device (Vioptix) and StO_2 measured with the Naloximeter in porcine model. Dashed lines depict the 95% confidence interval.

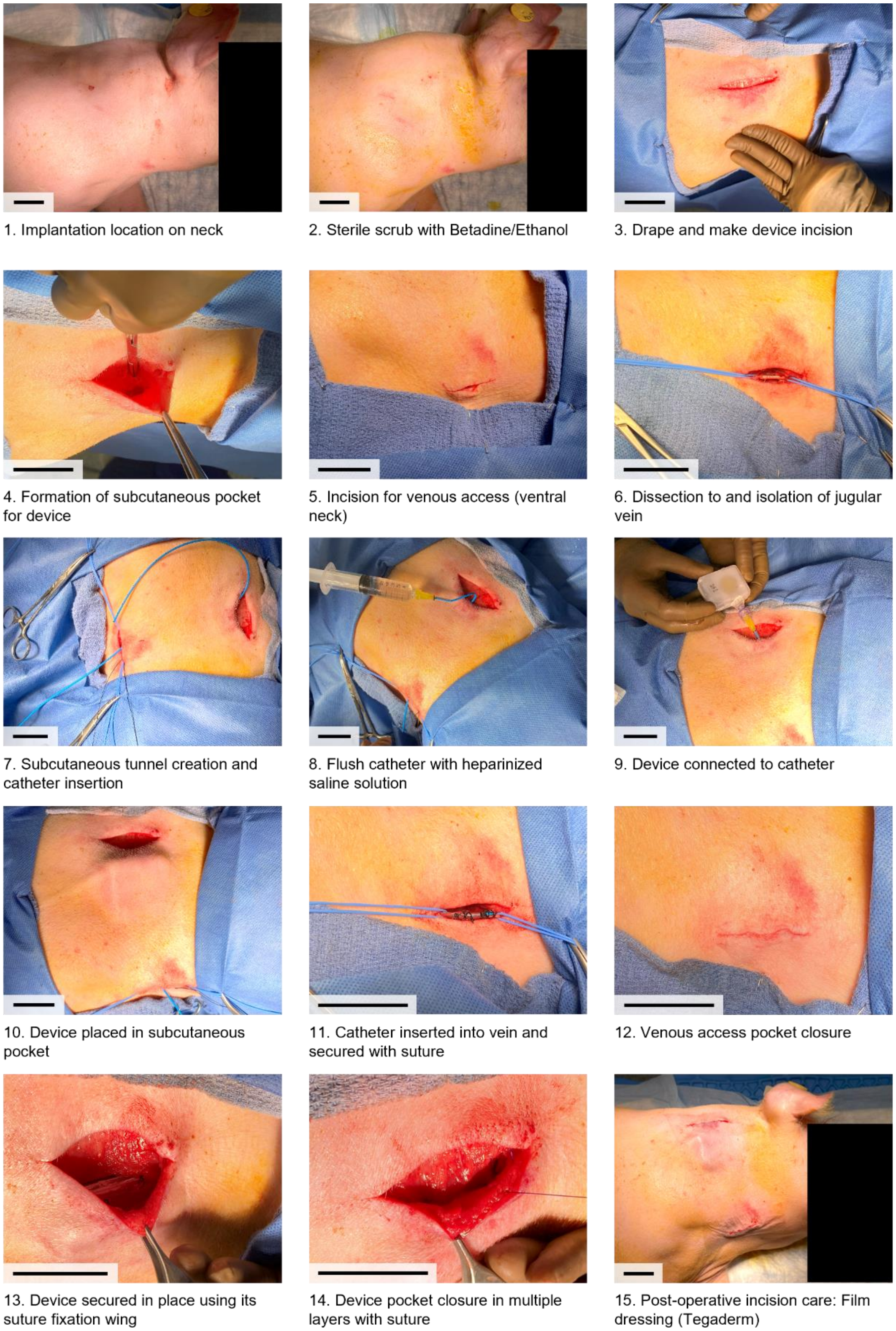

Fig. S20. Surgical approach for implanting an intravenous Naloximeter device in the external jugular vein of a pig. Scale bars, 5 cm.

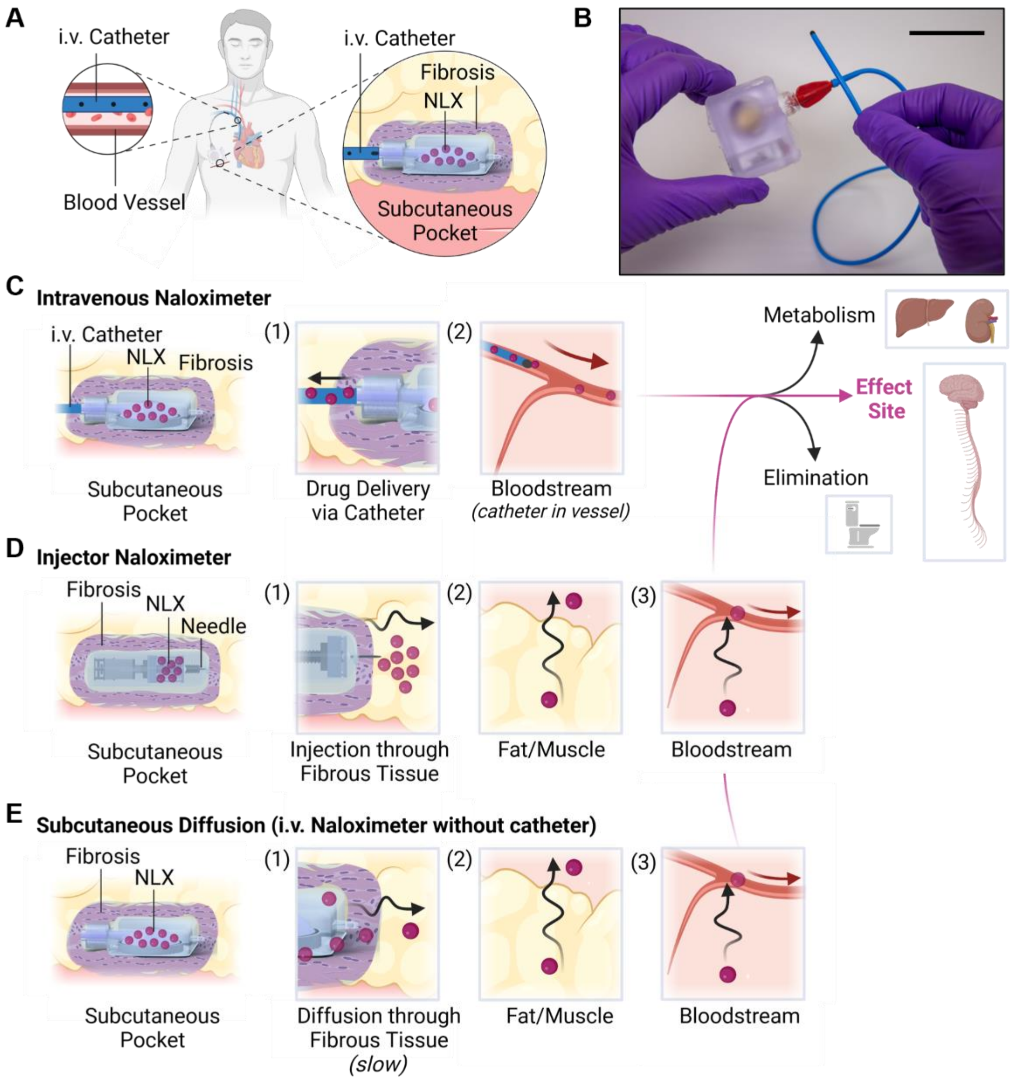

Fig. S21. Illustrations of implantation scheme and drug delivery route for Naloximeters used in porcine models. (A) Simple illustration of the implantation strategy for an intravenous (i.v.) Naloximeter device in a human. **(B)** Optical image of intravenous Naloximeter device with Groshong i.v. catheter attached, scale bar is 5 cm. Illustration of drug delivery route for **(C)** intravenous and **(D)** injector Naloximeter devices highlighting the path of naloxone (NLX) from device to bloodstream and effect site (central nervous system). **(E)** Illustration of subcutaneous diffusive drug delivery from electrolytic pumping Naloximeter without an i.v. catheter, drug slowly diffuses through fibrous tissue surrounding the device before moving through surrounding fat and muscle to the bloodstream.

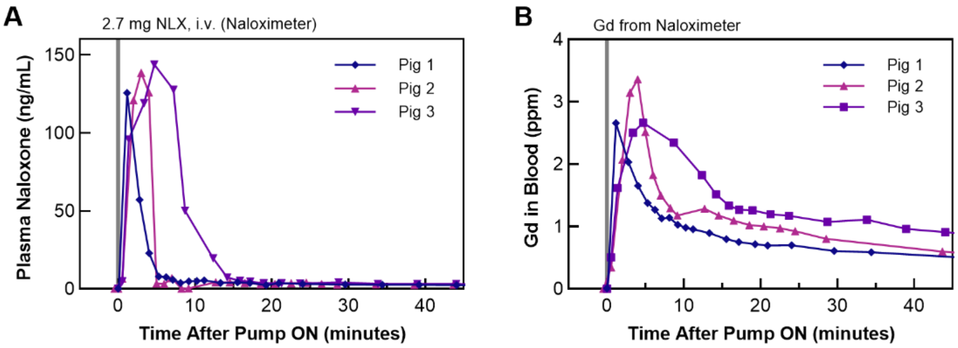

Fig. S22. Gadolinium (Gd) and Naloxone pharmacokinetics in porcine models with intravenous Naloximeter devices. (A) Naloxone and **(B)** Gd measured in plasma and blood, respectively, after delivery of 3 mL of drug solution from Naloximeter device. $N = 3$ animals, the same blood draw was split into samples for NLX and Gd quantification to facilitate direct comparison between the methods.

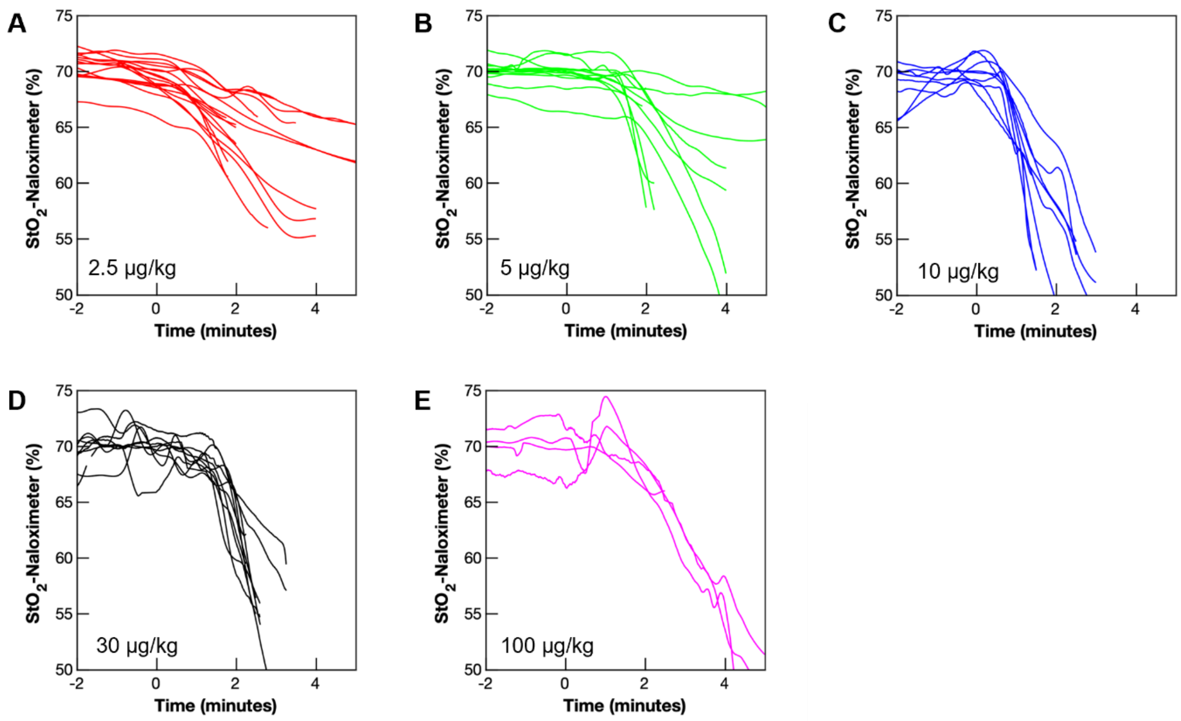

Fig. S23. Tissue oxygenation (StO_2) recorded with Naloximeters in porcine models at different fentanyl doses. (A) Fentanyl dose of 2.5 μg/kg, N = 6 subjects and n = 17 devices. **(B)** Fentanyl dose of 5 μg/kg, N = 4 subjects and n = 12 devices. **(C)** Fentanyl dose of 10 μg/kg, N = 9 subjects and n = 9 devices. **(D)** Fentanyl dose of 30 μg/kg, N = 6 subjects and n = 10 devices. **(E)** Fentanyl dose of 100 μg/kg, N = 4 subjects and n = 4 devices. Fentanyl was administered at time = 0. Overall N = 29 subjects, n = 52 devices.

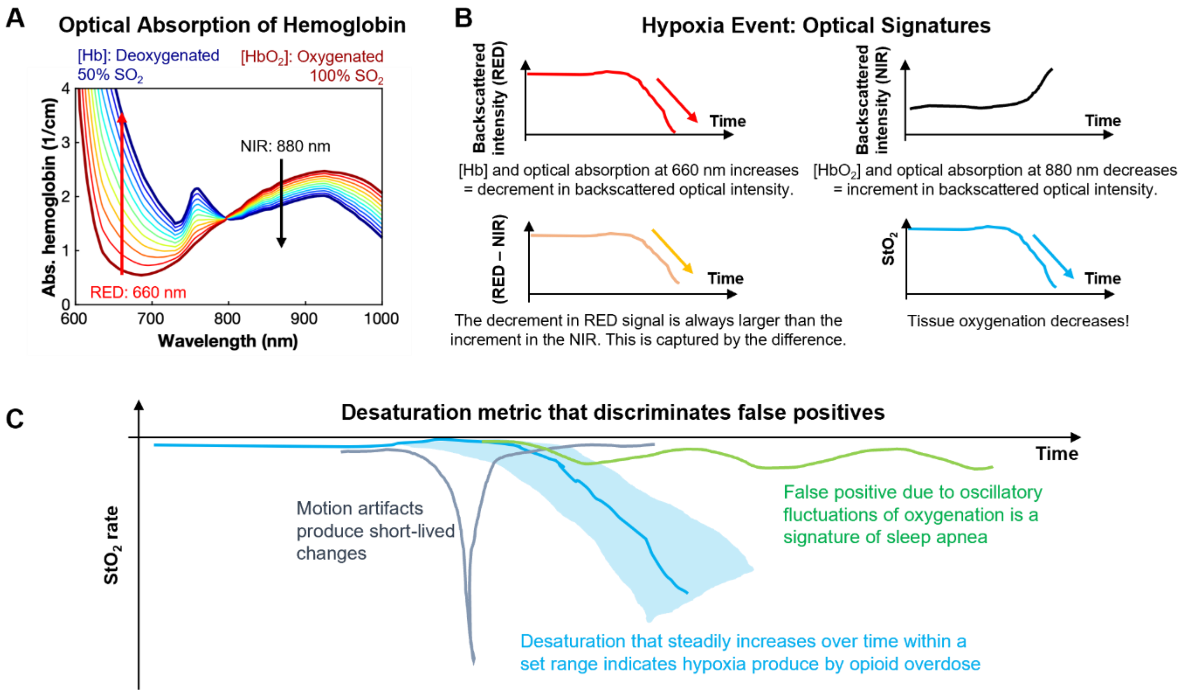

Fig. S24. Physiological dynamics during hypoxia produce measurable signatures that contrast with false positives produced by motion artifacts and sleep apneas. (A) Optical absorption of hemoglobin depends on the concentration of its oxygenated (HbO_2) and deoxygenated (Hb) states. Based on data from ref *(50).* Graph shows the change in the optical absorption as hemoglobin oxygenation changes from 100% SO_2 (dark red) to 50% SO_2 (dark blue). Differential spectroscopy, at the red = 660 nm ($\boldsymbol{\lambda_1}$) and near-infrared (NIR) = 880 nm ($\boldsymbol{\lambda_2}$) wavelengths, is used to calculate the tissue oxygenation ($\boldsymbol{StO_2}$). These signals capture the physiological dynamics. **(B)** Hypoxia is accompanied by clear changes in the optical signal and the calculated StO_2. These data produce a set of variables to detect desaturation. **(C)** Typical confounding events follow from motion artifacts and behaviors associated with sleep apnea. Motion artifacts can lead to false positives that are typically eliminated based on criteria associated with the rate of change of the optical signal. Episodes of desaturation due to sleep apnea can be eliminated by their characteristic oscillatory patterns and desaturation rate.

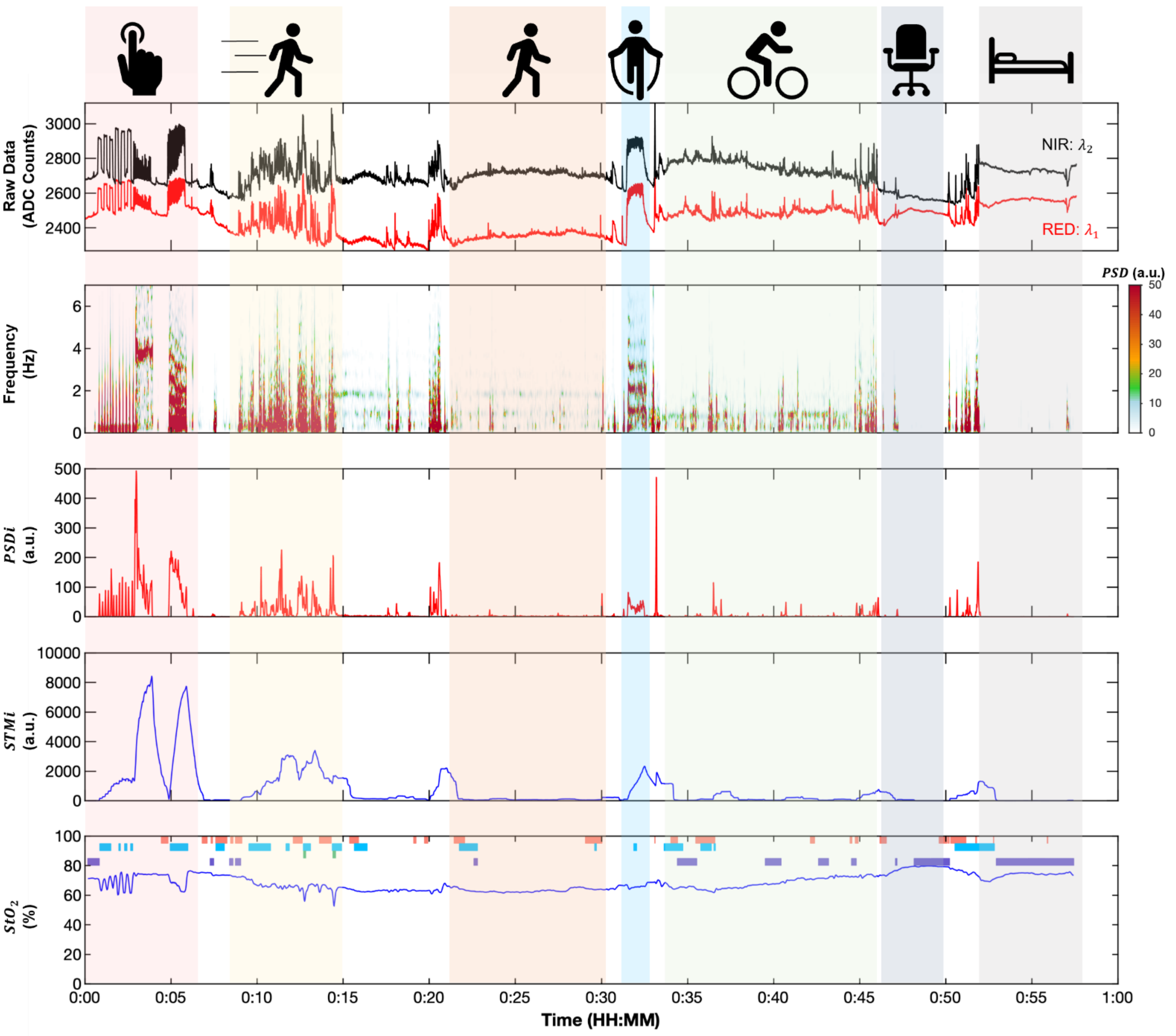

Fig. S25. Data from cutaneous optical sensors in humans associated with different types of physical activity. Data recorded with dual-wavelength optical sensor mounted on the skin in the arm of a human subject volunteer performing a series of movements. Movement sequence: Applying pressure to the skin (red), running (yellow), walking (orange), aerobic jumping (blue), cycling (green), resting while seated (blue-gray), resting while supinated (gray). Plots from top-down: red and near-infrared (NIR) optical signals; spectrogram; power spectral density index ($\boldsymbol{PSDi}$) in the 1-5 Hz frequency band; short-term motion index ($\boldsymbol{STMi}$, 60 s) and calculated tissue oxygenation ($\boldsymbol{StO_2}$).

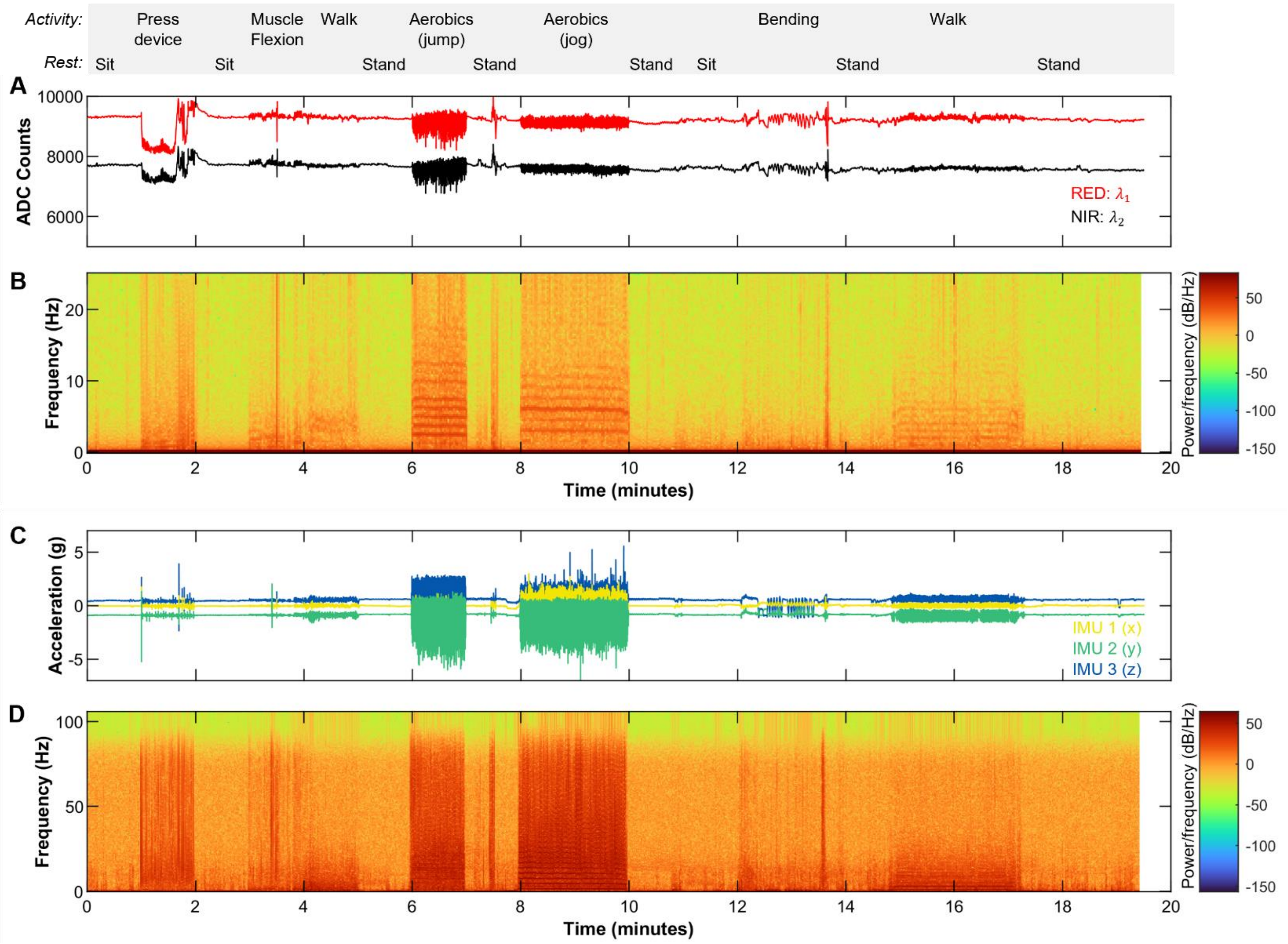

Fig. S26. Motion correlation between Naloximeter optical sensor and accelerometry sensor during simultaneous recording of activities in human. (A) Raw optical signals (λ_1 and λ_2) from cutaneous Naloximeter device and **(B)** corresponding spectrogram. Spectrogram inputs were window size = 200, window overlap = 190, and nfft = 200 samples for a sampling rate of 50 Hz**.** **(C)** Raw accelerometry signals for a cutaneous triaxial inertial sensor with axes denoted by numerals 1, 2, and 3, and **(D)** corresponding spectrogram. Spectrogram inputs were window size = 500, window overlap = 256, and nfft = 500 samples for a sampling rate of 211.23 Hz**.**

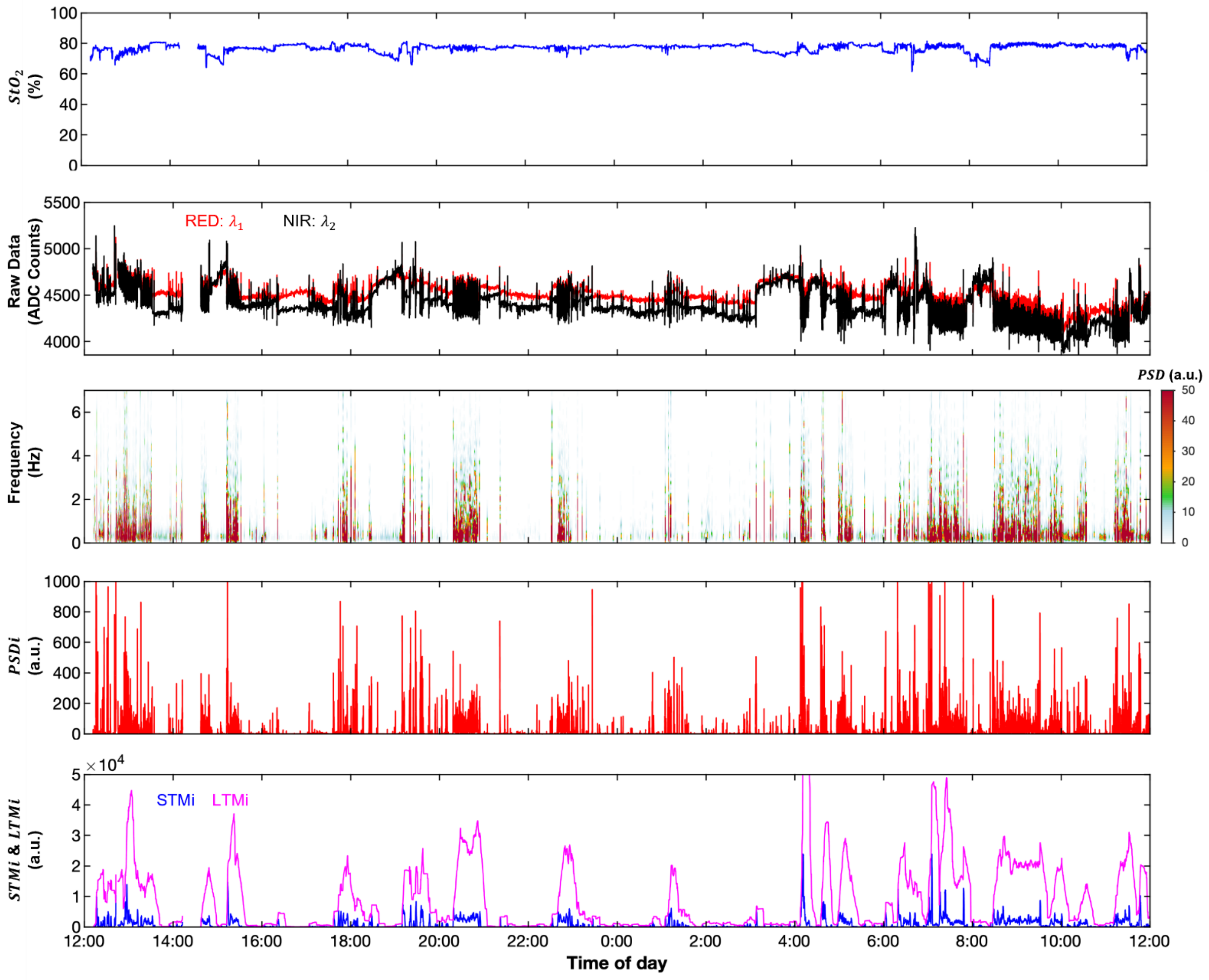

Fig. S27. Cumulative power spectral density index for defining episodes of physical activity. Data recorded with dual-wavelength optical sensor in a freely moving porcine model over 24 hours. From top-down: calculated tissue oxygenation ($\boldsymbol{StO_2}$); red and near-infrared (NIR) raw optical signals; spectrogram; power spectral density index ($\boldsymbol{PSDi}$) in the 1-5 Hz frequency band; short-term motion index ($\boldsymbol{STMi}$, 60 s) and long-term motion index ($\boldsymbol{LTMi}$, 10 min). Episodes of high motion index, apparent in the raw data, corresponds with the episode of increased $\boldsymbol{LTMi}$, power spectral density and $\boldsymbol{PSDi}$.

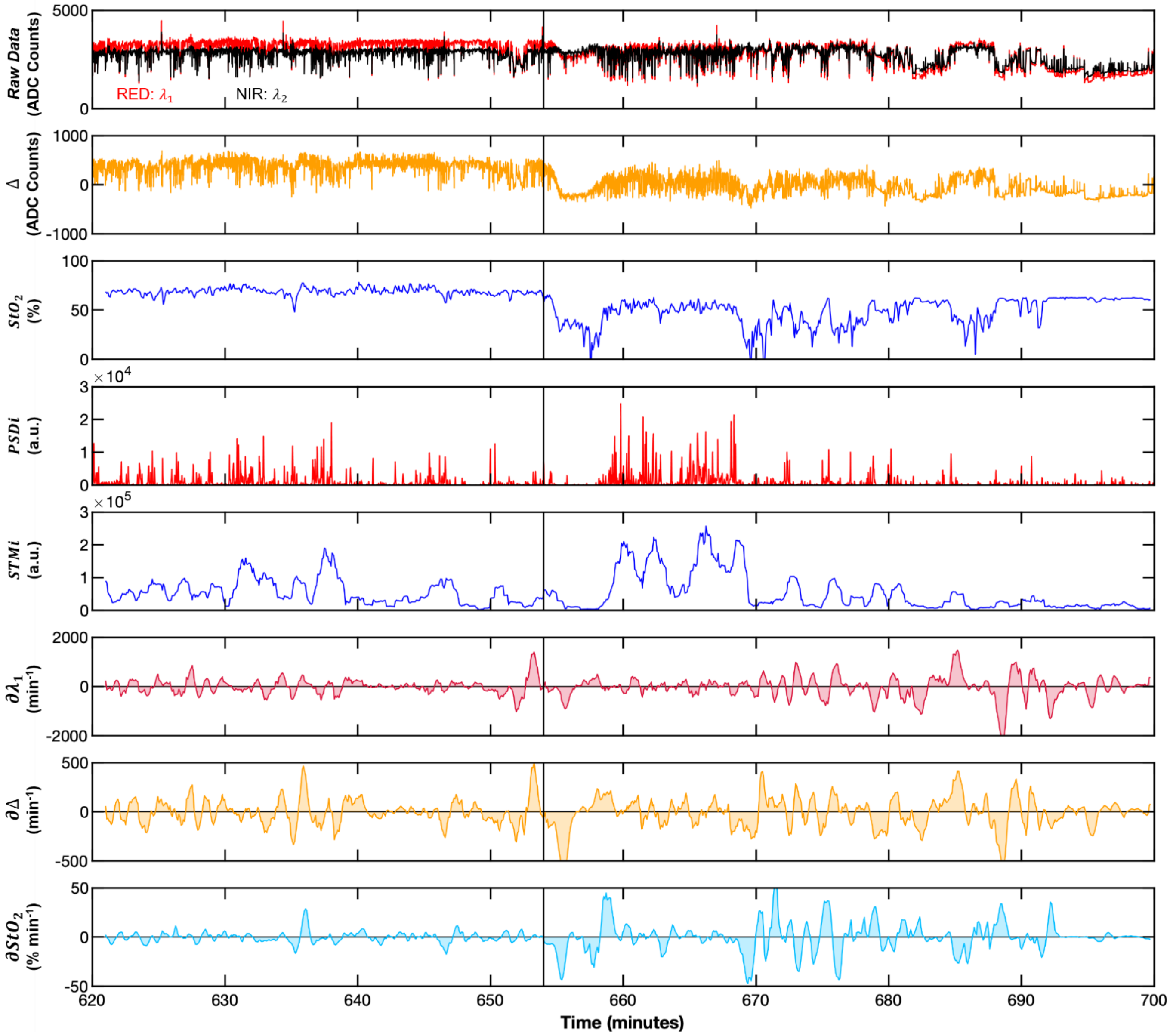

Fig. S28. Optical sensor data, and its multivariable derivatives, during fentanyl overdose in a freely moving pig. From top-down: raw data of the red (RED, $\boldsymbol{\lambda_1}$) and near-infrared (NIR, $\boldsymbol{\lambda_2}$) optical signals; difference between these signals ($\Delta = \boldsymbol{\lambda_1} - \boldsymbol{\lambda_2}$); calculated tissue oxygenation ($\boldsymbol{StO_2}$); power spectral density index ($\boldsymbol{PSDi}$) in the 1–5 Hz frequency band; short-term motion index ($\boldsymbol{STMi}$); rate of change ($\boldsymbol{\partial}$) of red ($\boldsymbol{\partial\lambda_1}$); difference ($\boldsymbol{\partial}\Delta$); and tissue oxygenation ($\boldsymbol{\partial StO_2}$). The time of fentanyl administration is indicated with a vertical line.

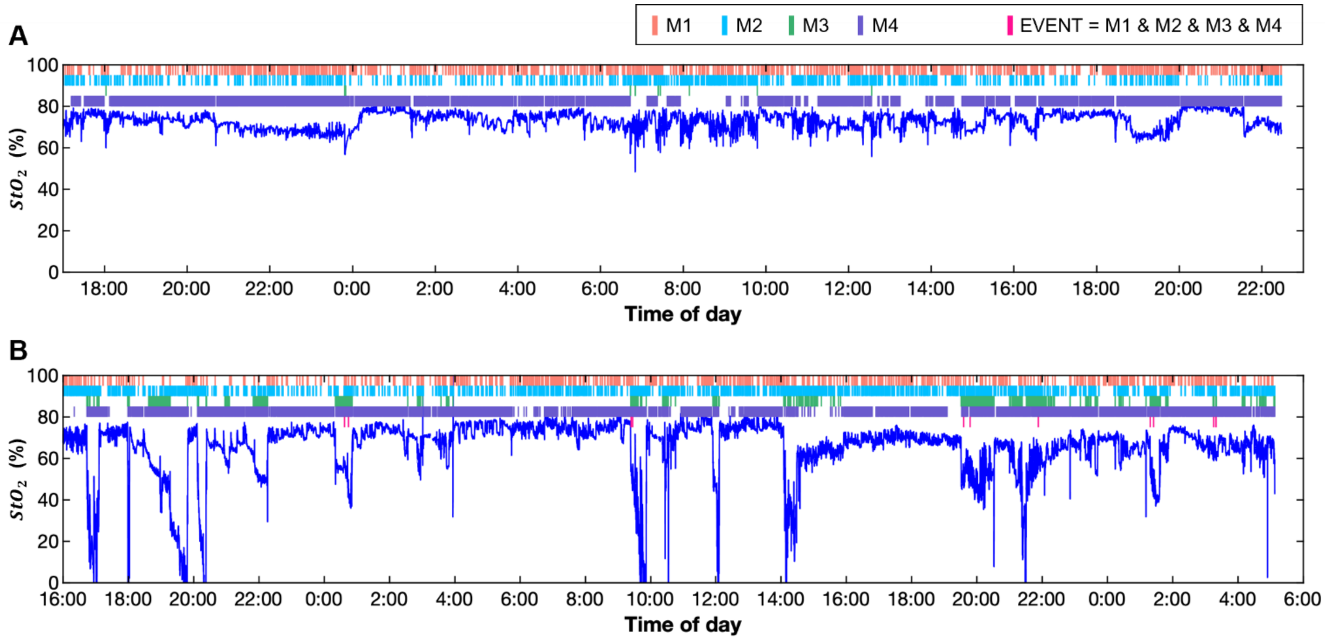

Fig. S29. Extended recordings of oxygenation (StO_2) with Naloximeters in freely moving pigs. (A, B) Colored bars represent instances where the logical metrics (M1 – M4) are true. EVENTS (M1 & M2 & M3 & M4) are registered in (B) but without satisfying the sequential condition to quality for an overdose. *N* = 2 animals with Naloximeters implanted in **(A)** the neck (jugular approach) for 15 days and **(B)** the flank (mammary approach) for 32 days.

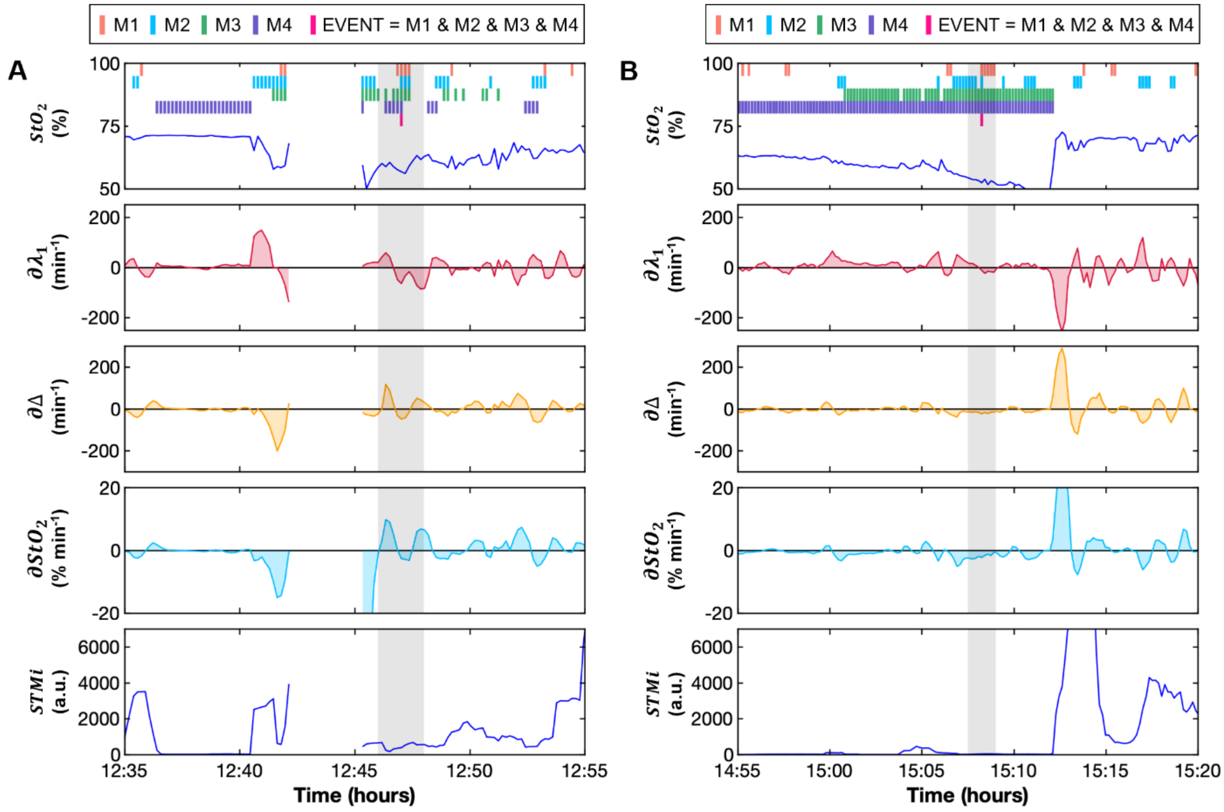

Fig. S30. Metrics associated with algorithm validation over 24 hours at selected times where an event was detected in data from Fig. 3N. (A, B) From top-down: calculated tissue oxygenation ($\boldsymbol{StO_2}$) showing when the logical metric conditions (M1, M2, M3, and M4) are true; rate of change ($\boldsymbol{\partial}$) of red ($\boldsymbol{\partial\lambda_1}$); difference ($\boldsymbol{\partial}\Delta$); and tissue oxygenation ($\boldsymbol{\partial StO_2}$); short-term motion index ($\boldsymbol{STMi}$). The instances of EVENTs (M1 & M2 & M3 & M4) are isolated and did not produce a WARNING (three consecutive occurrences). Each column represents a distinct episode.

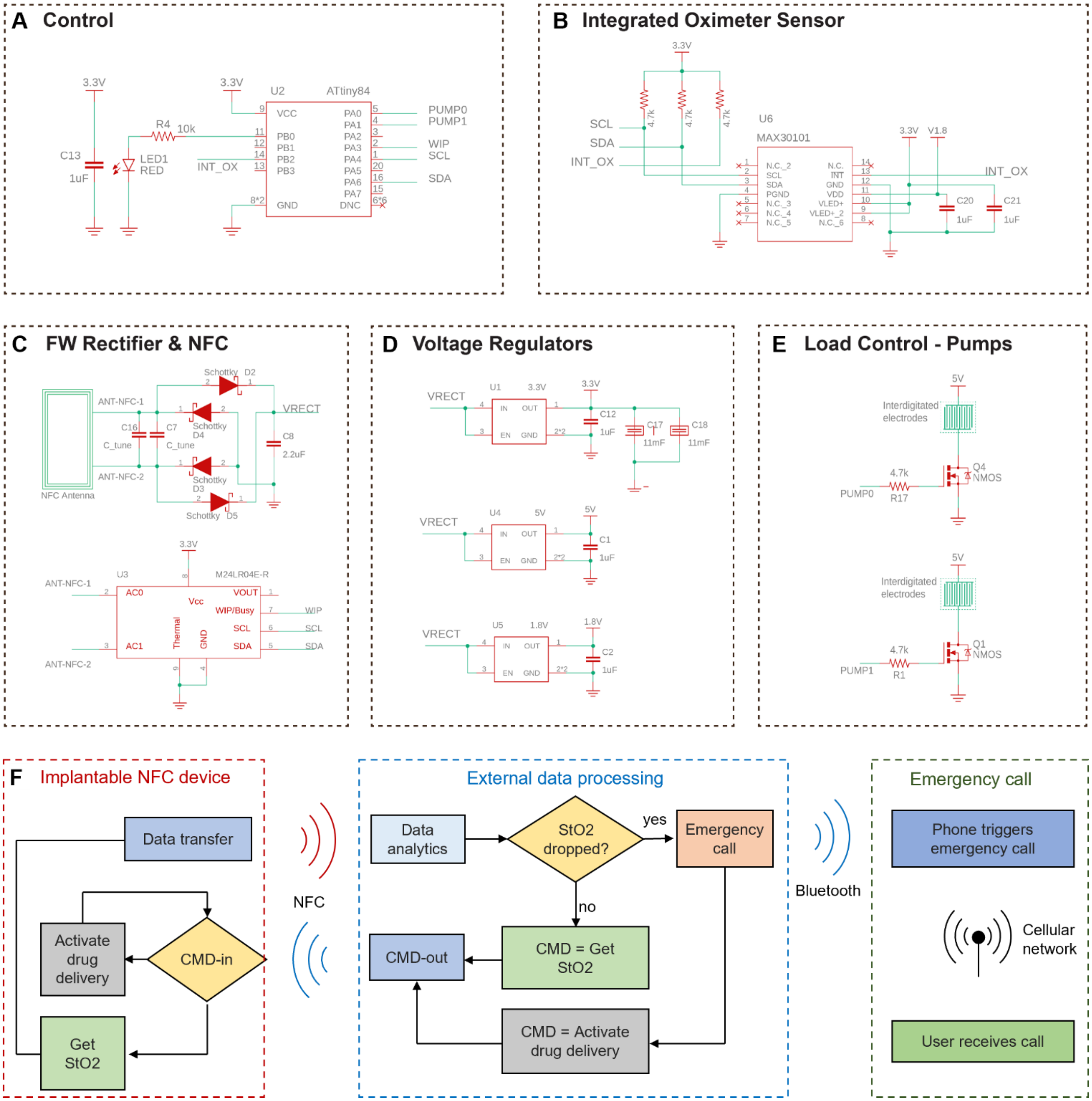

Fig. S31. Electronic circuit and functional block diagrams for the battery-free, NFC Naloximeter for rodents. (A) The ATTiny84 microcontroller controls the operation of the device and the manages the wireless communication. **(B)** The integrated optical sensor MAX30101 connects with the MCU using I^2C communication. **(C)** RF front end containing a full-wave rectifier and an RF random access memory used for bidirectional NFC communication. **(D)** A voltage regulator supplies 3.3 V to supercapacitor bank (top). A 5 V regulator provides voltage to the electrolytic pumps (center) and a 1.8 V regulator supplies voltage to the dual-wavelength

optical sensor (bottom). **(E)** Pump control system with MOSFETs and interdigitated gold electroplated electrodes. Each pump is controlled independently. **(F)** Functional block diagram of the NFC Naloximeter that demonstrates the close-loop implementation in rodents. From left to right: Implanted NFC device; external data processing unit implemented in MATLAB; emergency call management system.

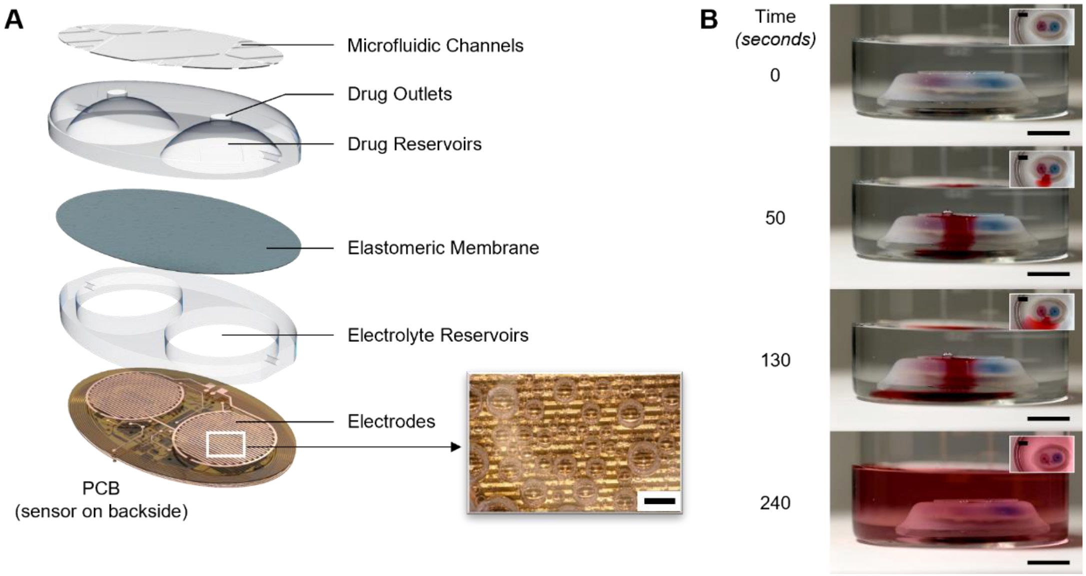

Fig. S32. Battery-free, NFC Naloximeter for rodents. (A) Exploded view schematic illustration of the device with inset image of electrolytic gas generation by interdigitated electrodes. Scale bar, 1 mm. **(B)** Photographs that show drug delivery, using food dye as drug proxy for purposes of visualization. Scale bars, 1 cm.

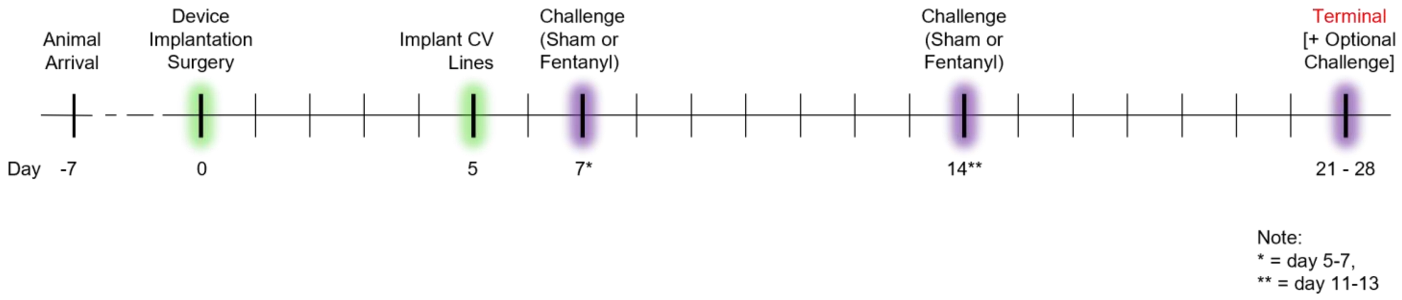

Fig. S33. General timeline for large animal survival studies.

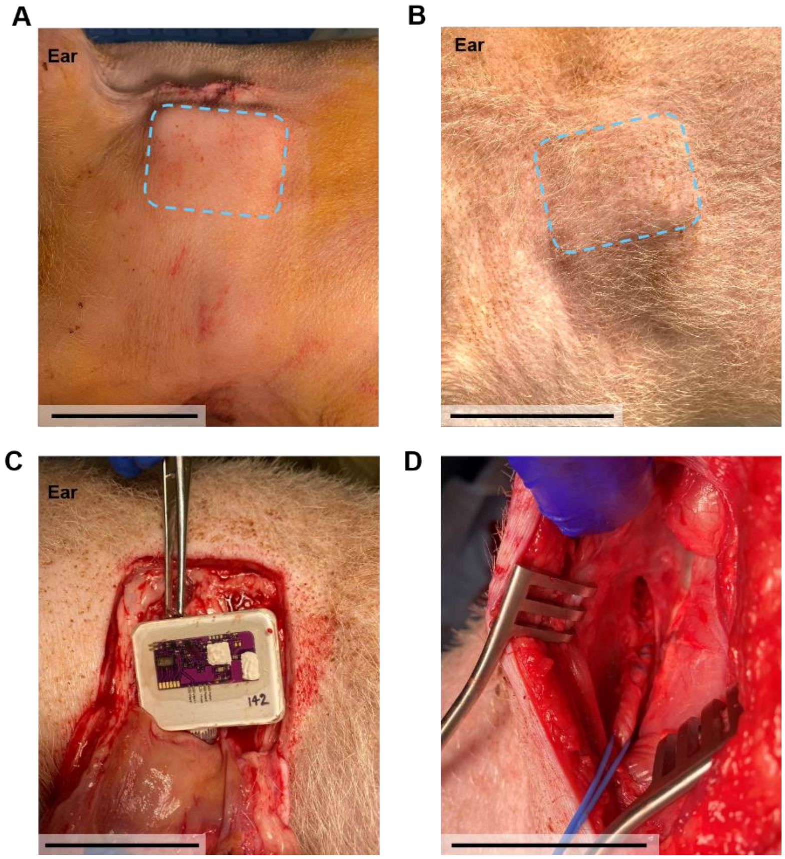

Fig. S34. Healing progression for an intravenous Naloximeter implanted in the jugular vein of a pig model. Photographs near the area **(A)** immediately after implantation and **(B)** on day 44 post-implantation. Dashed boxes denote the device outline. Photographs of the **(C)** implanted Naloximeter and **(D)** catheter intact in the jugular vein at day 44 post-implantation. Scale bars, 5 cm.

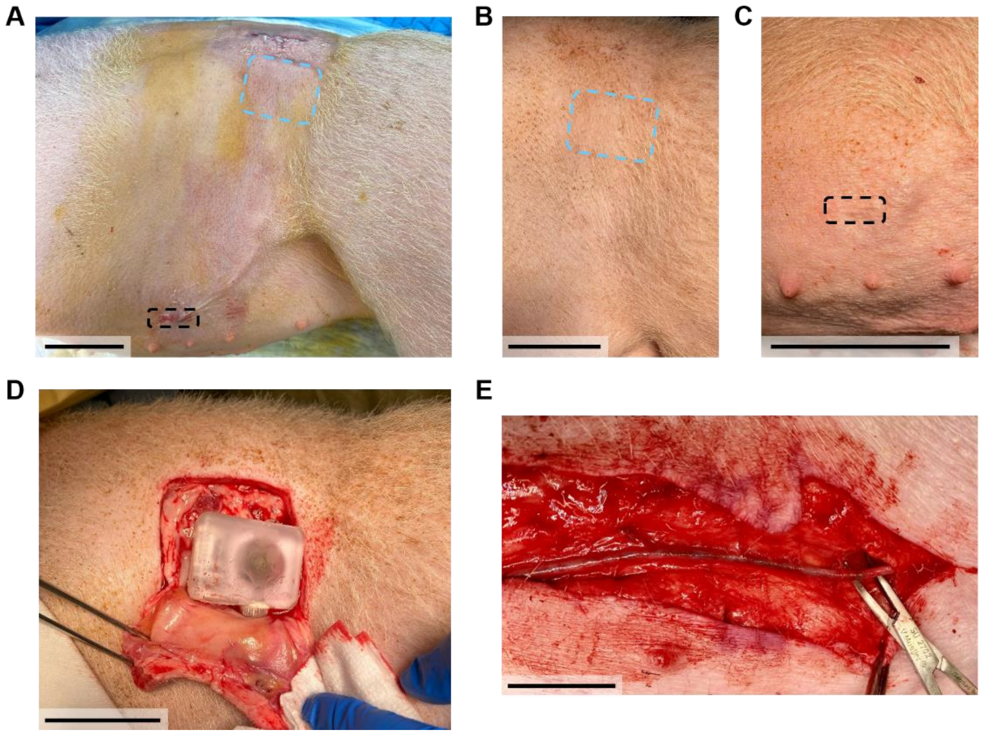

Fig. S35. Healing progression for an intravenous Naloximeter implanted in the mammary vein. Photographs near the area **(A)** immediately after implantation and **(B, C)** on day 44 post-implantation at the device and catheter locations, respectively. Dashed blue boxes denote the device outline. The dashed black boxes denote the vascular access incision. Photographs of the **(D)** implanted device and **(E)** catheter intact in the mammary vein at day 44 post-implantation. Scale bars, 5 cm.

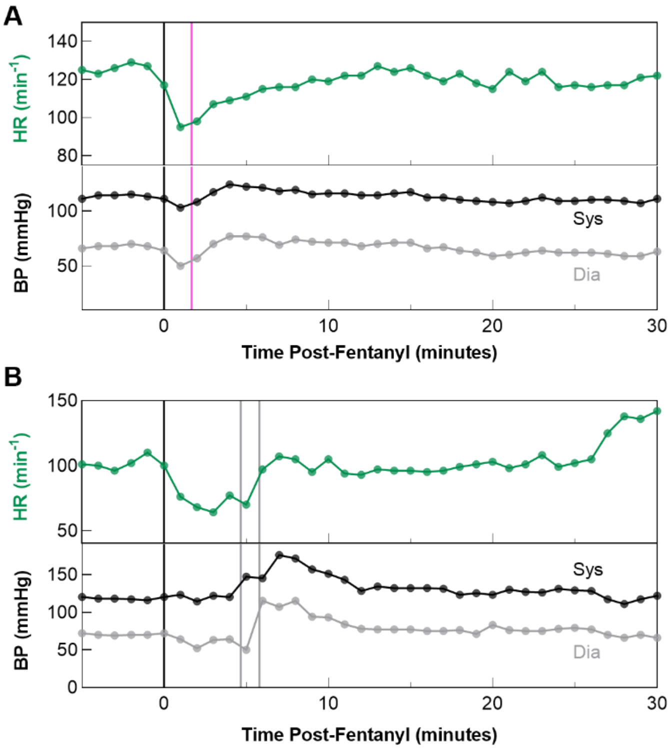

Fig. S36. Additional vital signs from (A) closed-loop rescue with Naloximeter and (B) the control case without Naloximeter in anesthetized pig model at 2.5 µg/kg fentanyl dose. Top: Heart rate from electrocardiogram (ECG), bottom: invasive blood pressure. Black line denotes time of fentanyl (i.v.) dose, pink line denotes time of closed-loop NLX (i.v.), gray line denotes time of manual rescue NLX (i.v.). Data in A, B correspond to overdoses in Fig. 4E, 4F, respectively.

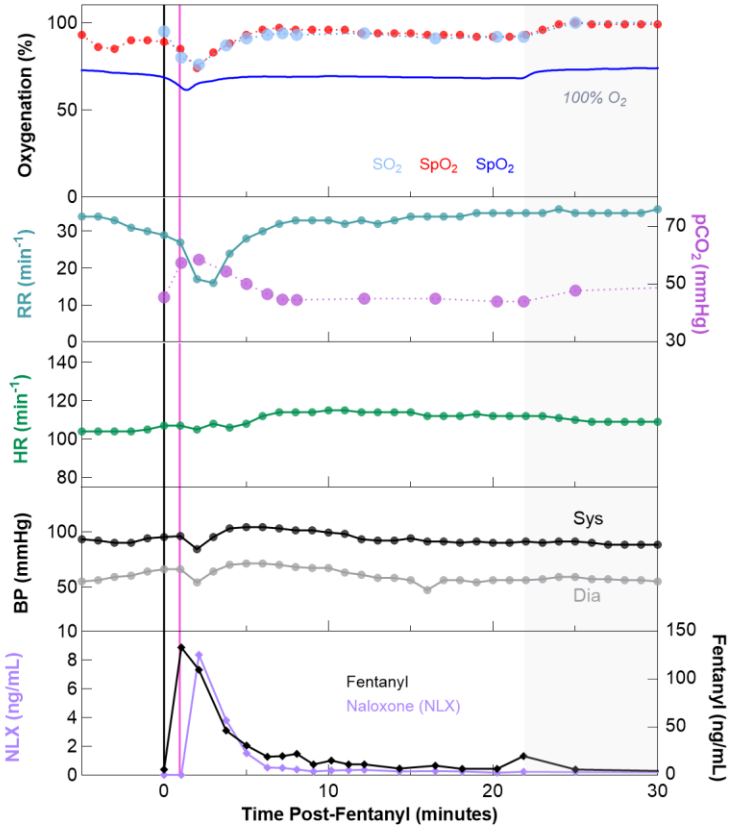

Fig. S37. Closed-loop rescue demonstration with intravenous Naloximeter in anesthetized pig model at 2.5 µg/kg fentanyl dose. From top-down: Oxygenation (SO_2: Arterial Blood Gas, SpO_2: commercial pulse oximeter, StO_2: Naloximeter), respiratory vitals, heart rate from electrocardiogram (ECG), invasive blood pressure, and pharmacokinetics of naloxone (NLX) and fentanyl. Black line denotes time of fentanyl (i.v.) dose, pink line denotes time of closed-loop NLX.

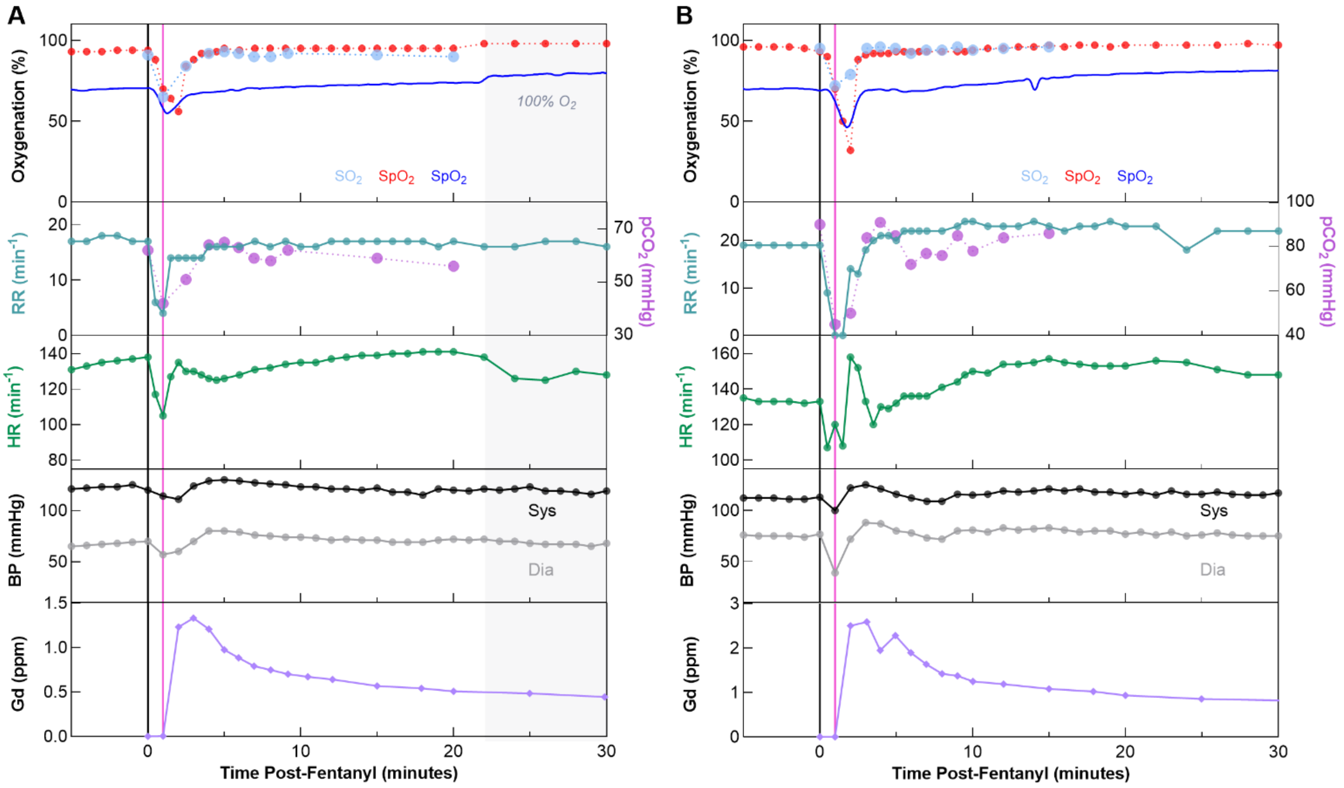

Fig. S38. Closed-loop rescue demonstrations with intravenous Naloximeter in anesthetized pig model at 5 µg/kg fentanyl dose. (A, B) From top-down: Oxygenation (SO_2: Arterial Blood Gas, SpO_2: commercial pulse oximeter, StO_2: Naloximeter), respiratory vitals, heart rate from electrocardiogram (ECG), invasive blood pressure, and gadolinium pharmacokinetics. Black line denotes time of fentanyl (i.v.) dose, pink line denotes time of closed-loop NLX (i.v.).

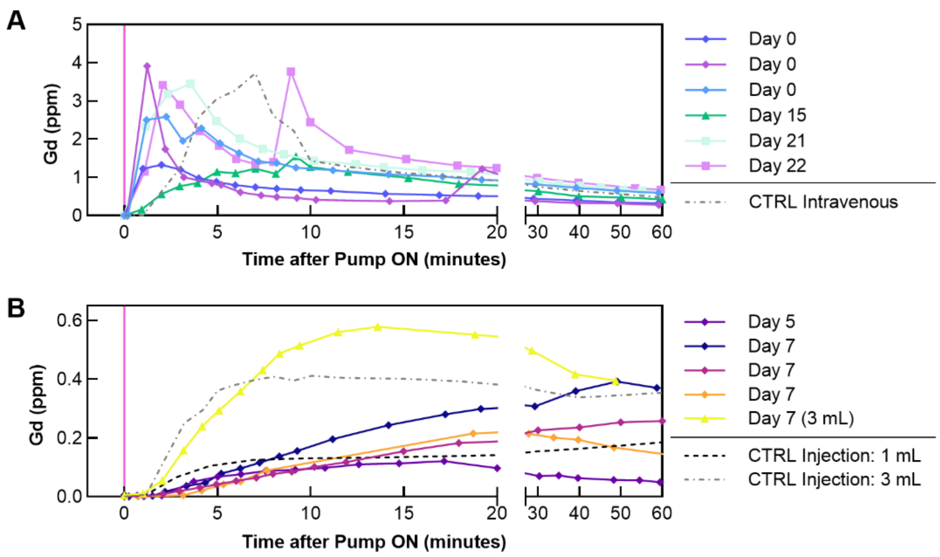

Fig. S39. Pharmacokinetics of NLX proxy, Gadolinium (Gd), when delivered by the intravenous Naloximeter after various implantation durations. (A) Gadolinium pharmacokinetics when delivered intravenously by the Naloximeter (mammary and jugular vein) between 0 and 22 days after implantation, or a medical infusion pump in the auricular vein (CTRL Intravenous). Dose is 3 mL. **(B)** Gadolinium pharmacokinetics when delivered subcutaneously by the Naloximeter (without a catheter) between 5 and 7 days after implantation, or by manual subcutaneous injection in tissue surrounding healed devices at 21 and 71 days after implantation (CTRL Injection). The subcutaneous dose is 1.5 mL unless otherwise specified.

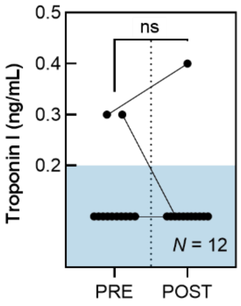

Fig. S40. Blood biomarker for cardiac function before (PRE) and after (POST) intravenous Naloximeter drug delivery. Troponin I quantified from blood serum with a veterinary Troponin I test (IDEXX Laboratories). Values above 0.2 ng/mL are considered elevated, values less than this are plotted within threshold box (lower reporting limit 0.2 ng/mL). Difference is not significant between groups (paired t-test, P = 0.674), though one case showed elevated Troponin levels in both PRE and POST samples.

State	Average current (mA)	Average power (mW)
Advertising	0.175	0.664
Connection	1.017	3.863
Connected (idle)	0.200	0.758
Connected + Sending Status Updates	0.231	0.877
Connected + Optical Sensor ON	1.461	5.553
Connected + Optical Sensor Intermittent (10% d.c., T = 10 s)	0.326	1.239
Connected + Optical Sensor Intermittent (50% d.c., T = 10 s)	0.830	3.154
Connected + Optical Sensor Intermittent (20% d.c., T = 5 s)	0.452	1.718

Table S1. Summary of power consumption of the Naloximeter for large animals during different operational states. Average current and power consumption calculated over a 5 s window.

Fentanyl Dose (μg/kg)	Number of Overdose Experiments (N)	Number of Devices (n)
100	4	4
30	6	10
10	9	9
5	4	12
2.5	6	17

Table S2. Summary of subjects and devices used in characterization of the desaturation response to fentanyl in porcine models.

Movie S1.

Demonstration of drug delivery from intravenous Naloximeter device. Pink dye (Rhodamine B) represents NLX solution, clear container of water represents vasculature. Video speed: 1× and 8× (noted in video).

Movie S2.

Demonstration of drug delivery with injector Naloximeter device, showing dual-injection. Blue solution indicates NLX solution and yellow solution represents biofluid in the body. Speed: 1×. Scale bar, 1 cm.

REFERENCES AND NOTES

1. J. Strang, N. D. Volkow, L. Degenhardt, M. Hickman, K. Johnson, G. F. Koob, B. D. L. Marshall, M. Tyndall, S. L. Walsh, Opioid use disorder. *Nat. Rev. Dis. Primer* **6**, 1–28 (2020).

2. C. P. France, G. P. Ahern, S. Averick, A. Disney, H. A. Enright, B. Esmaeli-Azad, A. Federico, L. R. Gerak, S. M. Husbands, B. Kolber, E. Y. Lau, V. Lao, D. R. Maguire, M. A. Malfatti, G. Martinez, B. P. Mayer, M. Pravetoni, N. Sahibzada, P. Skolnick, E. Y. Snyder, N. Tomycz, C. A. Valdez, J. Zapf, Countermeasures for preventing and treating opioid overdose. *Clin. Pharmacol. Ther*. **109**, 578–590 (2021).

3. L. Degenhardt, J. Grebely, J. Stone, M. Hickman, P. Vickerman, B. D. L. Marshall, J. Bruneau, F. L. Altice, G. Henderson, A. Rahimi-Movaghar, S. Larney, Global patterns of opioid use and dependence: Harms to populations, interventions, and future action. *The Lancet* **394**, 1560–1579 (2019).

4. C. Blanco, N. D. Volkow, Management of opioid use disorder in the USA: Present status and future directions. *The Lancet* **393**, 1760–1772 (2019).

5. N. D. Volkow, C. Blanco, The changing opioid crisis: Development, challenges and opportunities. *Mol. Psychiatry* **26**, 218–233 (2021).

6. J. O'Donnell, R. M. Gladden, C. L. Mattson, C. T. Hunter, N. L. Davis, Vital signs: Characteristics of drug overdose deaths involving opioids and stimulants - 24 States and the District of Columbia, January-June 2019. *MMWR Morb. Mortal. Wkly Rep.* **69**, 1189–1197 (2020).

7. F. B. Ahmad, J. Cisewksi, L. M. Rossen, P. Sutton, "Provisional Drug Overdose Death Counts," (National Center for Health Statistics, 2024); https://cdc.gov/nchs/nvss/vsrr/drug-overdose-data.htm.

8. Mortality Multiple Cause, National Center for Health Statistics: National Vital Statistics System (2022); https://cdc.gov/nchs/data_access/vitalstatsonline.htm.

9. C. M. Jones, F. Bekheet, J. N. Park, G. C. Alexander, The evolving overdose epidemic: Synthetic opioids and rising stimulant-related harms. *Epidemiol. Rev.* **42**, 154–166 (2020).

10. M. R. Spencer, M. F. Garnett, A. M. Miniño, "Drug Overdose Deaths in the United States, 2002–2022," (No. 491, National Center for Health Statistics, Hyattsville, MD, 2024); https://dx.doi.org/10.15620/cdc:135849.

11. L. Degenhardt, D. Randall, W. Hall, M. Law, T. Butler, L. Burns, Mortality among clients of a state-wide opioid pharmacotherapy program over 20 years: Risk factors and lives saved. *Drug Alcohol Depend.* **105**, 9–15 (2009).

12. E. Ødegård, E. J. Amundsen, K. B. Kielland, R. Kristoffersen, The contribution of imprisonment and release to fatal overdose among a cohort of Norwegian drug abusers. *Addict. Res. Theory* **18**, 51–58 (2010).

13. D. Lewer, B. Eastwood, M. White, T. D. Brothers, M. McCusker, C. Copeland, M. Farrell, I. Petersen, Fatal opioid overdoses during and shortly after hospital admissions in England: A case-crossover study. *PLoS Med.* **18**, e1003759 (2021).

14. E. Ravndal, E. J. Amundsen, Mortality among drug users after discharge from inpatient treatment: An 8-year prospective study. *Drug Alcohol Depend.* **108**, 65–69 (2010).

15. B. P. Smyth, J. Barry, E. Keenan, K. Ducray, Lapse and relapse following inpatient treatment of opiate dependence. *Ir. Med. J.* **103**, 176–179 (2010).

16. S. G. Weiner, O. Baker, D. Bernson, J. D. Schuur, One-year mortality of patients after emergency department treatment for nonfatal opioid overdose. *Ann. Emerg. Med.* **75**, 13–17 (2020).

17. S. A. Kitchen, D. McCormack, D. Werb, A. Caudarella, D. Martins, F. I. Matheson, T. Gomes, Trends and outcomes of serious complications associated with non-fatal opioid overdoses in Ontario, Canada. *Drug Alcohol Depend.* **225**, 108830 (2021).

18. K. T. S. Pattinson, Opioids and the control of respiration. *Br. J. Anaesth.* **100**, 747–758 (2008).

19. E. W. Boyer, Management of opioid analgesic overdose. *N. Engl. J. Med.* **367**, 146–155 (2012).

20. E. Y. Schiller, A. Goyal, O. J. Mechanic, "Opioid Overdose" in *StatPearls* (StatPearls Publishing, 2024); http://ncbi.nlm.nih.gov/books/NBK470415/).

21. B. Palkovic, V. Marchenko, E. J. Zuperku, E. A. E. Stuth, A. G. Stucke, Multi-level regulation of opioid-induced respiratory depression. *Phys. Ther.* **35**, 391–404 (2020).

22. R. Rzasa Lynn, J. Galinkin, Naloxone dosage for opioid reversal: Current evidence and clinical implications. *Ther. Adv. Drug Saf.* **9**, 63–88 (2018).

23. R. Abdelal, A. Raja Banerjee, S. Carlberg-Racich, N. Darwaza, D. Ito, J. Shoaff, J. Epstein, Real-world study of multiple naloxone administration for opioid overdose reversal among bystanders. *Harm Reduct. J.* **19**, 49 (2022).

24. T. C. Green, R. Heimer, L. E. Grau, Distinguishing signs of opioid overdose and indication for naloxone: An evaluation of six overdose training and naloxone distribution programs in the United States. *Addiction* **103**, 979–989 (2008).

25. A. K. Winiker, K. E. Tobin, R. E. Gicquelais, J. Owczarzak, C. Latkin, "When you're getting high… You just don't want to be around anybody." A qualitative exploration of reasons for injecting alone: Perspectives from young people who inject drugs. *Subst. Use Misuse* **55**, 2079–2086 (2020).

26. T. Fleming, J. Boyd, M. Gagnon, T. Kerr, R. McNeil, Using drugs alone in single room occupancy housing: Understanding environmental drivers of overdose risk. *Int. J. Drug Policy* **128**, 104444 (2024).

27. T. W. Levengood, G. H. Yoon, M. J. Davoust, S. N. Ogden, B. D. L. Marshall, S. R. Cahill, A. R. Bazzi, supervised injection facilities as harm reduction: A systematic review. *Am. J. Prev. Med.* **61**, 738–749 (2021).

28. M. C. Kennedy, M. Karamouzian, T. Kerr, Public health and public order outcomes associated with supervised drug consumption facilities: A systematic review. *Curr. HIV/AIDS Rep.* **14**, 161–183 (2017).

29. V. W. L. Tsang, K. Papamihali, A. Crabtree, J. A. Buxton, Acceptability of technological solutions for overdose monitoring: Perspectives of people who use drugs. *Subst. Abuse* **42**, 284–293 (2021).

30. C. Goldfine, J. T. Lai, E. Lucey, M. Newcomb, S. Carreiro, Wearable and wireless mhealth technologies for substance use disorder. *Curr. Addict. Rep.* **7**, 291–300 (2020).

31. A. R. Lombardi, R. Arya, J. G. Rosen, E. Thompson, R. Welwean, J. Tardif, J. D. Rich, J. N. Park, Overdose detection technologies to reduce solitary overdose deaths: A literature review. *Int. J. Environ. Res. Public Health* **20**, 1230 (2023).

32. A. Oteo, H. Daneshvar, A. Baldacchino, C. Matheson, Overdose alert and response technologies: State-of-the-art review. *J. Med. Internet Res.* **25**, e40389 (2023).

33. W. Rioux, T. Marshall, S. M. Ghosh, Virtual overdose monitoring services and overdose prevention technologies: Opportunities, limitations, and future directions. *Int. J. Drug Policy* **119**, 104121 (2023).

34. R. Nandakumar, S. Gollakota, J. E. Sunshine, Opioid overdose detection using smartphones. *Sci. Transl. Med.* **11**, eaau8914 (2019).

35. Brave Sensors; https://brave.coop/overdose-detection-sensors.

36. Care Technology - Technology Enabled Care | How It Works – Carezapp; https://carezapp.com/how-it-works/.

37. B. Dhowan, J. Lim, M. D. MacLean, A. G. Berman, M. K. Kim, Q. Yang, J. Linnes, C. H. Lee, C. J. Goergen, H. Lee, Simple minimally-invasive automatic antidote delivery device (A2D2) towards closed-loop reversal of opioid overdose. *J. Control. Release* **306**, 130–137 (2019).

38. J. Chan, V. Iyer, A. Wang, A. Lyness, P. Kooner, J. Sunshine, S. Gollakota, Closed-loop wearable naloxone injector system. *Sci. Rep.* **11**, 22663 (2021).

39. M. S. Imtiaz, C. V. Bandoian, T. J. Santoro, Hypoxia driven opioid targeted automated device for overdose rescue. *Sci. Rep.* **11**, 24513 (2021).

40. A. M. Roth, N. K. Tran, B. Cocchiaro, A. K. Mitchell, D. G. Schwartz, D. J. Hensel, J. Ataiants, J. Brenner, I. Yahav, S. E. Lankenau, Wearable biosensors have the potential to monitor physiological changes associated with opioid overdose among people who use drugs: A proof-of-concept study in a real-world setting. *Drug Alcohol Depend.* **229**, 109138 (2021).

41. PneumoWave - Real-time respiratory depression monitoring, *PneumoWave* (2024); https://pneumowave.com/.

42. S. Carreiro, D. Smelson, M. Ranney, K. J. Horvath, R. W. Picard, E. D. Boudreaux, R. Hayes, E. W. Boyer, Real-time mobile detection of drug use with wearable biosensors: A pilot study. *J. Med. Toxicol.* **11**, 73–79 (2015).

43. S. Carreiro, K. Wittbold, P. Indic, H. Fang, J. Zhang, E. W. Boyer, Wearable biosensors to detect physiologic change during opioid use. *J. Med. Toxicol.* **12**, 255–262 (2016).

44. A. Sakudo, Near-infrared spectroscopy for medical applications: Current status and future perspectives. *Clin. Chim. Acta* **455**, 181–188 (2016).

45. J. M. Anderson, "Inflammation, wound healing, and the foreign-body response," in *Biomaterials Science* (Elsevier, 2013) , pp. 503–512; https://linkinghub.elsevier.com/retrieve/pii/B9780080877808000449.

46. J. R. Gill, P. T. Lin, L. Nelson, Erratum to: Reliability of postmortem fentanyl concentrations in determining the cause of death. *J. Med. Toxicol.* **11**, 382–382 (2015).

47. Facts about Fentanyl; https://dea.gov/resources/facts-about-fentanyl.

48. "Fentanyl Citrate Injection [package insert]," (Akorn Inc., Lake Forest, IL, USA, 2012).

49. K. M. Busl, D. M. Greer, Hypoxic-ischemic brain injury: Pathophysiology, neuropathology and mechanisms. *NeuroRehabilitation* **26**, 5–13 (2010).

50. S. Y. Lim, S. Woo, J. L. Miller, G. H. Skrepnek, E. D. Henry, P. N. Johnson, Prediction and comparison of fentanyl infusion pharmacokinetics in obese and nonobese children. *Pediatr. Crit. Care Med.* **20**, e556 (2019), e564.

51. A. Dahan, A. Yassen, H. Bijl, R. Romberg, E. Sarton, L. Teppema, E. Olofsen, M. Danhof, Comparison of the respiratory effects of intravenous buprenorphine and fentanyl in humans and rats. *Br. J. Anaesth.* **94**, 825–834 (2005).

52. E. Brandl, Z. Halford, M. D. Clark, C. Herndon, Pharmacogenomics in pain management: A review of relevant gene-drug associations and clinical considerations. *Ann. Pharmacother.* **55**, 1486–1501 (2021).

53. S. Wray, M. Cope, D. T. Delpy, J. S. Wyatt, E. O. R. Reynolds, Characterization of the near infrared absorption spectra of cytochrome aa3 and haemoglobin for the non-invasive monitoring of cerebral oxygenation. *Biochim. Biophys. Acta BBA - Bioenerg.* **933**, 184–192 (1988).

54. J. Hodson, The case for using implanted ports. *Br. J. Nurs.* **28**, S3–S10 (2019).

55. E. M. Walser, Venous access ports: Indications, implantation technique, follow-up, and complications. *Cardiovasc. Intervent. Radiol.* **35**, 751–764 (2012).

56. B. Al-Ghamdi, Subcutaneous implantable cardioverter defibrillators: An overview of implantation techniques and clinical outcomes. *Curr. Cardiol. Rev.* **15**, 38–48 (2019).

57. A. J. Greenspon, J. D. Patel, E. Lau, J. A. Ochoa, D. R. Frisch, R. T. Ho, B. B. Pavri, S. M. Kurtz, Trends in permanent pacemaker implantation in the United States from 1993 to 2009. *J. Am. Coll. Cardiol.* **60**, 1540–1545 (2012).

58. L. E. Groshong, R. J. Brawn, "Valved Two-Way Catheter," U.S. Patent 4 549 879, 29 Oct. 1985.

59. D. G. Greenhalgh, M. B. Lawless, B. B. Chew, W. A. Crone, M. E. Fein, T. L. Palmieri, Temperature threshold for burn injury: An oximeter safety study. *J. Burn Care Rehabil.* **25**, 411–415 (2004).

60. I. Oshina, J. Spigulis, Beer–Lambert law for optical tissue diagnostics: Current state of the art and the main limitations. *J. Biomed. Opt.* **26**, 100901 (2021).

61. F. F. Jöbsis, Noninvasive, infrared monitoring of cerebral and myocardial oxygen sufficiency and circulatory parameters. *Science* **198**, 1264–1267 (1977).

62. M. Lindkvist, G. Granåsen, C. Grönlund, Coherent derivation of equations for differential spectroscopy and spatially resolved spectroscopy: An undergraduate tutorial. *Spectrosc. Lett.* **46**, 243–249 (2013).

63. S. L. Jacques, Corrigendum: Optical properties of biological tissues: A review. *Phys. Med. Biol.* **58**, 5007–5008 (2013).

64. S. Hindel, A. Söhner, M. Maaß, W. Sauerwein, D. Möllmann, H. A. Baba, M. Kramer, L. Lüdemann, Validation of blood volume fraction quantification with 3D gradient echo dynamic contrast-enhanced magnetic resonance imaging in porcine skeletal muscle. *PLOS ONE* **12**, e0170841 (2017).

65. Y. S. Luo, Y. L. Luo, E. B. Ashford, R. R. Morin, W. J. White, T. F. Fisher, "Comparison of Catheter Lock Solutions in Rats," (Charles River Laboratories, 2000).

66. P. B. Benni, D. MacLeod, K. Ikeda, H.-M. Lin, A validation method for near-infrared spectroscopy based tissue oximeters for cerebral and somatic tissue oxygen saturation measurements. *J. Clin. Monit. Comput.* **32**, 269–284 (2018).

67. J. Klepacki, B. Davari, M. Boulet, R. Lizarraga, U. Christians, A high-throughput HPLC-MS/MS assay for the detection, quantification and simultaneous structural confirmation of 136 drugs and metabolites in human urine. *Ther. Drug Monit.* **39**, 565–574 (2017).

68. R. Avila, C. Li, Y. Xue, J. A. Rogers, Y. Huang, Modeling programmable drug delivery in bioelectronics with electrochemical actuation. *Proc. Natl. Acad. Sci.* **118**, e2026405118 (2021).

69. X. Wang, O. S. Wolfbeis, Optical methods for sensing and imaging oxygen: Materials, spectroscopies and applications. *Chem. Soc. Rev.* **43**, 3666–3761 (2014).

70. W. Lu, W. Bai, H. Zhang, C. Xu, A. M. Chiarelli, A. Vázquez-Guardado, Z. Xie, H. Shen, K. Nandoliya, H. Zhao, K. Lee, Y. Wu, D. Franklin, R. Avila, S. Xu, A. Rwei, M. Han, K. Kwon, Y. Deng, X. Yu, E. B. Thorp, X. Feng, Y. Huang, J. Forbess, Z.-D. Ge, J. A. Rogers, Wireless, implantable catheter-type oximeter designed for cardiac oxygen saturation. *Sci. Adv.* **7**, eabe0579 (2021).

71. H. Zhang, P. Gutruf, K. Meacham, M. C. Montana, X. Zhao, A. M. Chiarelli, A. Vázquez-Guardado, A. Norris, L. Lu, Q. Guo, C. Xu, Y. Wu, H. Zhao, X. Ning, W. Bai, I. Kandela, C. R. Haney, D. Chanda, R. W. Gereau, J. A. Rogers, Wireless, battery-free optoelectronic systems as subdermal implants for local tissue oximetry. *Sci. Adv.* **5**, eaaw0873 (2019).

72. D. A. Benaron, I. H. Parachikov, W.-F. Cheong, S. Friedland, B. E. Rubinsky, D. M. Otten, F. W. H. Liu, C. J. Levinson, A. L. Murphy, J. W. Price, Y. Talmi, J. P. Weersing, J. L. Duckworth, U. B. Hörchner, E. L. Kermit, Design of a visible-light spectroscopy clinical tissue oximeter. *J. Biomed. Opt.* **10**, 044005 (2005).

73. D. Franklin, A. Tzavelis, J. Y. Lee, H. U. Chung, J. Trueb, H. Arafa, S. S. Kwak, I. Huang, Y. Liu, M. Rathod, J. Wu, H. Liu, C. Wu, J. A. Pandit, F. S. Ahmad, P. M. McCarthy, J. A. Rogers, Synchronized wearables for the detection of haemodynamic states via electrocardiography and multispectral photoplethysmography. *Nat. Biomed. Eng.* **7**, 1229–1241 (2023).

74. F. Pizza, M. Biallas, M. Wolf, E. Werth, C. L. Bassetti, Nocturnal cerebral hemodynamics in snorers and in patients with obstructive sleep apnea: A near-infrared spectroscopy study. *Sleep* **33**, 205–210 (2010).

75. C. Davies, J. Y. Lee, J. Walter, D. Kim, L. Yu, J. Park, S. Blake, L. Kalluri, M. Cziraky, E. Stanek, J. Miller, B. J. Harty, J. Schauer, S. M. Rangel, A. Serao, C. Edel, D. S. Ran, M. O.

Olagbenro, A. Lim, K. Gill, J. Cooksey, O. Toloui, T. Power, S. Xu, P. Zee, A single-arm, open-label, multicenter, and comparative study of the ANNE sleep system vs polysomnography to diagnose obstructive sleep apnea. *J. Clin. Sleep Med.* **18**, 2703–2712 (2022).

76. J. Olmo Arroyo, S. Khirani, A. Amaddeo, L. Griffon, L. De Sanctis, P. Pouard, B. Fauroux, A comparison of pulse oximetry and cerebral oxygenation in children with severe sleep apnea-hypopnea syndrome: A pilot study. *J. Sleep Res.* **26**, 799–808 (2017).

77. Z. Zhang, M. Qi, G. Hügli, R. Khatami, Predictors of changes in cerebral perfusion and oxygenation during obstructive sleep apnea. *Sci. Rep.* **11**, 23510 (2021).

78. J. D. Busch, M. Vens, C. Mahler, J. Herrmann, G. Adam, H. Ittrich, Complication rates observed in silicone and polyurethane catheters of totally implanted central venous access devices implanted in the upper arm. *J. Vasc. Interv. Radiol.* **28**, 1177–1183 (2017).
